# Two-dose ChAdOx1 nCoV-19 vaccine protection against COVID-19 hospital admissions and deaths over time: a retrospective, population-based cohort study in Scotland and Brazil

**DOI:** 10.1016/S0140-6736(21)02754-9

**Published:** 2022-01-01

**Authors:** Srinivasa Vittal Katikireddi, Thiago Cerqueira-Silva, Eleftheria Vasileiou, Chris Robertson, Sarah Amele, Jiafeng Pan, Bob Taylor, Viviane Boaventura, Guilherme Loureiro Werneck, Renzo Flores-Ortiz, Utkarsh Agrawal, Annemarie B Docherty, Colin McCowan, Jim McMenamin, Emily Moore, Lewis D Ritchie, Igor Rudan, Syed Ahmar Shah, Ting Shi, Colin R Simpson, Mauricio L Barreto, Vinicius de Araujo Oliveira, Manoel Barral-Netto, Aziz Sheikh

**Affiliations:** a MRC/CSO Social and Public Health Sciences Unit, University of Glasgow, Glasgow, UK; b Public Health Scotland, Glasgow, UK; c Instituto Gonçalo Moniz, Fiocruz, Bahia, Brazil; d Universidade Federal de Bahia, Salvador, Bahia, Brazil; e Usher Institute, University of Edinburgh, Edinburgh, UK; f Department of Mathematics and Statistics, University of Strathclyde, Glasgow, UK; g Departamento de Epidemiologia of Instituto de Medicina Social, Universidade do Estado do Rio de Janeiro, Rio de Janeiro, Brazil; h School of Medicine, University of St Andrews, St Andrews, UK; i Academic Primary Care, University of Aberdeen, Aberdeen, UK; j School of Health, Wellington Faculty of Health, Victoria University of Wellington, New Zealand

## Abstract

**Background:**

Reports suggest that COVID-19 vaccine effectiveness is decreasing, but whether this reflects waning or new SARS-CoV-2 variants—especially delta (B.1.617.2)—is unclear. We investigated the association between time since two doses of ChAdOx1 nCoV-19 vaccine and risk of severe COVID-19 outcomes in Scotland (where delta was dominant), with comparative analyses in Brazil (where delta was uncommon).

**Methods:**

In this retrospective, population-based cohort study in Brazil and Scotland, we linked national databases from the EAVE II study in Scotland; and the COVID-19 Vaccination Campaign, Acute Respiratory Infection Suspected Cases, and Severe Acute Respiratory Infection/Illness datasets in Brazil) for vaccination, laboratory testing, clinical, and mortality data. We defined cohorts of adults (aged ≥18 years) who received two doses of ChAdOx1 nCoV-19 and compared rates of severe COVID-19 outcomes (ie, COVID-19 hospital admission or death) across fortnightly periods, relative to 2–3 weeks after the second dose. Entry to the Scotland cohort started from May 19, 2021, and entry to the Brazil cohort started from Jan 18, 2021. Follow-up in both cohorts was until Oct 25, 2021. Poisson regression was used to estimate rate ratios (RRs) and vaccine effectiveness, with 95% CIs.

**Findings:**

1 972 454 adults received two doses of ChAdOx1 nCoV-19 in Scotland and 42 558 839 in Brazil, with longer follow-up in Scotland because two-dose vaccination began earlier in Scotland than in Brazil. In Scotland, RRs for severe COVID-19 increased to 2·01 (95% CI 1·54–2·62) at 10–11 weeks, 3·01 (2·26–3·99) at 14–15 weeks, and 5·43 (4·00–7·38) at 18–19 weeks after the second dose. The pattern of results was similar in Brazil, with RRs of 2·29 (2·01–2·61) at 10–11 weeks, 3·10 (2·63–3·64) at 14–15 weeks, and 4·71 (3·83–5·78) at 18–19 weeks after the second dose. In Scotland, vaccine effectiveness decreased from 83·7% (95% CI 79·7–87·0) at 2–3 weeks, to 75·9% (72·9–78·6) at 14–15 weeks, and 63·7% (59·6–67·4) at 18–19 weeks after the second dose. In Brazil, vaccine effectiveness decreased from 86·4% (85·4–87·3) at 2–3 weeks, to 59·7% (54·6–64·2) at 14–15 weeks, and 42·2% (32·4–50·6) at 18–19 weeks.

**Interpretation:**

We found waning vaccine protection of ChAdOx1 nCoV-19 against COVID-19 hospital admissions and deaths in both Scotland and Brazil, this becoming evident within three months of the second vaccine dose. Consideration needs to be given to providing booster vaccine doses for people who have received ChAdOx1 nCoV-19.

**Funding:**

UK Research and Innovation (Medical Research Council), Scottish Government, Research and Innovation Industrial Strategy Challenge Fund, Health Data Research UK, Fiocruz, Fazer o Bem Faz Bem Programme; Conselho Nacional de Desenvolvimento Científico e Tecnológico, Fundação Carlos Chagas Filho de Amparo à Pesquisa do Estado do Rio de Janeiro.

**Translation:**

For the Portuguese translation of the abstract see Supplementary Materials section.

## Introduction

Randomised controlled trials and real-world effectiveness studies have shown the considerable short-term protection offered by COVID-19 vaccines against SARS-CoV-2 infection and COVID-19-related hospitalisation and death.^1^, ^2^, ^3^, ^4^ Wide-scale vaccine deployment now forms a central part of the pandemic control strategy in many countries. ChAdOx1 nCoV-19 (Oxford–AstraZeneca in Scotland; Vaxzevria/Fiocruz in Brazil) has been widely deployed in many countries, with its relative affordability and less stringent storage requirements than mRNA vaccines making it particularly suitable for deployment in low-income and middle-income countries.

**Research in context**

**Evidence before this study**

We searched PubMed, medRxiv, and SSRN on Sept 7, 2021, for English language articles using terms related to SARS-CoV-2, COVID-19, vaccination, effectiveness, and waning, with searches updated on Oct 4, 2021. Data from randomised trials and observational studies have suggested decreasing vaccine effectiveness against COVID-19 for BNT162b2 (Pfizer–BioNtech), but results for ChAdOx1 nCoV-19 (Oxford–AstraZeneca) have not yet been published. A previous analysis from the EAVE II Scotland-wide national COVID-19 surveillance platform estimated vaccine effectiveness against infection during the period that the delta (B.1.617.2) variant was most common in Scotland. Vaccine effectiveness of ChAdOx1 nCoV-19 was 73% (95% CI 66–78) for S gene-negative cases and 60% (53–66) for S gene-positive confirmed infections. Immunological data have suggested decreasing antibody levels with time since two-dose vaccination. A preprint of a test-negative design case-control study from Public Health England reported waning effectiveness of ChAdOx1 nCoV-19, but analyses were at risk from biases arising from changes in infection risks over time, selection bias (due to restricting analyses to a minority of severe COVID-19 cases) and depletion of susceptibles bias (where unvaccinated individuals develop immunity over time due to natural infection).

**Added value of this study**

National analyses of data from Scotland and Brazil found risks of severe COVID-19 outcomes (defined as hospital admission or death due to COVID-19) increased with time since receiving a second ChAdOx1 nCoV-19 vaccine dose. Compared with at 2–3 weeks after the second dose, rate ratios for severe COVID-19 at 18–19 weeks were 5·43 (95% CI 4·00–7·38) in Scotland and 4·71 (3·83–5·78) in Brazil. Vaccine effectiveness against both severe COVID-19 and confirmed symptomatic infection diminished over time since receipt of a second dose in both countries. Sensitivity analyses, including a test-negative design case-control study, showed a similar pattern of findings. The consistency of the pattern of findings across the two countries, with differing variants of concern and temporal trends in infection rates, suggests that vaccine waning is a key driver for the increasing numbers of severe outcomes being seen in two-dose ChAdOx1 nCoV-19 vaccinated individuals.

**Implications of all the available evidence**

Protection against COVID-19 symptomatic infection, hospital admissions, and deaths began decreasing within 3 months of second dose ChAdOx1 nCoV-19 vaccination in Scotland and Brazil. There is a need to consider provision of booster doses for people who have been vaccinated with two doses of ChAdOx1 nCoV-19.

Infection rates and severe COVID-19 have increased in several countries that have attained high levels of vaccine coverage.^5^, ^6^ Although this might be attributable to vaccine escape associated with new variants—in particular, delta (B.1.617.2) and to a lesser extent gamma (P.1)^7^, ^8^, ^9^—it is also possible that vaccine effectiveness might be decreasing over time.^10^ Potential vaccine waning has been shown in randomised trials, with neutralising antibody titres decreasing over time,^11^ and diminishing protection against confirmed infection.^12^ Similarly, research indicates BNT162b2 (Pfizer–BioNtech) vaccine boosters offer additional protection over-and-above that achieved by two doses.^6^ However, the clinical and real-world relevance of these findings for ChAdOx1 nCoV-19 remains uncertain.

There have been very high rates of SARS-CoV-2 infection, and COVID-19 hospitalisation and death, in Scotland and Brazil. In both countries, vaccine programmes started early and uptake has been high, with ChAdOx1 nCoV-19 administered with a typical interval of 12 weeks between doses.^13^, ^14^ Both programmes initially targeted people at highest risk of severe disease, with health-care workers and older people prioritised (appendix 2 pp 3–7). Other vaccines have been used in both countries, with no systematic difference in the vaccine administered across demographic groups in Brazil. In Scotland, ChAdOx1 nCoV-19 was initially preferentially delivered to community-dwelling older people and the clinically vulnerable, whereas its use was subsequently restricted to people aged 40 years and older following concerns around haematological and vascular complications, particularly in young people.^15^ Variants of concern have emerged, with delta dominant in Scotland since May, 2021, and gamma common in Brazil since February, 2021.^16^, ^17^

The differing dominant variants across Scotland and Brazil offers the potential to disentangle vaccine waning from the effects of changes in variants; this also offers the opportunity to explore how effectiveness varies in the context of different dominant variants. We, therefore, assessed the association between time since two-dose vaccination with ChAdOx1 nCoV-19 and the risk of severe COVID-19 outcomes (ie, COVID-19 hospital admission or death) in Scotland and Brazil.

## Methods

### Study design

We undertook a retrospective, population-based cohort study to investigate the association between time since two-dose vaccination and COVID-19 outcomes. To assess potential waning, we defined population-based cohorts comprising adults who had received two ChAdOx1 nCoV-19 doses in Scotland and Brazil. This allowed the association between time since receiving a second dose and risk of severe COVID-19 symptoms to be investigated, while minimising potential bias due to apparent waning arising from natural infection among the unvaccinated over time.^18^ To estimate vaccine effectiveness, we defined cohorts that included a comparator of people with no vaccine protection (unvaccinated individuals in Scotland and the earliest follow-up period after first ChAdOx1 nCov-19 dose in Brazil). We did several sensitivity analyses, including a test-negative design case-control study for confirmed infection.

We used data from the EAVE II study, which brings together data from 5·4 million people in Scotland, covering around 99% of the national population (appendix 2 pp 53–54).^19^ Primary care data were linked to laboratory, hospital discharge, death, and vaccination data using a unique identifier.^20^, ^21^ In Brazil, we used three deterministically linked national datasets (appendix 2 pp 55–56): COVID-19 Vaccination Campaign (SI-PNI); Acute Respiratory Infection Suspected Cases (e-SUS-Notifica), which holds clinical and laboratory data from all suspected cases and contact tracing; and Severe Acute Respiratory Infection/Illness (SIVEP-Gripe), which includes all COVID-19 hospitalisations and deaths.

In both countries, we first defined cohorts of two-dose vaccinated people through vaccination records. We excluded children (younger than 18 years), and people who had received any vaccine other than ChAdOx1 nCoV-19, had inconsistent vaccination records (eg, received different vaccine types, or had an interval of <14 days between doses), or had diagnosed previous infection. For estimating vaccine effectiveness, differences in the underlying databases precluded identical approaches for cohort definition. In Scotland, data were available for the entire population, including unvaccinated people. In Brazil, data were only available for individuals who had received a vaccine. To investigate vaccine effectiveness, we studied a cohort of all adults alive in Scotland from May 19, 2021 (when >50% of cases were delta; appendix 2 p 52), with follow-up until Oct 25, 2021. In Brazil, we studied a cohort for which entry started on the date of receiving the first ChAdOx1 nCoV-19 dose (from Jan 18 to Oct 25, 2021).

We followed the STROBE and RECORD reporting guidelines (appendix 2 pp 46–51).^22^, ^23^ The statistical analysis plan was published before we did the analysis.^24^

For Scotland, ethics approvals were obtained from the National Research Ethics Service Committee, Southeast Scotland 02 (reference number 12/SS/0201), and Public Benefit and Privacy Panel for Health and Social Care (reference number 1920-0279). For Brazil, the Brazilian National Commission in Research Ethics approved the research protocol (CONEP approval number 4.921.308).

### Exposures and confounders

To assess waning, we compared rates of severe COVID-19 for the reference period of 2–3 weeks inclusive (ie, 14–27 days) from the date of the second dose among the two-dose vaccinated cohort^25^, ^26^ with subsequent fortnightly periods in both Scotland and Brazil. We followed up individuals until they experienced the primary outcome (a composite of COVID-19 hospitalisation or COVID-19 death), death, receipt of another vaccine type, or end of follow-up (Oct 25, 2021, in both countries).

To assess vaccine effectiveness, we classified exposure periods as time-varying. In Scotland, categories were unvaccinated, first-dose control period (0–13 days after first dose), first dose protection (from 14 days after first dose until receipt of a second dose), second dose control period (0–13 days after second dose), and then fortnightly periods thereafter. In Brazil, the same categories were used except that data on the unvaccinated population were not available. The unvaccinated period was the reference group in Scotland, given the artificially low risk seen immediately after the first dose because individuals with COVID-19 symptoms were advised not to attend for vaccination.^2^ The 0–13 days post-first dose period was the reference group in Brazil.

To reduce potential confounding by changes in variants, we restricted analyses to the period when delta comprised most cases in Scotland (ie, May 19, 2021, onwards; appendix 2 p 52).^16^

The following confounders were adjusted for in both countries: age (5-year bands), sex, socioeconomic position measured by quintiles of deprivation (the Scottish Index of Multiple Deprivation in Scotland and the Índice Brasileiro de Privação in Brazil), calendar week (as categorical), and interval between doses. In Scotland, we additionally adjusted for number and types of comorbidities commonly associated with COVID-19 illness (appendix 2 p 10),^27^ body-mass index, and the number of previous SARS-CoV-2 tests (a marker of being in a high-risk occupational group), which were identified from general practitioner records in the 5-year period before December, 2020. In Brazil, macroregion of residence (with interactions between age and time period) and primary reason for vaccination were also adjusted for.

### Outcomes

The primary prespecified outcome was severe COVID-19, defined as COVID-19 hospital admission or death (appendix 2 p 45).^2^ Secondary outcomes were the individual outcomes of COVID-19 hospital admission, death, and confirmed symptomatic SARS-CoV-2 infection. In Scotland, diagnostic testing was based on RT-PCR; whereas, in Brazil, rapid antigen testing or RT-PCR was used.

### Statistical analysis

To assess potential waning, we used Poisson regression to estimate rate ratios (RRs) with 95% CIs in the two-dose vaccinated cohorts, with natural logarithmic of person-years as offset. To test the hypothesis of whether waning occurred, we used two different approaches. First, we looked for statistical evidence of reducing effectiveness by doing a trend test of RRs on the period after vaccination from 2–3 weeks onwards. Second, we assessed whether effectiveness achieved and maintained a minimum acceptable level, adopting the US Food and Drug Administration threshold of achieving a minimum vaccine effectiveness of 50% for the point estimate.^28^, ^29^

To estimate vaccine effectiveness, Poisson regression was used to compare rates for different post-vaccination periods with the unvaccinated group (in Scotland) or the 0–13 days after the first dose period (in Brazil). Vaccine effectiveness was calculated as (1 – RR) × 100 from models including comparators with no vaccine protection. Analyses adjusted for the same confounders. Formulae for statistical models are in appendix 2 (p 38).

In Scotland, we applied sampling weights to account for over-representation of some populations (men and women aged 18–40 years), which was a consequence of those registered at multiple general practitioner practices, visitors, and those having left Scotland.^2^ These sampling weights were calculated on the basis of the 2020 National Records of Scotland mid-year population estimates in Scotland. All study participants who had contact with vaccinations, COVID-19 testing, hospital admissions, or who died during the period from March 1, 2020, to the end of follow-up were assigned a weight of 1.

Subgroup analyses were conducted to explore potential differences by age group (18–64, 65–79, and ≥80 years).

We conducted several sensitivity analyses. As confirmed symptomatic infection is susceptible to bias due to differential ascertainment, we additionally investigated this outcome using a test-negative design. We defined cases as those who had tested positive and had been symptomatic. We randomly sampled one symptomatic control negative test per case for that period and estimated odds ratios for different vaccination histories, adjusting for age, sex, temporal trend, geography, and deprivation using generalised additive logistic regression. Temporal trend was estimated using the time elapsed between the study start and date of test; temporal trend and age were modelled as smooth functions. We additionally adjusted for the number of at-risk groups, smoking status, blood pressure, body-mass index, and health board in Scotland, and specific comorbidities (diabetes, obesity, chronic kidney disease, cardiac disease, pregnancy, and post-partum period) in Brazil.

In Scotland, it was possible that COVID-19 might be incidentally diagnosed (eg, among people admitted to hospital for another reason). Therefore, we repeated the main analyses using outcomes ascertained only through pillar 2 testing. The main analyses were also repeated for the narrower age group of 40–64 years, given the restricted use of ChAdOx1 nCoV-19 since May 7, 2021, in Scotland. It is possible that very rapid changes in infection risk could result in residual confounding that has not been accounted for by adjustment for 1-week calendar periods within the cohort analysis. Therefore, a post-hoc incidence-density matched case-control study was conducted for the primary outcome, with exact matching (with ten controls per case) for outcome date, sex, age, and local authority, and statistical adjustment for further covariates. Therefore, cases were defined by having had a COVID-19 hospital admission or death whereas controls had not had this outcome by the date of matching.

For Brazilian analyses, we did a sensitivity analysis to mitigate potential confounding by the emergence of the delta variant (appendix 2 p 57) by restricting the study period up to July 31, 2021. To explore the potential implications of bias arising from the absence of comorbidity information in Brazil, we repeated the main analysis (≥14 days after second dose among the two-dose ChAdOx1 nCoV-19 cohort) excluding covariates for comorbidities in Scotland. Lastly, we also conducted analyses using the same reference period for vaccine effectiveness estimates in Scotland as in Brazil (ie, 0–13 days after first dose vaccination) to explore potential for bias of vaccine effectiveness estimates in Brazil.

All analyses were carried out using R statistical software (versions 4.1.1 and 3.6.1).

### Role of the funding source

The funder of the study had no role in study design, data collection, data analysis, data interpretation, or the writing of the report.

## Results

1 972 454 adults received two doses of ChAdOx1 nCoV-19 in Scotland and 42 558 839 in Brazil (table 1; appendix 2 pp 59–60, 65). The number and rate of confirmed symptomatic infections and severe COVID-19 cases (including hospital admissions and deaths ≥14 days after the second dose) among the two-dose ChAdOx1 nCoV-19 cohort in Scotland and in Brazil are shown in table 1. In Scotland, peaks of severe COVID-19 disease and confirmed SARS-CoV-2 infection were observed in July and September, 2021 (appendix 2 pp 61–62), whereas in Brazil, peaks occurred earlier in March and June, 2021 (appendix 2 pp 66–67). Two-dose vaccination in Brazil occurred later than in Scotland (appendix 2 pp 58, 68); therefore, a shorter follow-up duration was possible.

**Table 1:** Population characteristics for cohorts analysed in Scotland and Brazil

		**Scotland**		**Brazil**	
		Two dose cohort	Vaccine effectiveness cohort*	Two dose cohort	Vaccine effectiveness cohort*
Total		1 972 454	2 534 527	42 558 839	56 013 638
Sex					
	Female	997 890 (50·6%)	1 269 011 (50·1%)	23 006 854 (54·1%)	29 683 170 (53·0%)
	Male	974 564 (49·4%)	1 265 517 (49·9%)	19 551 985 (45·9%)	26 330 468 (47·0%)
Age, years					
	Mean (SD)	58 (15·3)	52 (17·7)	50 (14·0)	48 (14·6)
	Median (IQR)	57 (48–68)	53 (40–63)	51 (40–60)	49 (38–59)
	18–64	1 392 123 (70·6%)	1 898 322 (74·9%)	37 676 032 (88·5%)	50 410 302 (90·0%)
	65–79	376 286 (19·1%)	411 685 (16·2%)	3 355 384 (7·9%)	3 816 360 (6·8%)
	≥80	204 045 (10·3%)	224 520 (8·9%)	1 527 423 (3·6%)	1 786 976 (3·2%)
Vaccination month†					
	Unvaccinated	..	503 455 (19·9%)	..	..
	December, 2020	..	12 (0%)	..	..
	January, 2021	7 (<0·1%)	221 953 (8·8%)	..	31 188 (0·1%)
	February, 2021	1587 (0·1%)	546 523 (21·6%)	523 (<0·1%)	121 097 (0·2%)
	March, 2021	102 109 (5·2%)	803 902 (31·7%)	6290 (<0·1%)	245 321 (0·4%)
	April, 2021	489 091 (24·8%)	202 752 (8·0%)	841 673 (2·0%)	1 377 361 (2·5%)
	May, 2021	604 299 (30·6%)	215 733 (8·5%)	1 689 814 (4·0%)	2 762 162 (4·9%)
	June, 2021	505 233 (25·6%)	25 011 (1·0%)	1 986 489 (4·7%)	4 517 705 (8·1%)
	July, 2021	220 483 (11·2%)	7994 (0·3%)	8 786 235 (20·6%)	14 003 567 (25·0%)
	August, 2021	34 689 (1·8%)	3948 (0·2%)	12 308 438 (28·9%)	15 256 542 (27·2%)
	September, 2021	10 724 (0·5%)	2548 (0·1%)	12 113 808 (28·5%)	12 717 568 (22·7%)
	October, 2021	4232 (0·2%)	697 (<0·1%)	4 825 569 (11·3%)	4 981 127 (8·9%)
Interval between doses, weeks					
	Unvaccinated	..	503 455 (19·9%)	..	..
	One dose only	..	58 375 (2·3%)	..	13 014 057 (23·2%)
	<7 weeks	27 121 (1·4%)	27 122 (1·1%)	292 142 (0·7%)	292 508 (0·5%)
	7–8 weeks	360 728 (18·3%)	360 796 (14·2%)	952 852 (2·2%)	957 090 (1·7%)
	9–10 weeks	1 020 620 (51·7%)	1 020 637 (40·3%)	4 106 531 (9·6%)	4 134 102 (7·3%)
	11–12 weeks	492 722 (25·0%)	492 727 (19·4%)	25 419 256 (59·7%)	25 649 224 (45·4%)
	≥13 weeks	71 263 (3·6%)	71 416 (2·8%)	11 788 058 (27·7%)	11 966 657 (21·9%)
Deprivation status quintile‡					
	1	350 922 (17·8%)	516 156 (20·4%)	8 962 383 (21·1%)	10 841 567 (19·4%)
	2	382 783 (19·4%)	506 194 (20·0%)	8 034 607 (18·9%)	10 042 918 (17·9%)
	3	406 927 (20·6%)	507 642 (20·0%)	8 794 837 (20·7%)	11 354 054 (20·3%)
	4	412 754 (20·9%)	495 809 (19·6%)	8 570 045 (20·1%)	11 531 844 (20·6%)
	5	408 903 (20·7%)	490 143 (19·3%)	7 981 107 (18·8%)	11 937 518 (21·3%)
	Unknown	10 165 (0·5%)	18 584 (0·7%)	215 860 (0·5%)	305 737 (0·5%)
COVID-19 outcomes, n (rate per 100 000 person-years)					
	COVID-19 hospital admission or death	4662 (236·4)	7211 (288·7)	9039 (113·6)	68 763 (318·0)
	COVID-19 hospital admission or death ≥14 days after second dose	4494 (227·8)	..	6508 (101·8)	..
	COVID-19 hospital admission	4355 (220·8)	6830 (273·4)	8927 (110·8)	68 494 (316·7)
	COVID-19 hospital admission ≥14 days after second dose	4188 (212·3)	..	6436 (99·4)	..
	COVID-19 death after second dose	916 (46·4)	1192 (47·7)	3238 (40·7)	21 973 (101·6)
	COVID-19 death ≥14 days after second dose	911 (46·2)	..	2360 (37·0)	..
	COVID-19 confirmed infection	95 330 (4833·0)	154 402 (6181·4)	103 755 (1280·4)	638 588 (2866·4)
	COVID-19 confirmed infection ≥14 days after second dose	92 133 (4670·0)	..	74 974 (1158·1)	..

Information about additional covariates is included within appendix 2 (pp 8–10).

* Cohorts used to assess vaccine effectiveness differed between Scotland and Brazil: in Scotland, the availability of data on the entire unvaccinated population allowed analyses to be based on the resident adult population, whereas in Brazil, vaccine effectiveness was assessed among the cohort of individuals who had received at least one dose of ChAdOx1 nCoV-19.

† Vaccine month is for the second dose for the rate ratio cohort and first dose for the vaccine effectiveness cohort.

‡ Deprivation status was measured by quintiles of the Index of Multiple Deprivation 2020 for Scotland and Indice Brasileiro de Privacao at the municipality level for Brazil; 1 is the lowest quintile of deprivation, 5 is the highest quintile of deprivation.

**Figure 1:**
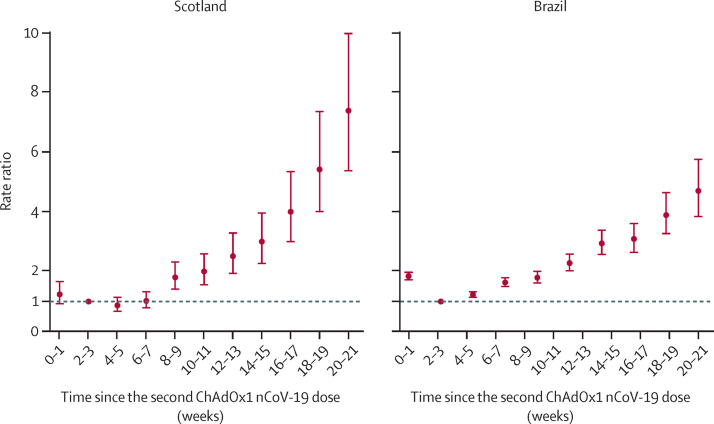
Rate ratios for time since receiving two doses of ChAdOx1 nCoV-19 and severe COVID-19 (hospital admission or death) in Scotland and Brazil Analyses in Scotland were adjusted for age, sex, deprivation, comorbidities, number of previous tests, interval between doses, and temporal trend. Analyses in Brazil were adjusted for age, sex, deprivation, macroregion of residence, primary reason for vaccination, interval between doses, and temporal trend. Error bars are 95% CIs.

RRs for severe COVID-19 increased with time since receiving two doses of ChAdOx1 nCoV-19 in Scotland and Brazil (figure 1; appendix 2 pp 16, 22). In Scotland, RRs increased to 2·01 (95% CI 1·54–2·62) at 10–11 weeks, 3·01 (2·26–3·99) at 14–15 weeks, and 5·43 (4·00–7·38) at 18–19 weeks, compared with at 2–3 weeks after the second dose. The pattern of results was similar in Brazil, with RRs of 2·29 (95% CI 2·01–2·61) at 10–11 weeks, 3·10 (2·63–3·64) at 14–15 weeks, and 4·71 (3·83–5·78) at 18–19 weeks, compared with at 2–3 weeks. Waning was statistically significant in both countries (p<0·0001; appendix 2 p 20).

In Scotland, vaccine effectiveness (estimated by comparing risks of severe COVID-19 in the two-dose vaccinated group with the unvaccinated group) initially remained relatively stable between 2–3 weeks and 6–7 weeks after the second dose, then decreased up to 18–19 weeks after the second dose (table 2). In Brazil, vaccine effectiveness (estimated by comparing risks with the 0–1 week period following a first dose, which might underestimate protection) was generally lower than in Scotland, increasing up to 4–5 weeks after the second dose, then decreasing up to 18–19 weeks (table 2).

**Table 2:** Vaccine effectiveness estimates for ChAdOx1 nCoV-19 against COVID-19 hospital admissions or death by length of time since two-dose vaccination in Scotland and Brazil

	**Scotland**			**Brazil**		
	Person-years	Number of events	Vaccine effectiveness* (95% CI)	Person-years	Number of events	Vaccine effectiveness* (95% CI)
Unvaccinated	336 942	2245	0% (ref)	..	..	..
0–2 weeks after first dose	6860	39	−15·4% (−60·6 to 17·0)	1 849 099	21 736	0% (ref)
Partially vaccinated†	94 761	420	49·3% (43·3 to 54·6)	11 701 310	37 802	57·9% (56·9 to 58·9)
0–1 week after second dose	47 252	78	77·7% (71·9 to 82·3)	1 601 585	2688	73·2% (71·9 to 74·5)
2–3 weeks after second dose	55 318	85	83·7% (79·7 to 87·0)	1 492 259	1095	86·4% (85·4 to 87·3)
4–5 weeks after second dose	65 698	106	86·6% (83·6 to 89·0)	1 338 063	1019	83·5% (82·3 to 84·7)
6–7 weeks after second dose	71 120	134	86·8% (84·2 to 88·9)	1 117 983	1019	77·9% (76·1 to 79·5)
8–9 weeks after second dose	73 540	245	79·0% (75·9 to 81·7)	862 976	863	75·6% (73·4 to 77·6)
10–11 weeks after second dose	73 212	280	79·6% (76·8 to 82·1)	651 213	751	69·3% (66·3 to 72·1)
12–13 weeks after second dose	71 773	337	77·4% (74·6 to 80·0)	445 924	646	60·8% (56·6 to 64·6)
14–15 weeks after second dose	68 114	356	75·9% (72·9 to 78·6)	264 128	472	59·7% (54·6 to 64·2)
16–17 weeks after second dose	63 974	402	70·5% (67·0 to 73·7)	169 692	397	50·5% (43·4 to 56·6)
18–19 weeks after second dose	58 608	508	63·7% (59·6 to 67·4)	132 459	275	42·2% (32·4 to 50·6)
20–21 weeks after second dose	45 716	598	53·6% (48·4 to 58·3)	..	..	..

Scotland reference group: unvaccinated, Brazil reference group: 0–13 days after first dose vaccination.

* In Scotland, vaccine effectiveness was adjusted for age, sex, deprivation, comorbidities, number of previous tests, interval between doses, and temporal trend; individuals positive for SARS-CoV-2 before Dec 8, 2020, were excluded from the analysis. In Brazil, vaccine effectiveness was adjusted for age, sex, deprivation, macroregion of residence, primary reason for vaccination, interval between doses, and temporal trend.

† Partially vaccinated: ≥2 weeks after the first dose and before the second dose.

When investigated as separate outcomes, patterns for COVID-19 hospital admissions and COVID-19 deaths were broadly similar in both Scotland (appendix 2 p 21) and Brazil (appendix 2 pp 22–24) as for the primary composite outcome, although estimates for death were less precise.

**Figure 2:**
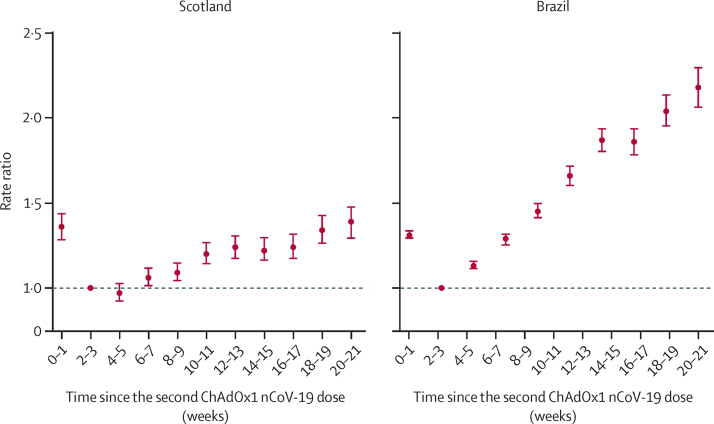
Rate ratios for time since receiving two doses of ChAdOx1 nCoV-19 and confirmed SARS-CoV-2 symptomatic infection in Scotland and Brazil Analyses in Scotland were adjusted for age, sex, deprivation, comorbidities, number of previous tests, interval between doses, and temporal trend. Analyses in Brazil were adjusted for age, sex, deprivation, macroregion of residence, primary reason for vaccination, interval between doses, and temporal trend. Error bars are 95% CIs.

RRs increased over time for confirmed symptomatic SARS-CoV-2 infection in both countries, but to a lesser extent than for severe COVID-19 (figure 2; appendix 2 pp 18–19, 25–26). In Scotland, RRs increased to 1·20 (95% CI 1·14–1·27) at 10–11 weeks, 1·22 (1·16–1·30) at 14–15 weeks, and 1·34 (1·26–1·43) at 18–19 weeks, compared with at 2–3 weeks. In Brazil, RRs for infection were 1·66 (95% CI 1·60–1·72) at 10–11 weeks, 1·86 (1·78–1·94) at 14–15 weeks, and 2·18 (2·06–2·30) at 18–19 weeks, compared with at 2–3 weeks (figure 2; appendix 2 pp 25–26).

Subgroup analyses for the primary outcome of severe COVID-19 were difficult to reliably estimate in both Scotland and Brazil because the rapid delivery of vaccination among older groups led to a high level of collinearity between calendar period, dose interval, and length of time since receipt of a second dose (appendix 2 pp 63–64). In Scotland, RRs increased over time for the group aged 18–64 years, but showed no clear pattern among both older groups, although results were based on few events (appendix 2 pp 16–17). By contrast, RRs in Brazil were greater in the 65–79 years age group than in the 18–64 years age group (appendix 2 pp 22–23).

Sensitivity analyses to explore potential ascertainment biases led to broadly similar patterns of findings as the main results. In Scotland, cohort analyses limited to pillar 2 testing, restricted to the 40–64 year age group, and using an incidence-density matched case-control design showed similar results as the main analysis (appendix 2 pp 34–35, 38). In Scotland, vaccine effectiveness against confirmed SARS-CoV-2 symptomatic infection in the test-negative design case-control study decreased between 2–3 weeks and 18–19 weeks after the second dose (table 3). In test-negative design analyses for Brazil, vaccine effectiveness also decreased after 2–3 weeks, but was generally slightly higher than in Scotland, potentially due to differences in circulating variants (table 3). In Brazil, sensitivity analyses with follow-up restricted to July 31, 2021 (before the delta variant became established) yielded similar findings as the main results (appendix 2 p 27). Repeating vaccine effectiveness analyses for Scotland using the same reference period and more limited adjustment as in Brazil suggested that vaccine effectiveness is likely to be consistently under-estimated in Brazil (appendix 2 p 35).

**Table 3:** Vaccine effectiveness estimates for ChAdOx1 nCoV-19 against confirmed SARS-CoV-2 symptomatic infection by length of time since two-dose vaccination in Scotland and Brazil using a test-negative design case-control study

	**Scotland**			**Brazil**		
	Total samples	Positive samples	Vaccine effectiveness* (95% CI)	Total samples	Positive samples	Vaccine effectiveness* (95% CI)
Unvaccinated	26 130	13 698	0% (ref)	9 852 053	4 920 001	0% (ref)
0–1 week after first dose	911	374	20·9% (8·2 to 31·9)	286 322	151 328	−9·6% (−10·5 to −8·8)
Partially vaccinated†	15 714	7176	37·6% (34·6 to 40·5)	1 143 423	398 717	37·6% (37·3 to 37·9)
0–1 week after second dose	5027	2025	50·2% (46·7 to 53·5)	112 391	30 550	51·3% (50·6 to 52·0)
2–3 weeks after second dose	7141	2429	67·9% (65·9 to 69·8)	95 671	7963	69·8% (69·3 to 70·4)
4–5 weeks after second dose	8947	3387	67·3% (65·3 to 69·1)	79 298	15 568	68·4% (67·8 to 68·9)
6–7 weeks after second dose	10 622	4346	63·8% (61·7 to 65·7)	60 301	12 401	66·8% (66·1 to 67·5)
8–9 weeks after second dose	11 258	4633	63·3% (61·3 to 65·3)	44 351	9424	65·4% (64·6 to 66·2)
10–11 weeks after second dose	14 043	6319	59·3% (57·2 to 61·4)	32 832	7103	63·2% (62·2 to 64·2)
12–13 weeks after second dose	17 300	7966	55·3% (53·0 to 57·5)	22 454	5177	58·8% (57·4 to 60·1)
14–15 weeks after second dose	17 421	7670	52·9% (50·4 to 55·2)	15 305	3435	59·8% (58·2 to 61·4)
16–17 weeks after second dose	15 442	6554	48·7% (45·9 to 51·4)	10 822	2529	58·7% (56·7 to 60·5)
18–19 weeks after second dose	14 403	6248	44·6% (41·5 to 47·6)	7458	1852	57·7% (55·4 to 60·0)
20–21 weeks after second dose	10 596	4718	39·1% (35·4 to 42·6)	..	..	..

* In Scotland, vaccine effectiveness was adjusted for age, sex, deprivation, comorbidities, number of at-risk groups, smoking status, blood pressure, body-mass index, health board, interval between doses, and temporal trend. In Brazil, vaccine effectiveness was adjusted for age, sex, deprivation, macroregion of residence, diabetes, obesity, immunosuppression, cardiac disease, pregnancy, puerperal period, chronic kidney disease, and temporal trend. Descriptive characteristics for the sample are available in appendix 2 (pp 11–15).

† Partially vaccinated: ≥2 weeks after the first dose and before the second dose.

## Discussion

Risks of severe COVID-19 increased over a relatively short follow-up duration following second doses of ChAdOx1 nCoV-19 in Scotland and Brazil, indicating waning vaccine effectiveness. In comparison to the period of greatest protection (2–3 weeks after the second dose), RRs for severe COVID-19 increased to 5·43 (95% CI 4·00–7·38) in Scotland and 4·71 (3·83–5·78) in Brazil at 18–19 weeks. Comparative analyses across both countries with different dominant variants of concern suggests that the findings are unlikely to be accounted for by confounding due to the emergence of the delta variant or trends in infection rates. Quantifying the exact magnitude of waning is challenging, and vaccine effectiveness estimates should be considered with caution given the difficulty of estimating risk among unvaccinated people. However, our findings consistently show substantial waning in both countries.

Randomised trials for ChAdOx1 nCoV-19 have shown short-term effective protection against severe COVID-19 disease;^1^, ^30^ however, research on potential vaccine waning has mainly focused on BNT162b2. Results from a randomised trial showed that vaccine effectiveness against confirmed infection for BNT162b2 reduced from 96·2% (95% CI 93·3–98·1) at 7 days to less than 2 months after the second dose, to 83·7% (74·7–89·9) at 4 to 6 months,^12^ and recent real-world data showed protection against severe disease was maintained up to 6 months.^31^ In Israel, risk of severe COVID-19 among fully vaccinated adults aged 65 years and older was 1·7 times (95% CI 1·0–2·7) higher for those vaccinated in January, 2021, than for those vaccinated in March, 2021.^32^ Another study assessing a representative sample of the UK population assessed the association between time since second dose of both ChAdOx1 nCoV-19 and BNT162b2 and new RT-PCR confirmed infections.^33^ The findings showed waning protection among 18–64-year-olds with BNT162b2, whereas ChAdOx1 nCoV-19 estimates were imprecise, but suggested a downward trend.^34^ Meanwhile, immunological markers such as spike-antibody levels appear to fall over a 3–10-week period after the second dose of ChAdOx1 nCoV-19 and BNT162b2 in a population-based study of 552 participants in England and Wales.^34^ A test-negative design study (preprint) by Public Health England has suggested waning protection against symptomatic SARS-CoV-2 infection and severe COVID-19, particularly for ChAdOx1 nCoV-19, with vaccine effectiveness estimates for symptomatic infection generally similar to our test-negative design results.^35^ However, in the Public Health England study higher vaccine effectiveness for COVID-19 hospitalisations and deaths was observed, although the results were limited to pillar 2 tests, which tend to exclude the most severe cases, especially among people at higher risk.

Assessment of vaccine waning from observational data is methodologically challenging due to the closely inter-related nature of key variables. Most countries initially prioritised delivery to people at highest risk of severe disease, including older people and people with comorbidities, meaning these groups have longer follow-up. New SARS-CoV-2 variants have emerged, with increasing risks of severe disease among vaccinated people related to the delta variant.^17^, ^36^, ^37^, ^38^ However, the longest time since vaccination will be in the most recent months when outcomes might deteriorate due to new variants. The length of time between doses can affect immune responses,^39^ and also changed in many countries over time. Detection of vaccine waning can also be particularly difficult if conducted in settings with low infection rates.

By conducting harmonised analyses in contexts with different circulating variants, our study has important strengths that help mitigate these issues. Drawing on data from Scotland and Brazil makes results less prone to confounding by changes in viral variants or other secular trends. In Scotland, we restricted analyses to when the delta variant was dominant, whereas in Brazil, the gamma variant was common. Vaccine delivery to population subgroups differed across the two countries. In Scotland, ChAdOx1 nCoV-19 was mainly administered to older people residing outside of care homes.^2^ However, in Brazil, ChAdOx1 nCoV-19 was not targeted at particular demographic groups, making age comparisons potentially more reliable, but potentially making vaccine effectiveness estimates less comparable across countries.^14^ Additional strengths include using national databases to assess clinically important endpoints, and a high level of statistical power. Focusing on the two-dose vaccinated population helped to avoid potential bias arising from comparisons with unvaccinated people becoming immune over time through natural infection, the so-called depletion of susceptibles bias.^18^

Several limitations should be noted. First, given the highly correlated nature of time since vaccination, calendar time (including circulating variants), and interval between doses, residual confounding remains possible. The prioritisation of older people for early vaccination makes distinguishing between these factors particularly difficult for subgroup analyses, especially in Scotland where high uptake among older people was achieved over a few weeks. Therefore, estimates among older age groups are potentially less reliable in Scotland. Second, there was no information on some important confounders. However, analyses in Scotland benefited from richer covariate data. Furthermore, data were not available for unvaccinated individuals within Brazil, meaning vaccine effectiveness estimates were based on the early unvaccinated period and symptom onset used for more accurate estimation. Therefore, vaccine effectiveness estimates might differ between Scotland and Brazil due to these methodological differences. Our sensitivity analyses in Scotland suggested the Brazilian results are likely to underestimate vaccine effectiveness at baseline and lead to the magnitude of vaccine effectiveness waning being overestimated. Therefore, we can be more confident that important waning is occurring than quantifying its exact magnitude. Third, underascertainment of outcomes is possible, particularly for infection. We believe this is less likely for hospital admissions and deaths, with both countries having long-standing robust data infrastructure systems. Our test-negative design analysis provides reassurance as to the robustness of our findings for confirmed infection. However, although we excluded individuals who had previous confirmed infection from our analyses, undetected infection and its resultant immunity could nevertheless bias our vaccine effectiveness estimates. Fourth, we did not have access to individual-level information on SARS-CoV-2 variants (except for a very small minority of the population). Although confounding by variant is unlikely in Scotland because nearly all cases were due to delta,^16^, ^40^ the absence of this information prevents more nuanced assessment of how waning differs across variants, especially given the above methodological differences across countries. Fifth, following our prespecified analysis plan and on the basis of published guidance,^41^ we did not correct p values for multiple testing. However, we note that statistical tests for waning would have readily met conventional tests for statistical significance if Bonferroni correction was applied. Lastly, Scotland and Brazil have had high infection rates during the period of study compared with other countries. Therefore, our estimates of the magnitude of vaccine effectiveness waning might not be generalisable to countries with low infection rates where waning might be more modest.

Our findings have important implications for vaccination policy. In combination with the emerging immunological and trials data suggesting that vaccine effectiveness declines over time following two-dose vaccination,^12^, ^42^ our findings highlight the need to consider providing booster doses. Further evidence in support of booster doses comes from real-world data from Israel, which found that BNT162b2 boosters were associated with reduced severe COVID-19.^6^ However, issues of global equity in vaccine supply remain a concern.

In conclusion, our findings suggest that ChAdOx1 nCoV-19 vaccine protection against severe COVID-19 wanes within a few months of the second vaccine dose. Consideration should be given to provision of booster doses for those administered ChAdOx1 nCoV-19.

## Data sharing

A data dictionary covering the datasets used in this study can be found at https://github.com/EAVE-II/EAVE-II-data-dictionary. All statistical code used in this study is publicly available at https://github.com/EAVE-II/Covid-VE. The data used in this study are sensitive and will not be made publicly available.

## Declaration of interests

SVK is a member of the UK Government's Scientific Advisory Group on Emergencies subgroup on ethnicity, the Cabinet Office's International Best Practice Advisory Group, and was co-chair of the Scottish Government's Expert Reference Group on Ethnicity and COVID-19. CR reports grants from the Medical Research Council (MRC) and Public Health Scotland, during the conduct of the study, and is a member of the Scottish Government Chief Medical Officer's COVID-19 Advisory Group, Scientific Pandemic Influenza Group on Modelling, and Medicines and Healthcare products Regulatory Agency Vaccine Benefit and Risk Working Group. AS is a member of the Scottish Government Chief Medical Officer's COVID-19 Advisory Group and its Standing Committee on Pandemics; he is also a member of the UK Government's New and Emerging Respiratory Virus Threats Risk Stratification Subgroup and a member of AstraZeneca's Thrombotic Thrombocytopenic Taskforce. All roles are unremunerated. VdAO, VB, MLB, and MB-N are employees of Fiocruz, a federal public institution, which manufactures Vaxzevria in Brazil, through a full technology transfer agreement with AstraZeneca. Fiocruz allocates all its manufactured products to the Ministry of Health for the public health service use. All other authors declare no competing interests.

## Supplementary Materials

Portuguese translation of the abstract

## References

[1] Voysey M, Clemens SAC, Madhi SA (2021). Safety and efficacy of the ChAdOx1 nCoV-19 vaccine (AZD1222) against SARS-CoV-2: an interim analysis of four randomised controlled trials in Brazil, South Africa, and the UK. Lancet.

[2] Vasileiou E, Simpson CR, Shi T (2021). Interim findings from first-dose mass COVID-19 vaccination roll-out and COVID-19 hospital admissions in Scotland: a national prospective cohort study. Lancet.

[3] Dagan N, Barda N, Kepten E (2021). BNT162b2 mRNA Covid-19 vaccine in a nationwide mass vaccination setting. N Engl J Med.

[4] Shah ASV, Gribben C, Bishop J (2021). Effect of vaccination on transmission of SARS-CoV-2. N Engl J Med.

[5] WHO (2021). WHO coronavirus (COVID-19) dashboard. https://covid19.who.int/.

[6] Bar-On YM, Goldberg Y, Mandel M (2021). Protection of BNT162b2 vaccine booster against COVID-19 in Israel. N Engl J Med.

[7] Abu-Raddad LJ, Chemaitelly H, Butt AA (2021). Effectiveness of the BNT162b2 COVID-19 vaccine against the B.1.1.7 and B.1.351 variants. N Engl J Med.

[8] Skowronski DM, Setayeshgar S, Zou M (2021). Single-dose mRNA vaccine effectiveness against SARS-CoV-2, including alpha and gamma variants: a test-negative design in adults 70 years and older in British Columbia, Canada. Clin Infect Dis.

[9] Krause PR, Fleming TR, Longini IM (2021). SARS-CoV-2 variants and vaccines. N Engl J Med.

[10] Keehner J, Horton LE, Binkin NJ (2021). Resurgence of SARS-CoV-2 infection in a highly vaccinated health system workforce. N Engl J Med.

[11] Flaxman A, Marchevsky NG, Jenkin D (2021). Reactogenicity and immunogenicity after a late second dose or a third dose of ChAdOx1 nCoV-19 in the UK: a substudy of two randomised controlled trials (COV001 and COV002). Lancet.

[12] Thomas SJ, Moreira ED, Kitchin N (2021). Safety and efficacy of the BNT162b2 mRNA COVID-19 vaccine through 6 months. N Engl J Med.

[13] Scottish Government (2021). Coronavirus (COVID-19) vaccination. https://www.gov.scot/collections/coronavirus-covid-19-vaccination/#vaccinationprogramme.

[14] Ministro da Saúde (2021). Plano Nacional de Operacionalização da Vacinação contra a COVID-19. Brasilia. https://www.gov.br/saude/pt-br/coronavirus/vacinas/plano-nacional-de-operacionalizacao-da-vacina-contra-a-covid-19.

[15] Simpson CR, Shi T, Vasileiou E (2021). First-dose ChAdOx1 and BNT162b2 COVID-19 vaccines and thrombocytopenic, thromboembolic and hemorrhagic events in Scotland. Nat Med.

[16] Sheikh A, McMenamin J, Taylor B, Robertson C (2021). SARS-CoV-2 delta VOC in Scotland: demographics, risk of hospital admission, and vaccine effectiveness. Lancet.

[17] Fiocruz RG (2021). Frequência das Principais Linhagens do SARS-CoV-2 por mês de amostragem. http://www.genomahcov.fiocruz.br/frequencia-das-principais-linhagens-do-sars-cov-2-por-mes-de-amostragem/.

[18] Lipsitch M, Goldstein E, Ray GT, Fireman B (2019). Depletion-of-susceptibles bias in influenza vaccine waning studies: how to ensure robust results. Epidemiol Infect.

[19] Simpson CR, Robertson C, Vasileiou E (2020). Early pandemic evaluation and enhanced surveillance of COVID-19 (EAVE II): protocol for an observational study using linked Scottish national data. BMJ Open.

[20] National Services Scotland (2021). National data catalogue: rapid preliminary inpatient data (RAPID). https://www.ndc.scot.nhs.uk/National-Datasets/data.asp?SubID=37.

[21] National Services Scotland (2021). Turas vaccine management tool. https://learn.nes.nhs.scot/42708/turas-vaccination-management-tool.

[22] von Elm E, Altman DG, Egger M, Pocock SJ, Gøtzsche PC, Vandenbroucke JP (2007). The strengthening the reporting of observational studies in epidemiology (STROBE) statement: guidelines for reporting observational studies. Lancet.

[23] Benchimol EI, Smeeth L, Guttmann A (2015). The reporting of studies conducted using observational routinely-collected health data (RECORD) statement. PLoS Med.

[24] EAVE II analysts (2021). Analysis plan to investigate potential waning of COVID-19 vaccine protection after the completion of a primary immunisation schedule. https://www.ed.ac.uk/files/atoms/files/sap_vaccine_waning_2nddose_10sep2021.pdf.

[25] Centers for Disease Control and Prevention (2021). Interim clinical considerations for use of COVID-19 vaccines currently approved or authorized in the United States. https://www.cdc.gov/vaccines/covid-19/clinical-considerations/covid-19-vaccines-us.html.

[26] Folegatti PM, Ewer KJ, Aley PK (2020). Safety and immunogenicity of the ChAdOx1 nCoV-19 vaccine against SARS-CoV-2: a preliminary report of a phase 1/2, single-blind, randomised controlled trial. Lancet.

[27] Clift AK, Coupland CAC, Keogh RH (2020). Living risk prediction algorithm (QCOVID) for risk of hospital admission and mortality from coronavirus 19 in adults: national derivation and validation cohort study. BMJ.

[28] US Food and Drug Administration (2020). Development and licensure of vaccines to prevent COVID-19. https://www.fda.gov/regulatory-information/search-fda-guidance-documents/development-and-licensure-vaccines-prevent-covid-19.

[29] Hodgson SH, Mansatta K, Mallett G, Harris V, Emary KRW, Pollard AJ (2021). What defines an efficacious COVID-19 vaccine? A review of the challenges assessing the clinical efficacy of vaccines against SARS-CoV-2. Lancet Infect Dis.

[30] Polack FP, Thomas SJ, Kitchin N (2020). Safety and efficacy of the BNT162b2 mRNA COVID-19 vaccine. N Engl J Med.

[31] Tartof SY, Slezak JM, Fischer H (2021). Effectiveness of mRNA BNT162b2 COVID-19 vaccine up to 6 months in a large integrated health system in the USA: a retrospective cohort study. Lancet.

[32] Goldberg Y, Mandel M, Bar-On YM (2021). Waning immunity of the BNT162b2 vaccine in Israel. N Engl J Med.

[33] Pouwels KB, Pritchard E, Matthews PC (2021). Effect of delta variant on viral burden and vaccine effectiveness against new SARS-CoV-2 infections in the UK. Nature.

[34] Shrotri M, Navaratnam AMD, Nguyen V (2021). Spike-antibody waning after second dose of BNT162b2 or ChAdOx1. Lancet.

[35] Andrews N, Tessier E, Stowe J (2021). Vaccine effectiveness and duration of protection of Comirnaty, Vaxzevria and Spikevax against mild and severe COVID-19 in the UK. medRxiv.

[36] Lopez Bernal J, Andrews N, Gower C (2021). Effectiveness of COVID-19 vaccines against the B.1.617.2 (delta) variant. N Engl J Med.

[37] Hacisuleyman E, Hale C, Saito Y (2021). Vaccine breakthrough infections with SARS-CoV-2 variants. N Engl J Med.

[38] Planas D, Veyer D, Baidaliuk A (2021). Reduced sensitivity of SARS-CoV-2 variant delta to antibody neutralization. Nature.

[39] Voysey M, Costa Clemens SA, Madhi SA (2021). Single-dose administration and the influence of the timing of the booster dose on immunogenicity and efficacy of ChAdOx1 nCoV-19 (AZD1222) vaccine: a pooled analysis of four randomised trials. Lancet.

[40] Sheikh A, Robertson C, Taylor B (2021). BNT162b2 and ChAdOx1 nCoV-19 vaccine effectiveness against death from the delta variant. N Engl J Med.

[41] Rothman KJ (1990). No adjustments are needed for multiple comparisons. Epidemiology.

[42] Barros-Martins J, Hammerschmidt SI, Cossmann A (2021). Immune responses against SARS-CoV-2 variants after heterologous and homologous ChAdOx1 nCoV-19/BNT162b2 vaccination. Nat Med.

