# Supplementary material for: Two-dose ChAdOx1 nCoV-19 vaccine protection against COVID-19 hospital admissions and deaths over time: a retrospective, population-based cohort study in Scotland and Brazil
Source: Lancet. 2022 Jan 1;399(10319):25–35. doi: 10.1016/S0140-6736(21)02754-9 (PMC8687670; doi:10.1016/S0140-6736(21)02754-9)
Supplement: Portuguese translation of the abstract [file mmc1.pdf]

THE LANCET

## Supplementary appendix

This translation in Portuguese was submitted by the authors and we reproduce it as supplied. It has not been peer reviewed. The Lancet's editorial processes have only been applied to the original in English, which should serve as reference for this manuscript.

Esta tradução em português foi submetida pelos autores e nós não fizemos quaisquer alterações. Esta versão não foi revista por pares. O processo editorial do The Lancet só foi aplicado à versão original em inglês, que deve servir como referência para este artigo.

Supplement to: Katikireddi SV, Cerqueira-Silva T, Vasileiou E, et al. Two-dose ChAdOx1 nCoV-19 vaccine protection against COVID-19 hospital admissions and deaths over time: a retrospective, population-based cohort study in Scotland and Brazil. *Lancet* 2021; published online Dec 20. https://doi.org/10.1016/S0140-6736(21)02754-9.

**Resumo**

Situação
Várias publicações sugerem que a efetividade das vacinas (EV) contra COVID19 diminuem com o tempo após a vacinação, mas não está claro quanto disto reflete o declínio do efeito da vacina e quanto se deve à presença de novas variantes - especialmente a Delta. Nós investigamos a associação entre o tempo após a administração de duas doses da ChAdOx1 e o risco de desfechos graves da COVID-19 na Escócia (onde Delta era dominante), com análises comparativas no Brasil (no período que a Delta era incomum).

Métodos
Conectamos bancos de dados nacionais referentes a vacinação, testes laboratoriais, dados clínicos e de mortalidade. Definimos coortes de adultos que receberam duas doses de ChAdOx1 e comparamos as taxas de desfechos graves da COVID-19 (hospitalização ou morte por COVID-19) em períodos quinzenais, em relação a 2-3 semanas após a segunda dose. Os desfechos secundários foram infecção sintomática por SARS-CoV-2 confirmada laboratorialmente e desfechos separados de hospitalização ou morte por COVID-19. A regressão de Poisson foi utilizada para estimar as razões de taxas (RRs) e EV, com intervalos de confiança de 95% (IC95%).

Resultados
Dois milhões (m) de pessoas receberam ChAdOx1 na Escócia e 42.6m no Brasil, com acompanhamento mais longo na Escócia. Na Escócia, os RRs para COVID-19 grave aumentaram para 2,01 (IC 95% 1,54-2,62) em 10-11 semanas, 3,01 (IC 95% 2,26-3,99) em 14-15 semanas e) 5,43 (IC 95% 4,00-7,38 ) em 18-19 semanas. O padrão de resultados foi semelhante no Brasil, com RRs de 2,29 (IC95% 2,01-2,61), 3,10 (2,63-3,64) e 4,71 (3,83-5,78) para os mesmos períodos. Na Escócia, as EVs caíram de 83,7% (IC de 95% 79,7-87,0) em 2-3 semanas, para 75,9 (72,9-78,6) em 14-15 semanas e 53,6 (48,4-58,3) em 20-21 semanas. As EVs também diminuíram com o tempo no Brasil. Houve evidência de diminuição da proteção contra a infecção sintomática de SARS-CoV-2 ao longo do tempo em ambos os países.

Interpretação
Encontramos diminuição da proteção vacinal de ChAdOx1 contra infecção sintomática por SARS-CoV-2, hospitalizações por COVID-19 e mortes na Escócia e no Brasil, tornando-se evidente três meses após a segunda dose da vacina. Deve-se considerar o fornecimento de doses de vacina de reforço para pessoas que receberam ChAdOx1.

Financiamento
UK Research and Innovation (Medical Research Council), Governo Escocês, Research and Innovation Industrial Strategy Challenge Fund, Health Data Research UK, Fiocruz, Programa Fazer o Bem Faz Bem; Conselho Nacional de Desenvolvimento Científico e Tecnológico (CNPq), Fundação Carlos Chagas Filho de Amparo à Pesquisa do Estado do Rio de Janeiro (FAPERJ).
