# Supplementary material for: Two-dose ChAdOx1 nCoV-19 vaccine protection against COVID-19 hospital admissions and deaths over time: a retrospective, population-based cohort study in Scotland and Brazil
Source: Lancet. 2022 Jan 1;399(10319):25–35. doi: 10.1016/S0140-6736(21)02754-9 (PMC8687670; doi:10.1016/S0140-6736(21)02754-9)
Supplement: Supplementary appendix 2 [file mmc2.pdf]

THE LANCET

Supplementary appendix

This appendix formed part of the original submission and has been peer reviewed. We post it as supplied by the authors.

Supplement to: Katikireddi SV, Cerqueira-Silva T, Vasileiou E, et al. Two-dose ChAdOx1 nCoV-19 vaccine protection against COVID-19 hospital admissions and deaths over time: a retrospective, population-based cohort study in Scotland and Brazil. *Lancet* 2021; published online Dec 20. https://doi.org/10.1016/S0140-6736(21)02754-9.

Supplementary Appendix: Two-dose ChAdOx1 vaccine protection against SARS-CoV-2 symptomatic infections, and COVID-19 hospitalisations and deaths over time: Population-based cohort studies in Scotland and Brazil

Table S1: Joint Committee on Vaccination and Immunisation (JCVI) COVID-19 vaccination priority list, Scotland 3
Table S2: Brazilian Ministry of Health National Covid-19 Immunisation Plan 6
Table S3: Additional population characteristics for Scotland 8
Table S4: Additional population characteristics for Brazil 9
Table S5: QCovid risk groups and codes 10
Table S6: Study population for test-negative case-control design in Scotland 11
Table S7: Study population for test-negative design case-control study in Brazil 15
Table S8: Rate ratios for severe COVID-19 (i.e. COVID-19 hospitalisation or death) over time since completing two ChAdOx1 doses in Scotland for: a) Overall adult population; b) Stratified by age group 16
Table S9: Rate ratios for confirmed SARS-CoV-2 symptomatic infections over time since completing two doses in Scotland for: a) Overall adult population; b) Stratified by age group 18
Table S10: Trend test for: a) Overall adult population; b) Stratified by age group 20
Table S11: Rate ratios for COVID-19 hospitalisations over time since completing two ChAdOx1 doses in Scotland for the overall adult population 21
Table S12: Rate ratios for COVID-19 death over time since completing two ChAdOx1 doses in Scotland for the overall adult population 21
Table S13: Rate ratios for severe COVID-19 (hospitalisation/death) over time since completing two ChAdOx1 doses in Brazil for: a) Overall adult population; b) Stratified by age group 22
Table S14: Rate ratios for COVID-19 death over time since completing two ChAdOx1 doses in Brazil for the overall adult population 24
Table S15: Rate ratios for confirmed symptomatic SARS-CoV-2 infections over time since completing two ChAdOx1 doses in Brazil, overall and age-stratified 25
Table S17: Vaccine effectiveness estimates in Brazil, restricted to follow-up when presence of delta variant was minimal (sensitivity analysis) 27
Table S18: Rate ratios for Covid-19 hospitalisations over time since completing two ChAdOx1 doses in Brazil for: a) Overall adult population; b) Stratified by age group 28
Table S19: Rate ratios for COVID-19 deaths over time since completing two ChAdOx1 doses in Brazil for: a) Overall adult population; b) Stratified by age group 30
Table S20: Vaccine effectiveness estimates for ChAdOx1 against hospitalisations or mortality by length of time since two-dose vaccination in Scotland (reference group: unvaccinated), stratified by age group 32
Table S21: Rate ratios over time since receiving two ChAdOx1 doses for severe COVID-19 in Scotland using Pillar 2 testing (sensitivity analysis) 34
Table S22: Vaccine effectiveness estimates in Scotland for severe COVID-19 using Pillar 2 testing for outcome ascertainment in Scotland (sensitivity analysis) 34
Table S23: Rate ratios over time since receiving two ChAdOx1 doses for severe COVID-19 in Scotland among 40-64 year olds (sensitivity analysis) 35
Table S24: Vaccine effectiveness against severe COVID-19 estimated using an alternative reference period (0-13 days post-first ChAdOx1 dose) in Scotland 35

Table S25: Vaccine effectiveness estimates for ChAdOx1 against COVID-19 hospitalisations or death by length of time since full vaccination in Brazil (reference group: 0-13 days post-first dose vaccination), stratified by age 36
Table S25: Rate ratios over time since receiving two ChAdOx1 doses for severe COVID-19 in Scotland using an incidence-density matched case-control design (sensitivity analysis) 38
Table S26: Formulae for models 38
Table S26: Estimates of covariates from main adjusted models for the primary outcome (COVID-19 hospitalisation or death) in two-dose and vaccine effectiveness cohorts in Scotland 39
Table S29: ICD-10 codes for COVID-19 illness 45
Table S30: Reporting STROBE and RECORD checklists 46

Figure S1: Distribution of variants over time in Scotland 52
Figure S2: Data linkage diagram for Scotland 53
Figure S3: Data linkage diagram for Brazil 55
Figure S4: Distribution of variants over time in Brazil, stratified by region 57
Figure S5: ChAdOx1 two-dose vaccination numbers in Scotland, stratified by age group 58
Figure S6: STROBE Flow diagram for Scotland 59
Figure S7: Trends in COVID-related outcomes in Scotland for: a) COVID-19 hospitalisation or death; b) COVID-19 hospitalisation; c) COVID-19 death; d) SARS-CoV-2 confirmed infection 61
Figure S8: Association between waning and calendar period effects for vaccines aged 65+ in Scotland 63
Figure S9: STROBE Flow diagram for Brazil 65
Figure S10: Monthly numbers of: a) COVID-19 hospitalisation or death; b) COVID-19 hospitalisation; c) COVID-19 death; d) SARS-CoV-2 confirmed symptomatic infection in Brazil, stratified by age group 66
Figure S11: Cumulative uptake for receipt of any vaccination in Brazil, stratified by age group 68
Figure S12: ChAdOx1 vaccination numbers in Brazil, stratified by age group 68

Table S1: Joint Committee on Vaccination and Immunisation (JCVI) COVID-19 vaccination priority list, Scotland

Order of priority	Group*	Estimated population
**Phase 1 of the vaccination programme**		
1	Residents and workers in care homes for older people	30,000 residents;<br>45,000 staff
2	All those over 80 years of age and over	250,000
	Patient facing, frontline healthcare workers	230,000
	Non-clinical but patient facing staff in secondary or primary care/community healthcare settings	
	Laboratory and pathology staff	
	Social care staff directly involved in the care of their service users and others involved directly in delivering social care such that they and vulnerable patients/clients are at increased risk of exposure	
3	All those 75 years of age and over	190,000
4	All those 70 years of age and over and clinically extremely vulnerable individuals	280,000 over 70; 110,000 clinically extremely vulnerable
5	All those 65 years of age and over	280,000
6	All individuals aged 16 years to 64 years with underlying health conditions which put them at higher risk of serious disease and mortality and Unpaid carers	1,010,000
7	All those 60 years of age and over	280,000
8	All those 55 years of age and over	330,000
9	All those 50 years of age and over	340,000
**Phase 2 of the vaccination programme**		

1	All those aged 40 to 49 years
2	All those aged 30 to 39 years
3	All those 18 to 29 years

*These groups represent around 99% of preventable mortality from COVID-19

The vaccine roll-out strategy has been determined by an independent UK-wide body, namely the Joint Commission on Vaccinations and Immunisation (JCVI),[1] which has prioritised vaccinations to adults on the basis of assessing the risk of serious COVID-19 outcomes, in particular hospitalisations and deaths.[1] In Scotland, the vaccination programme is rolled out into two phases. Phase 1 included all adults over the age of 50, those with underlying health conditions and health or social care workers. Phase 2 included all adults under the age of 50 not already vaccinated as part of priority groups 1 to 9 in Phase1.[2,3] First and second doses of Pfizer-BioNTech, Oxford-AstraZeneca and Moderna vaccines are currently administered in Scotland. Pfizer-BioNTech and Oxford-AstraZeneca vaccines were approved by United Kingdom's (UK) Medicines and Healthcare products Regulatory Agency (MHRA) on 8 December 2020 and Moderna was approved on 8 January 2021.[1] On 7 May 2021, JCVI announced that all adults aged 18-39 years olds should not be offered the Oxford-AstraZeneca vaccine due to increased risk of blood clots and bleeding post-vaccination in younger adults.[4]

Individuals in these priority groups received a written invitation ~14 days before their appointment. They were asked however to delay their vaccination if they had recently had COVID-19, tested positive or were self-isolating. These invitations were accompanied by written advice on the need to observe behavioural measures to reduce the risk of contracting the infection.

Prior to vaccination, checks were made by the trained administering staff to see if individuals had COVID-19 or tested positive in the preceding 4 weeks; if so, the vaccination was deferred. Immediately following vaccination, individuals received both verbal and written advice on the need to maintain behavioural measures, particularly in the 2-3 weeks following vaccination.

Because of the different storage requirements for the two vaccines, GPs have administered the Oxford-AstraZeneca vaccine and vaccine centres have mainly administered the Pfizer-BioNTech vaccine. Guided by JCVI priorities, GPs began by focusing their efforts on: a) the mobile elderly who they vaccinated in their general practice surgeries; and b) care home residents affiliated with general practices. Vaccination centres began with focusing on health and social care providers before extending to other JCVI priority groups. By February 22nd 2021, Group 7 vaccination was underway and a full roll-out of invitations to Group 6 started.

References:

[1] Joint Committee on Vaccination and Immunisation. Priority groups for coronavirus (COVID-19) vaccination: advice from the JCVI, 30 December 2020. Available from: https://www.gov.uk/government/publications/priority-groups-for-coronavirus-covid-19-vaccination-advice-from-the-jcvi-30-december-2020
[2] Scottish Government. Coronavirus (COVID-19): vaccine deployment plan 2021. Available from: https://www.gov.scot/publications/coronavirus-covid-19-vaccine-deployment-plan-2021/

[3] Scottish Government. . Coronavirus (COVID-19): vaccine deployment plan: update – July 2021. Available from: https://www.gov.scot/publications/coronavirus-covid-19-vaccine-deployment-plan-update---july-2021/documents/
[4] Public Health England. COVID-19 vaccination and blood clotting. Available at:
https://www.gov.uk/government/publications/covid-19-vaccination-and-blood-clotting/covid-19-vaccination-and-blood-clotting

Table S2: Brazilian Ministry of Health National Covid-19 Immunisation Plan

Order of Priority	Group	Estimated Population
1	Elderly people in nursing homes, aged 60 years and older	160,472
2	People with Disabilities in nursing homes	6,472
3	Indigenous Peoples Living in Indigenous Lands	413,739
4	Health Workers	7,337,807
5	People aged 90 years and older	893,873
6	People aged 85 to 89	1,299,948
7	People aged 80 to 84	2,247,225
8	People aged 75 to 79	3,614,384
9	Traditional Peoples and Communities Riverside	632,815
10	Traditional Quilombola Peoples and Communities	1,184,383
11	People aged 70 to 74	5,408,657
12	People aged 65 to 69	7,349,241
13	People aged 60 to 64	9,383,724
14	People with comorbidities and pregnant women and puerperal women with Comorbidities (n=18,218,730); Persons with Permanent Disabilities registered in federal social assistance (BPC) (n=1,467,477); Pregnant and Postpartum women (n=2,488,052)	22.174.259
15	Persons with Permanent Disabilities (18 to 59 years) not registered at BPC	6,281,581
16	Homeless people (18 to 59 years)	140,559
17	Employees of the Prisons (n=108,949) and Prisoners (n=753,966)	862,915
18	Primary Education Workers (daycare, preschools, elementary school, high school)	2,707,200
19	Higher Education Workers	719,818
20	Security and Rescue Forces (n=604,511), Army, Navy and Airforce (n=364,631)	969.142
21	Public Transport Workers	678,264
22	Rail and Metro Transport Workers	73,504
23	Air Transport Workers	165,944
24	Waterway Transport Workers	41,515
25	Truck Drivers	1,241,061
26	Port Workers	111,397
27	Industrial Workers	5,323,291
28	Urban cleaning and solid waste management workers	228,218
29	General Population - People aged 18 to 59	76,443,686
Total		158,095,094

The vaccine roll-out strategy for Brazil has been determined by the Ministry of Health – Health Surveillance Secretariat – National Immunisation Programme, advised by a commission with representatives of the states, municipalities, health professional associations and the Pan American Health Organisation – World Health Organisation (PAHO – WHO). The reference document for the vaccine roll-out strategy (National Covid-19 immunisation plan) has been updated on monthly basis and is available on the following link: https://www.gov.br/saude/pt-br/coronavirus/publicacoes-tecnicas/guias-e-planos/plano-nacional-de-vacinacao-covid-19/view

The plan defines guidelines for vaccines administration and determines the delivery of doses for the 5,570 municipalities and the federal district, which administered the doses to their resident population. Immunisation pace varied in each municipality due to factors such as vaccine availability, local health facilities capabilities, and resident priority groups adherence.

Each municipality had its own communication strategy to call the population to receive the doses. In most of the cases there was no individual written invitation. Usually posting the schedule in the Municipal Health Secretariat Website and local media notices sufficed for people to show on the indicated health facilities. Larger cities often launched websites and mobile phone apps for scheduling the doses.

The population was not routinely tested for previous or active COVID-19 infections, but if they had any COVID-19 related symptoms they were instructed to postpone vaccination for 4 weeks. Different vaccine types were not targeted at different

population subgroups, except for pregnant women who were not usually given ChAdOx1. However, for all other patient groups, the specific vaccine type administered depended on availability, with patients not offered a choice over their vaccine type.

Table S3: Additional population characteristics for Scotland

Characteristic	Level	Two-dose cohort	VE cohort
Number of previous tests *	0	1,374,415 (69.7%)	2,081,024 (82.1%)
	1	354,730 (18.0%)	315,770 (12.5%)
	2	123,457 (6.3%)	73,019 (2.9%)
	3-4	70,912(3.6%)	33,310 (1.3%)
	5-9	30,724(1.6%)	16,753 (0.7%)
	10+	18,216 (0.9%)	14,652 (0.6%)
Number of risk groups †	0	994,558 (50.4%)	1,363,199 (53.8%)
	1	567,960 (28.8%)	698,667 (27.6%)
	2	241,737 (12.3%)	281,194 (11.1%)
	3	99,184 (5.1%)	112,488 (4.4%)
	4	42,029 (2.1%)	47,647 (1.9%)
	≥5	26,986 (1.4%)	31,333 (1.2%)
Urban/Rural index	1 Large Urban Areas	595,827 (30.2%)	852,840 (33.6%)
	2 Other Urban Areas	749,514 (38.0%)	926,846 (36.6%)
	3 Accessible Small Towns	200,861 (10.2%)	240,976 (9.5%)
	4 Remote Small Towns	108,106 (5.5%)	129,347 (5.1%)
	5 Accessible Rural	200,579(10.2%)	239,566 (9.5%)
	6 Remote Rural	107,402 (5.4%)	126,368 (5.0%)
	Unknown	10,165 (0.5%)	18,584 (0.7%)

* Number of previous tests: Proxy for working in a high-risk occupation (e.g. healthcare worker)
† Number of risk groups: Individual QCovid risk groups found in Table S13

Table S4: Additional population characteristics for Brazil

Characteristic	Level	Two dose cohort	VE cohort
Region	Northeast	10,105,954 (23.7%)	14,254,472 (25.4%)
	North	3,046,408 (7.2%)	4,587,710 (8.2%)
	Southeast	18,666,201 (43.9%)	23,968,607 (42.8%)
	South	7,300,485 (17.2%)	8,764,490 (15.6%)
	Central West	3,223,931 (7.6%)	4,132,622 (7.4%)
	Unknown	215,860 (0.5%)	305,737 (0.5%)
Vaccination category	High risk age group	24,069,720 (56.6%)	34,008,604 (60.7%)
	Comorbidities/Pregnant/Puerperal period	8,627,958 (20.3%)	9,663,617 (17.3%)
	General workforce	3,632,578 (8.5%)	4,319,301 (7.7%)
	Heath Care Workers	4,559,818 (10.7%)	5,533,679 (9.9%)
	Indigenous or Traditional community	628,170 (1.5%)	800,217 (1.4%)
	Other/Missing	704,499 (1.7%)	1,206,068 (2.2%)
	Vulnerable Population	336,048 (0.8%)	482,004 (0.9%)

Table S5: QCovid risk groups and codes

QCovid risk group	Code
Atrial fibrillation	Q_DIAG_AF
Asthma	Q_DIAG_ASTHMA
Blood cancer	Q_DIAG_BLOOD_CANCER
Heart failure	Q_DIAG_CCF
Cerebral palsy	Q_DIAG_CEREBALPALSY
Coronary heart disease	Q_DIAG_CHD
Cirrhosis	Q_DIAG_CIRRHOSIS
Congenital heart disease	Q_DIAG_CONGEN_HD
COPD	Q_DIAG_COPD
Dementia	Q_DIAG_DEMENTIA
Diabetes type 1	Q_DIAG_DIABETES_1
Diabetes type 2	Q_DIAG_DIABETES_2
Epilepsy	Q_DIAG_EPILEPSY
Fracture	Q_DIAG_FRACTURE
Neurological disorder	Q_DIAG_NEURO
Parkinson’s	Q_DIAG_PARKINSONS
Pulmonary hypertension	Q_DIAG_PULM_HYPER
Pulmonary rare	Q_DIAG_PULM_RARE
Peripheral vascular disease	Q_DIAG_PVD
Rheumatoid arthritis or SLE	Q_DIAG_RA_SLE
Respiratory cancer	Q_DIAG_RESP_CANCER
Severe mental illness	Q_DIAG_MENT_ILL
Sickle cell disease	Q_DIAG_SICKLE_CELL
Stroke/TIA	Q_DIAG_STROKE
Thrombosis or pulmonary embolus	Q_DIAG_VTE
Care housing category	Q_HOME_CAT
Learning disability or Down's	Q_LEARN_CAT
Kidney disease	Q_DIAG_CKD_LEVEL

More information on codes: https://github.com/EAVE-II/EAVE-II-data-dictionary
Ref: Clift, A.K., et al. Living risk prediction algorithm (QCovid) for risk of hospital admission and mortality from coronavirus 19 in adults: national derivation and validation cohort study. BMJ 371, m3731 (2020).

Table S6: Study population for test-negative case-control design in Scotland

Characteristic	Level	Negative tests (%)	Positive tests (%)	Unvaccinated (%)	Vaccinated (%)	ChAdOx1
Age	Mean (SD)	41.3 (15.9)	40.9 (16.2)	26.8 (7.7)	42.2 (16.0)	50.0 (13.8)
Sex	Female	126994 (56.5)	73962 (50.4)	11936 (45.7)	188930 (54.7)	86723 (53.7)
	Male	97747 (43.5)	72693 (49.6)	14194 (54.3)	156162 (45.3)	74838 (46.3)
Deprivation	1 - High	42697 (19.6)	30474 (21.4)	5293 (21.4)	67850 (20.2)	32570 (20.6)
	2	43150 (19.8)	29449 (20.7)	4965 (20.1)	67599 (20.1)	32426 (20.5)
	3	40436 (18.5)	25610 (18.0)	4408 (17.8)	61604 (18.3)	29105 (18.4)
	4	43572 (20.0)	27128 (19.0)	4597 (18.6)	66071 (19.7)	30662 (19.4)
	5-Low	46922 (21.5)	28996 (20.3)	5216 (21.1)	70659 (21.0)	32515 (20.6)
	Missing	1364 (0.6)	881 (0.6)	232 (0.9)	2011 (0.6)	788 (0.5)
Smoking status	Non-smoker	104748 (49.0)	70452 (50.4)	11938 (49.3)	163166 (49.6)	74611 (48.1)
	Ex-smoker	26003 (12.2)	16966 (12.1)	1378 (5.7)	41565 (12.6)	24710 (15.9)
	Smoker	47367 (22.2)	27890 (19.9)	2964 (12.3)	72260 (21.9)	41627 (26.8)
	Unknown	35677 (16.7)	24538 (17.5)	7914 (32.7)	52284 (15.9)	14272 (9.2)
Blood pressure	No Investigation	50601 (23.7)	34581 (24.7)	10990 (45.4)	74158 (22.5)	20177 (13.0)
	Low	3847 (1.8)	2475 (1.8)	522 (2.2)	5799 (1.8)	2381 (1.5)
	Normal	137966 (64.5)	88764 (63.5)	11843 (49.0)	214767 (65.2)	110031 (70.9)
	High	17903 (8.4)	11758 (8.4)	740 (3.1)	28907 (8.8)	18751 (12.1)
	Very High	3478 (1.6)	2268 (1.6)	99 (0.4)	5644 (1.7)	3880 (2.5)
QRisk AF	0	211732 (99.0)	138386 (99.0)	24185 (100.0)	325761 (98.9)	152739 (98.4)
	1	2063 (1.0)	1460 (1.0)	9 (0.0)	3514 (1.1)	2481 (1.6)
QRisk Asthma	0	177966 (83.2)	118103 (84.5)	20713 (85.6)	275206 (83.6)	128671 (82.9)
	1	35829 (16.8)	21743 (15.5)	3481 (14.4)	54069 (16.4)	26549 (17.1)
QRisk blood cancer	0	213085 (99.7)	139436 (99.7)	24191 (100.0)	328158 (99.7)	154442 (99.5)

	1	710 (0.3)	410 (0.3)	3 (0.0)	1117 (0.3)	778 (0.5)
QRisk CCF	0	212904 (99.6)	139209 (99.5)	24190 (100.0)	327751 (99.5)	154081 (99.3)
	1	891 (0.4)	637 (0.5)	4 (0.0)	1524 (0.5)	1139 (0.7)
QRisk cerebral palsy	0	213584 (99.9)	139720 (99.9)	24188 (100.0)	328944 (99.9)	154964 (99.8)
	1	211 (0.1)	126 (0.1)	6 (0.0)	331 (0.1)	256 (0.2)
QRisk CHD	0	209018 (97.8)	136630 (97.7)	24187 (100.0)	321289 (97.6)	149626 (96.4)
	1	4777 (2.2)	3216 (2.3)	7 (0.0)	7986 (2.4)	5594 (3.6)
QRisk cirrhosis	0	213012 (99.6)	139299 (99.6)	24183 (100.0)	327956 (99.6)	154334 (99.4)
	1	783 (0.4)	547 (0.4)	11 (0.0)	1319 (0.4)	886 (0.6)
QRisk congenital heart disease	0	212173 (99.2)	138756 (99.2)	24166 (99.9)	326591 (99.2)	153268 (98.7)
	1	1622 (0.8)	1090 (0.8)	28 (0.1)	2684 (0.8)	1952 (1.3)
QRisk COPD	0	210012 (98.2)	137920 (98.6)	24182 (100.0)	323578 (98.3)	151077 (97.3)
	1	3783 (1.8)	1926 (1.4)	12 (0.0)	5697 (1.7)	4143 (2.7)
QRisk dementia	0	213606 (99.9)	139708 (99.9)	24194 (100.0)	328948 (99.9)	154973 (99.8)
	1	189 (0.1)	138 (0.1)	0 (0.0)	327 (0.1)	247 (0.2)
QRisk type 1 diabetes	0	212625 (99.5)	139016 (99.4)	24184 (100.0)	327285 (99.4)	153684 (99.0)
	1	1170 (0.5)	830 (0.6)	10 (0.0)	1990 (0.6)	1536 (1.0)
QRisk type 2 diabetes	0	206492 (96.6)	134401 (96.1)	24150 (99.8)	316571 (96.1)	146157 (94.2)
	1	7303 (3.4)	5445 (3.9)	44 (0.2)	12704 (3.9)	9063 (5.8)
QRisk epilepsy	0	211085 (98.7)	138151 (98.8)	24130 (99.7)	324934 (98.7)	151752 (97.8)
	1	2710 (1.3)	1695 (1.2)	64 (0.3)	4341 (1.3)	3468 (2.2)
QRisk fracture	0	205561 (96.1)	134403 (96.1)	23338 (96.5)	316456 (96.1)	148988 (96.0)
	1	8234 (3.9)	5443 (3.9)	856 (3.5)	12819 (3.9)	6232 (4.0)
QRisk neurological condition	0	213113 (99.7)	139415 (99.7)	24191 (100.0)	328165 (99.7)	154379 (99.5)
	1	682 (0.3)	431 (0.3)	3 (0.0)	1110 (0.3)	841 (0.5)

QRisk Parkinsons	0	213645 (99.9)	139772 (99.9)	24193 (100.0)	329052 (99.9)	155046 (99.9)
	1	150 (0.1)	74 (0.1)	1 (0.0)	223 (0.1)	174 (0.1)
QRisk pulmonary hypertension	0	213641 (99.9)	139736 (99.9)	24194 (100.0)	329011 (99.9)	155012 (99.9)
	1	154 (0.1)	110 (0.1)	0 (0.0)	264 (0.1)	208 (0.1)
QRisk rare pulmonary disease	0	213039 (99.6)	139403 (99.7)	24189 (100.0)	328081 (99.6)	154352 (99.4)
	1	756 (0.4)	443 (0.3)	5 (0.0)	1194 (0.4)	868 (0.6)
QRisk PVD	0	212998 (99.6)	139355 (99.6)	24185 (100.0)	327996 (99.6)	154328 (99.4)
	1	797 (0.4)	491 (0.4)	9 (0.0)	1279 (0.4)	892 (0.6)
QRisk rheumatoid conditions	0	211878 (99.1)	138649 (99.1)	24176 (99.9)	326179 (99.1)	152978 (98.6)
	1	1917 (0.9)	1197 (0.9)	18 (0.1)	3096 (0.9)	2242 (1.4)
QRisk Respiartory cancer	0	213590 (99.9)	139701 (99.9)	24193 (100.0)	328926 (99.9)	154957 (99.8)
	1	205 (0.1)	145 (0.1)	1 (0.0)	349 (0.1)	263 (0.2)
QRisk Severe Mental illness	0	185874 (86.9)	124625 (89.1)	22750 (94.0)	287602 (87.3)	130149 (83.8)
	1	27921 (13.1)	15221 (10.9)	1444 (6.0)	41673 (12.7)	25071 (16.2)
QRisk Sickle Cell	0	213638 (99.9)	139763 (99.9)	24193 (100.0)	329036 (99.9)	155057 (99.9)
	1	157 (0.1)	83 (0.1)	1 (0.0)	239 (0.1)	163 (0.1)
QRisk Stroke	0	211334 (98.8)	138191 (98.8)	24178 (99.9)	325175 (98.8)	152334 (98.1)
	1	2461 (1.2)	1655 (1.2)	16 (0.1)	4100 (1.2)	2886 (1.9)
QRisk VTE	0	211740 (99.0)	138407 (99.0)	24172 (99.9)	325803 (98.9)	152582 (98.3)
	1	2055 (1.0)	1439 (1.0)	22 (0.1)	3472 (1.1)	2638 (1.7)
QRisk care home	0	213568 (99.9)	139719 (99.9)	24172 (99.9)	328943 (99.9)	155001 (99.9)
	1	99 (0.0)	56 (0.0)	5 (0.0)	150 (0.0)	111 (0.1)
	2	128 (0.1)	71 (0.1)	17 (0.1)	182 (0.1)	108 (0.1)
QRisk Learning difficulties	0	210870 (98.6)	138287 (98.9)	23885 (98.7)	325101 (98.7)	152866 (98.5)
	1	2868 (1.3)	1543 (1.1)	309 (1.3)	4101 (1.2)	2298 (1.5)

	2	57 (0.0)	16 (0.0)	0 (0.0)	73 (0.0)	56 (0.0)
QRisk CKD	Mean (SD)	0.0 (0.4)	0.0 (0.4)	0.0 (0.0)	0.0 (0.4)	0.1 (0.5)
QRisk no. of risk groups	0	132028 (61.8)	89184 (63.8)	18534 (76.6)	202551 (61.5)	83199 (53.6)
	1	59275 (27.7)	37396 (26.7)	4995 (20.6)	91636 (27.8)	48434 (31.2)
	2	16267 (7.6)	9513 (6.8)	606 (2.5)	25169 (7.6)	16410 (10.6)
	3	4238 (2.0)	2495 (1.8)	53 (0.2)	6680 (2.0)	4761 (3.1)
	4	1330 (0.6)	814 (0.6)	4 (0.0)	2140 (0.6)	1572 (1.0)
	5+	657 (0.3)	444 (0.3)	2 (0.0)	1099 (0.3)	844 (0.5)
BMI	Mean (SD)	27.5 (4.4)	27.5 (4.3)	26.0 (3.1)	27.6 (4.4)	28.4 (4.7)
Health board	NHS AYRSHIRE & ARRAN	14860 (6.6)	9939 (6.8)	1661 (6.4)	23138 (6.7)	10654 (6.6)
	NHS BORDERS	3504 (1.6)	1980 (1.4)	354 (1.4)	5130 (1.5)	2437 (1.5)
	NHS DUMFRIES & GALLOWAY	4526 (2.0)	2769 (1.9)	288 (1.1)	7007 (2.0)	3722 (2.3)
	NHS FIFE	14413 (6.4)	9567 (6.5)	1517 (5.8)	22463 (6.5)	11009 (6.8)
	NHS FORTH VALLEY	12603 (5.6)	8035 (5.5)	1117 (4.3)	19521 (5.7)	9924 (6.1)
	NHS GRAMPIAN	18885 (8.4)	9952 (6.8)	1576 (6.0)	27243 (7.9)	12018 (7.4)
	NHS GREATER GLASGOW & CLYDE	56335 (25.1)	38310 (26.1)	6908 (26.5)	87636 (25.4)	37114 (23.0)
	NHS HIGHLAND	10727 (4.8)	6065 (4.1)	1171 (4.5)	15621 (4.5)	7156 (4.4)
	NHS LANARKSHIRE	32416 (14.4)	22529 (15.4)	2501 (9.6)	52443 (15.2)	28016 (17.3)
	NHS LOTHIAN	41113 (18.3)	26309 (17.9)	6544 (25.1)	60824 (17.6)	28377 (17.6)
	NHS ORKNEY	244 (0.1)	98 (0.1)	4 (0.0)	338 (0.1)	128 (0.1)
	NHS SHETLAND	198 (0.1)	20 (0.0)	4 (0.0)	214 (0.1)	10835 (6.7)
	NHS TAYSIDE	14764 (6.6)	10986 (7.5)	2461 (9.4)	23289 (6.8)	96 (0.1)
	NHS WESTERN ISLES	54 (0.0)	34 (0.0)	7 (0.0)	81 (0.0)	20 (0.0)
In QRisk Learning difficulties condition category 2 refers to Down’s syndrome; In QRisk Care home condition, category 2 refers to homeless						

Table S7: Study population for test-negative design case-control study in Brazil

Patient characteristic		Negative tests (%)	Positive tests (%)	Unvaccinated (%)	Vaccinated (%)	ChAdOx1
Age	Mean (SD)	37 (16.3)	40 (17.0)	36 (14.8)	46 (20)	49 (16.3)
Sex	Female	4,250,817 (55.6%)	3,291,203 (51.9%)	5,070,090 (51.5%)	2,471,930 (59.7%)	1,133,682 (59.0%)
	Male	3,399,407 (44.4%)	3,050,015 (48.1%)	4,781,963 (48.5%)	1,667,459 (40.3%)	788,401 (41.0%)
Deprivation	1 - High	2,527,084 (33.0%)	1,723,330 (27.2%)	2,876,866 (29.2%)	1,373,548 (33.2%)	640,017 (33.3%)
	2	1,700,287 (22.2%)	1,335,704 (21.1%)	2,116,290 (21.5%)	919,701 (22.2%)	400,943 (20.9%)
	3	1,384,119 (18.1%)	1,356,698 (21.4%)	1,985,735 (20.2%)	755,082 (18.2%)	354,339 (18.4%)
	4	1,321,997 (17.3%)	1,211,384 (19.1%)	1,828,837 (18.6%)	704,544 (17.0%)	339,974 (17.7%)
	5-Low	712,310 (9.3%)	698,669 (11.0%)	1,031,381 (10.5%)	379,598 (9.2%)	183,723 (9.6%)
	Missing	4,427 (0.1%)	15,433 (0.2%)	12,944 (0.1%)	6,916 (0.2%)	3,087 (0.2%)
Race	White	3,297,385 (43.1%)	2,637,330 (41.6%)	4,071,164 (41.3%)	1,863,551 (45.0%)	866,076 (45.1%)
	Black	295,935 (3.9%)	239,579 (3.8%)	388,846 (3.9%)	146,668 (3.5%)	67,278 (3.5%)
	Asian	92,686 (1.2%)	82,222 (1.3%)	127,079 (1.3%)	47,829 (1.2%)	20,830 (1.1%)
	Mixed Race	2,139,858 (28.0%)	1,969,576 (31.1%)	2,994,519 (30.4%)	1,114,915 (26.9%)	520,838 (27.1%)
	Indigenous	8,610 (0.1%)	4,827 (0.1%)	4,673 (0.0%)	8,764 (0.2%)	571 (0.0%)
	Missing	1,815,750 (23.7%)	1,407,684 (22.2%)	2,265,772 (23.0%)	957,662 (23.1%)	446,490 (23.2%)
Diabetes Mellitus	Present	212,075 (2.8%)	302,729 (4.8%)	268,729 (2.7%)	246,075 (5.9%)	119,201 (6.2%)
Obesity	Present	73,562 (1.0%)	134,155 (2.1%)	138,651 (1.4%)	69,066 (1.7%)	35,222 (1.8%)
Immunosuppression	Present	43,928 (0.6%)	40,561 (0.6%)	49,957 (0.5%)	34,532 (0.8%)	17,790 (0.9%)
Cardiac disease	Present	390,228 (5.1%)	495,979 (7.8%)	485,663 (4.9%)	400,544 (9.7%)	189,360 (9.9%)
Puerperal period (up to 45 days after delivery)	Present	4,237 (0.1%)	3,624 (0.1%)	6,187 (0.1%)	1,674 (0.0%)	306 (0.0%)
Pregnancy	Present	60,785 (0.8%)	31,179 (0.5%)	71,561 (0.7%)	20,403 (0.5%)	2,419 (0.1%)
Chronic kidney disease	Present	22,637 (0.3%)	32,166 (0.5%)	29,698 (0.3%)	25,105 (0.6%)	10,971 (0.6%)

Table S8: Rate ratios for severe COVID-19 (i.e. COVID-19 hospitalisation or death) over time since completing two ChAdOx1 doses in Scotland for: a) Overall adult population; b) Stratified by age group

Follow-up period (weeks post second dose)	Person-years	Number of events	RR (95% CI)
**Overall**			
0 to 1	44,978	78	1.24 (0.91-1.69)
2 to 3	52,718	84	Ref
4 to 5	63,161	100	0.87 (0.65-1.16)
6 to 7	68,874	134	1.02 (0.77-1.35)
8 to 9	71,541	245	1.81 (1.40-2.35)
10 to 11	71,093	280	2.01 (1.54-2.62)
12 to 13	68,520	336	2.52 (1.92-3.32)
14 to 15	64,495	353	3.01 (2.26-3.99)
16 to 17	60,849	397	4.01 (2.99-5.38)
18 to 19	51,211	497	5.43 (4.00-7.38)
20 to 21	37,526	567	7.39 (5.37-10.18)
**18-64 years old**			
0 to 1	43,935	73	1.17 (0.84-1.64)
2 to 3	47,879	69	Ref
4 to 5	49,813	75	0.94 (0.67-1.31)
6 to 7	50,130	84	1.04 (0.75-1.46)
8 to 9	49,969	130	1.94 (1.40-2.69)
10 to 11	49,275	136	2.40 (1.71-3.37)
12 to 13	46,714	151	2.74 (1.93-3.91)
14 to 15	42,721	196	3.75 (2.61-5.38)
16 to 17	39,148	212	5.18 (3.57-7.52)
18 to 19	29,636	201	7.51 (5.10-11.04)
20 to 21	16,176	158	11.11 (7.41-16.65)
**65-79 years old**			
0 to 1	935	†	0.59 (0.17-2.08)
2 to 3	4,484	13	Ref
4 to 5	11,061	15	0.44 (0.20-0.93)
6 to 7	13,522	32	0.52 (0.26-1.05)
8 to 9	14,113	76	0.78 (0.40-1.52)
10 to 11	14,142	79	0.63 (0.31-1.24)
12 to 13	14,119	91	0.84 (0.42-1.69)
14 to 15	14,088	83	0.93 (0.45-1.93)
16 to 17	14,028	105	0.88 (0.41-1.87)
18 to 19	13,924	194	1.07 (0.50-2.31)
20 to 21	13,724	210	1.13 (0.51-2.49)

80+ years old			
0 to 1	109	†	1.84 (0.26-13.12)
2 to 3	354	†	Ref
4 to 5	2,287	10	1.65 (0.36-7.61)
6 to 7	5,221	18	1.12 (0.26-4.88)
8 to 9	7,459	39	1.10 (0.26-4.61)
10 to 11	7,676	65	1.02 (0.24-4.24)
12 to 13	7,687	94	1.09 (0.26-4.54)
14 to 15	7,686	74	0.87 (0.21-3.64)
16 to 17	7,674	80	1.16 (0.27-4.93)
18 to 19	7,651	102	1.19 (0.28-5.14)
20 to 21	7,627	199	1.53 (0.35-6.63)

*Adjusted for age, sex, deprivation, comorbidities, number of previous tests, interval between doses and temporal trend.
†Values suppressed due to small numbers

Table S9: Rate ratios for confirmed SARS-CoV-2 symptomatic infections over time since completing two doses in Scotland for: a) Overall adult population; b) Stratified by age group

Follow-up period (weeks post second dose)	Person-years	Number of events	RR (95% CI)
**Overall**			
0 to 1	47,415	2,034	1.36 (1.28-1.44)
2 to 3	55,478	2,350	Ref
4 to 5	65,955	3,294	0.97 (0.92-1.03)
6 to 7	71,388	4,146	1.06 (1.01-1.12)
8 to 9	73,762	4,472	1.09 (1.04-1.15)
10 to 11	73,204	6,136	1.20 (1.14-1.27)
12 to 13	71,396	7,527	1.24 (1.17-1.31)
14 to 15	67,446	7,074	1.22 (1.16-1.30)
16 to 17	63,336	6,044	1.24 (1.17-1.32)
18 to 19	57,492	5,520	1.34 (1.26-1.43)
20 to 21	44,615	3,860	1.39 (1.29-1.48)
**18-64 years old**			
0 to 1	46,241	2,030	1.35 (1.27-1.44)
2 to 3	49,927	2,333	Ref
4 to 5	51,654	3,255	0.99 (0.94-1.05)
6 to 7	51,743	4,048	1.10 (1.05-1.17)
8 to 9	51,394	4,256	1.16 (1.10-1.23)
10 to 11	50,650	5,791	1.27 (1.20-1.34)
12 to 13	48,875	7,176	1.31 (1.24-1.39)
14 to 15	44,976	6,691	1.31 (1.24-1.39)
16 to 17	40,948	5,409	1.36 (1.27-1.44)
18 to 19	35,263	4,488	1.46 (1.37-1.56)
20 to 21	22,631	2,781	1.46 (1.36-1.57)
**65-79 years old**			
0 to 1	46,241	2,030	1.35 (1.27-1.44)
2 to 3	49,927	2,333	Ref
4 to 5	51,654	3,255	0.99 (0.94-1.05)
6 to 7	51,743	4,048	1.10 (1.05-1.17)
8 to 9	51,394	4,256	1.16 (1.10-1.23)
10 to 11	50,650	5,791	1.27 (1.20-1.34)
12 to 13	48,875	7,176	1.31 (1.24-1.39)
14 to 15	44,976	6,691	1.31 (1.24-1.39)
16 to 17	40,948	5,409	1.36 (1.27-1.44)
18 to 19	35,263	4,488	1.46 (1.37-1.56)
20 to 21	22,631	2,781	1.46 (1.36-1.57)
**80+ years old**			

0 to 1	1,057	†	0.75 (0.25-2.28)
2 to 3	5,124	15	Ref
4 to 5	11,761	34	0.85 (0.46-1.59)
6 to 7	14,057	90	1.11 (0.61-2.00)
8 to 9	14,617	196	1.17 (0.65-2.10)
10 to 11	14,627	288	1.33 (0.74-2.38)
12 to 13	14,588	272	1.52 (0.84-2.75)
14 to 15	14,544	309	1.88 (1.03-3.42)
16 to 17	14,476	566	2.05 (1.12-3.75)
18 to 19	14,341	891	2.30 (1.25-4.24)
20 to 21	14,135	791	2.42 (1.31-4.50)

*Adjusted for age, sex, deprivation, comorbidities, number of previous tests, interval between doses and temporal trend.
†Values suppressed due to small numbers

Table S10: Trend test for: a) Overall adult population; b) Stratified by age group

Follow-up period (weeks post second dose)	Scotland	Brazil
	P-value	
**Overall**		
3-4 week +	<0.00001	<0.00001
5-6 week +	<0.00001	<0.00001
7-8 week +	<0.00001	<0.00001
**18-64 years old**		
3-4 week +	<0.00001	<0.00001
5-6 week +	<0.00001	<0.00001
7-8 week +	<0.00001	<0.00001
**65-79 years old**		
3-4 week +	0.0051	<0.00001
5-6 week +	0.0113	<0.00001
7-8 week +	0.0265	<0.00001
**80+ years old**		
3-4 week +	0.5523	<0.00001
5-6 week +	0.2362	<0.00001
7-8 week +	0.1559	<0.00001

All analyses from fully adjusted models.

Table S11: Rate ratios for COVID-19 hospitalisations over time since completing two ChAdOx1 doses in Scotland for the overall adult population

Follow-up period (weeks post second dose)	Person-years	Number of events	RR* (95% CI)
0 to 1	44,978	78	1.23 (0.90-1.68)
2 to 3	52,718	84	Ref
4 to 5	63,161	99	0.87 (0.65-1.17)
6 to 7	68,874	131	1.02 (0.77-1.35)
8 to 9	71,541	243	1.88 (1.45-2.43)
10 to 11	71,093	276	2.11 (1.61-2.75)
12 to 13	68,520	315	2.55 (1.93-3.36)
14 to 15	64,495	345	3.20 (2.40-4.26)
16 to 17	60,849	370	4.12 (3.05-5.55)
18 to 19	51,211	464	5.65 (4.14-7.72)
20 to 21	37,526	521	7.72 (5.58-10.69)

* Adjusted for age, sex, deprivation, comorbidities, number of previous tests, interval between doses and temporal trend.
† Values suppressed due to small numbers

Table S12: Rate ratios for COVID-19 death over time since completing two ChAdOx1 doses in Scotland for the overall adult population

Follow-up period (weeks post second dose)	Person-years	Number of events	RR* (95% CI)
0 to 1	59,377	†	0.70 (0.14-3.62)
2 to 3	67,290	5	Ref
4 to 5	71,449	6	0.79 (0.24-2.59)
6 to 7	72,343	10	0.98 (0.33-2.91)
8 to 9	72,136	18	1.37 (0.48-3.86)
10 to 11	71,683	38	2.26 (0.83-6.20)
12 to 13	70,212	64	3.58 (1.30-9.83)
14 to 15	66,543	54	3.27 (1.15-9.27)
16 to 17	62,692	71	5.00 (1.73-14.48)
18 to 19	57,078	82	5.88 (1.97-17.52)
20 to 21	44,270	134	9.32 (3.05-28.47)

* Adjusted for age, sex, deprivation, comorbidities, number of previous tests, interval between doses and temporal trend.
† Values suppressed due to small numbers

Table S13: Rate ratios for severe COVID-19 (hospitalisation/death) over time since completing two ChAdOx1 doses in Brazil for: a) Overall adult population; b) Stratified by age group

Follow-up period (weeks post second dose)	Person-years	Number of events	RR (95% CI)
**Overall**			
0 to 1	1,565,461	2,531	1.85 (1.71-2.00)
2 to 3	1,473,040	1,083	Ref
4 to 5	1,321,841	1,013	1.23 (1.12-1.35)
6 to 7	1,103,439	1,018	1.64 (1.48-1.82)
8 to 9	851,489	861	1.80 (1.60-2.03)
10 to 11	642,304	750	2.29 (2.01-2.61)
12 to 13	439,999	644	2.95 (2.56-3.41)
14 to 15	261,089	470	3.10 (2.63-3.64)
16 to 17	167,191	395	3.90 (3.26-4.67)
18 to 19	130,428	274	4.71 (3.83-5.78)
**18-64 years**			
0 to 1	1,383,537	1,271	1.99 (1.77-2.23)
2 to 3	1,287,689	454	Ref
4 to 5	1,137,024	448	1.40 (1.22-1.61)
6 to 7	919,949	427	1.95 (1.67-2.27)
8 to 9	670,186	277	2.04 (1.70-2.46)
10 to 11	464,809	156	2.26 (1.80-2.83)
12 to 13	272,090	85	2.92 (2.21-3.86)
14 to 15	130,572	37	2.71 (1.87-3.92)
16 to 17	70,249	27	3.32 (2.18-5.05)
18 to 19	51,942	26	4.78 (3.11-7.35)
**65-79 years**			
0 to 1	126,333	488	1.62 (1.37-1.92)
2 to 3	127,521	249	Ref
4 to 5	126,911	217	1.10 (0.91-1.34)
6 to 7	125,724	228	1.49 (1.20-1.84)
8 to 9	123,769	222	1.82 (1.45-2.29)
10 to 11	120,278	218	2.63 (2.06-3.36)
12 to 13	111,174	189	3.61 (2.77-4.71)
14 to 15	74,528	105	3.47 (2.54-4.74)
16 to 17	41,800	82	4.90 (3.44-6.97)
18 to 19	25,300	49	5.26 (3.45-8.01)
**80+ years**			
0 to 1	55,591	772	1.72 (1.48-2.00)
2 to 3	57,829	380	Ref
4 to 5	57,906	348	1.11 (0.94-1.32)
6 to 7	57,766	363	1.37 (1.12-1.68)
8 to 9	57,534	362	1.49 (1.18-1.87)
10 to 11	57,217	376	1.82 (1.41-2.34)
12 to 13	56,735	370	2.18 (1.66-2.87)

14 to 15	55,989	328	2.46 (1.82-3.32)
16 to 17	55,142	286	2.90 (2.08-4.04)
18 to 19	53,185	199	3.44 (2.37-4.99)

* Adjusted for sex, age in 5 years band up to 80, deprivation status, macro-region of residence, calendar week, interval between doses and primary reason for vaccination.

Table S14: Rate ratios for COVID-19 death over time since completing two ChAdOx1 doses in Brazil for the overall adult population

Follow-up period (weeks post second dose)	Person-years	Number of events	RR (95% CI)
0 to 1	1,565,034	878	1.74 (1.52-1.99)
2 to 3	1,472,503	388	Ref
4 to 5	1,320,697	356	1.21 (1.04-1.42)
6 to 7	1,102,505	369	1.63 (1.37-1.95)
8 to 9	851,876	312	1.77 (1.44-2.17)
10 to 11	642,456	288	2.32 (1.85-2.91)
12 to 13	438,577	246	2.80 (2.18-3.60)
14 to 15	260,208	186	2.95 (2.21-3.94)
16 to 17	166,882	132	3.59 (2.58-5.00)
18 to 19	130,548	83	4.80 (3.25-7.08)

Table S15: Rate ratios for confirmed symptomatic SARS-CoV-2 infections over time since completing two ChAdOx1 doses in Brazil, overall and age-stratified

Follow-up period (weeks post second dose)	Person-years	Number of events	RR* (95% CI)
**Overall**			
0 to 1	1,629,924	28,781	1.31 (1.29-1.34)
2 to 3	1,525,929	17,926	Ref
4 to 5	1,350,928	15,497	1.13 (1.11-1.16)
6 to 7	1,106,456	12,279	1.29 (1.25-1.32)
8 to 9	851,751	9,337	1.45 (1.41-1.50)
10 to 11	642,038	7,070	1.66 (1.60-1.72)
12 to 13	438,953	5,124	1.87 (1.80-1.94)
14 to 15	260,605	3,354	1.86 (1.78-1.94)
16 to 17	166,992	2,536	2.04 (1.95-2.14)
18 to 19	130,039	1,851	2.18 (2.06-2.30)
**18-64 years**			
0 to 1	1,448,103	23,898	1.30 (1.27-1.33)
2 to 3	1,340,491	14,996	Ref
4 to 5	1,166,182	12,906	1.14 (1.11-1.17)
6 to 7	923,192	9,805	1.28 (1.24-1.31)
8 to 9	670,641	7,009	1.45 (1.41-1.50)
10 to 11	464,887	4,807	1.62 (1.56-1.69)
12 to 13	271,635	3,199	1.78 (1.70-1.86)
14 to 15	130,971	1,977	1.74 (1.65-1.83)
16 to 17	70,705	1,438	1.86 (1.76-1.97)
18 to 19	52,068	1,202	2.11 (1.98-2.25)
**65-79 years**			
0 to 1	121,768	2,502	1.33 (1.24-1.43)
2 to 3	122,884	1,526	Ref
4 to 5	122,131	1,348	1.13 (1.05-1.23)
6 to 7	120,835	1,301	1.43 (1.31-1.57)
8 to 9	118,906	1,216	1.67 (1.51-1.84)
10 to 11	115,368	1,086	2.09 (1.87-2.33)
12 to 13	106,133	855	2.58 (2.29-2.91)
14 to 15	69,336	507	2.79 (2.43-3.21)
16 to 17	37,063	326	3.42 (2.91-4.03)
18 to 19	20,977	151	3.08 (2.49-3.81)
**80+ years**			
0 to 1	55,626	2,227	1.40 (1.28-1.52)
2 to 3	58,027	1,318	Ref
4 to 5	58,097	1,151	1.10 (1.00-1.21)
6 to 7	57,932	1,102	1.35 (1.20-1.51)
8 to 9	57,757	1,018	1.44 (1.27-1.65)
10 to 11	57,397	1,066	1.80 (1.56-2.08)
12 to 13	56,881	970	2.08 (1.77-2.45)

14 to 15	56,131	803	2.23 (1.87-2.67)
16 to 17	55,212	730	2.73 (2.24-3.32)
18 to 19	53,245	473	2.82 (2.25-3.53)

* Adjusted for sex, age in 5 years band up to 80, deprivation status, macro-region of residence, calendar week, interval between doses and primary reason for vaccination.

Table S16: Vaccine effectiveness estimates in Brazil, restricted to follow-up when presence of delta variant was minimal (sensitivity analysis)

Follow-up period (weeks post second dose)	ChAdOx1		
	Person-years	Number of Events	VE* (95% CI)
0 to 2 post-first dose	1,749,528	23,845	Ref
Partially vaccinated†	6,153,141	31,797	59.5 (58.6-60.3)
0 to 1	384,334	1,379	71.9 (70.2-73.6)
2 to 3	224,864	511	84.5 (83-85.9)
4 to 5	153,068	400	83.1 (81.2-84.7)
6 to 7	122,680	358	76.4 (73.6-78.9)
8 to 9	88,473	197	70.8 (66.2-74.8)
10 to 11	55,337	95	67.5 (60-73.5)
12 to 13	30,550	28	61.5 (44-73.5)

* Adjusted for sex, age in 5 years band up to 80, deprivation status, macro-region of residence, calendar week, interval between doses and primary reason for vaccination.
† Partially vaccinated: ≥2 weeks post-first dose and before second dose

Table S17: Rate ratios for Covid-19 hospitalisations over time since completing two ChAdOx1 doses in Brazil for: a) Overall adult population; b) Stratified by age group

Follow-up period (weeks post second dose)	Person-years	Number of events	RR* (95% CI)
**Overall**			
0 to 1	1,585,306	2491	1.86 (1.72-2.02)
2 to 3	1,491,714	1062	Ref
4 to 5	1,338,600	997	1.23 (1.12-1.36)
6 to 7	1,117,429	1010	1.66 (1.50-1.84)
8 to 9	862,285	850	1.81 (1.61-2.04)
10 to 11	650,448	745	2.31 (2.02-2.63)
12 to 13	445,578	641	2.97 (2.57-3.43)
14 to 15	264,399	468	3.12 (2.65-3.67)
16 to 17	169,311	392	3.92 (3.27-4.70)
18 to 19	132,081	271	4.71 (3.83-5.78)
**18-64 years**			
0 to 1	1,401,081	1258	1.98 (1.76-2.23)
2 to 3	1,304,018	450	Ref
4 to 5	1,151,443	444	1.40 (1.22-1.61)
6 to 7	931,615	427	1.96 (1.68-2.29)
8 to 9	678,685	276	2.05 (1.70-2.47)
10 to 11	470,703	156	2.27 (1.81-2.86)
12 to 13	275,541	84	2.92 (2.21-3.86)
14 to 15	132,228	36	2.67 (1.84-3.89)
16 to 17	71,140	27	3.36 (2.21-5.12)
18 to 19	52,601	26	4.86 (3.16-7.48)
**65-79 years**			
0 to 1	127,933	480	1.64 (1.39-1.95)
2 to 3	129,137	245	Ref
4 to 5	128,519	213	1.09 (0.90-1.33)
6 to 7	127,318	227	1.48 (1.20-1.83)
8 to 9	125,338	219	1.79 (1.42-2.25)
10 to 11	121,803	216	2.59 (2.03-3.32)
12 to 13	112,584	189	3.57 (2.73-4.65)
14 to 15	75,473	105	3.41 (2.49-4.67)
16 to 17	42,330	82	4.79 (3.36-6.82)
18 to 19	25,621	48	4.98 (3.26-7.61)
**80+ years**			
0 to 1	56,292	753	1.75 (1.50-2.04)
2 to 3	58,559	367	Ref
4 to 5	58,638	340	1.13 (0.95-1.34)
6 to 7	58,496	356	1.41 (1.14-1.73)
8 to 9	58,262	355	1.53 (1.21-1.93)
10 to 11	57,941	373	1.87 (1.45-2.41)
12 to 13	57,453	368	2.23 (1.69-2.95)

14 to 15	56,698	327	2.53 (1.86-3.42)
16 to 17	55,840	283	2.94 (2.10-4.11)
18 to 19	53,860	197	3.49 (2.40-5.08)

* Adjusted for sex, age in 5 years band up to 80, deprivation status, macro-region of residence, calendar week, interval between doses and primary reason for vaccination.

Table S18: Rate ratios for COVID-19 deaths over time since completing two ChAdOx1 doses in Brazil for: a) Overall adult population; b) Stratified by age group

Follow-up period (weeks post second dose)	Person-years	Number of events	RR (95% CI)
**Overall**			
0 to 1	1,565,034	878	1.74 (1.52-1.99)
2 to 3	1,472,503	388	Ref
4 to 5	1,320,697	356	1.21 (1.04-1.42)
6 to 7	1,102,505	369	1.63 (1.37-1.95)
8 to 9	851,876	312	1.77 (1.44-2.17)
10 to 11	642,456	288	2.32 (1.85-2.91)
12 to 13	438,577	246	2.80 (2.18-3.60)
14 to 15	260,208	186	2.95 (2.21-3.94)
16 to 17	166,882	132	3.59 (2.58-5.00)
18 to 19	130,548	83	4.80 (3.25-7.08)
**18-64 years**			
0 to 1	1,384,870	277	1.74 (1.36-2.23)
2 to 3	1,288,906	104	Ref
4 to 5	1,137,739	103	1.52 (1.13-2.04)
6 to 7	920,949	94	2.16 (1.54-3.03)
8 to 9	672,471	51	2.12 (1.39-3.26)
10 to 11	466,852	30	2.51 (1.48-4.25)
12 to 13	272,507	13	2.67 (1.34-5.33)
14 to 15	131,000	6	3.14 (1.27-7.75)
16 to 17	70,789	3	2.96 (0.88-10.02)
18 to 19	52,201	1	1.74 (0.23-13.07)
**65-79 years**			
0 to 1	124,364	184	1.37 (1.05-1.79)
2 to 3	125,577	102	Ref
4 to 5	124,835	86	1.03 (0.76-1.40)
6 to 7	123,613	81	1.23 (0.88-1.73)
8 to 9	121,701	87	1.87 (1.31-2.68)
10 to 11	118,267	78	2.58 (1.75-3.82)
12 to 13	109,247	62	3.47 (2.25-5.35)
14 to 15	73,131	37	3.47 (2.08-5.80)
16 to 17	40,830	24	4.50 (2.46-8.22)
18 to 19	25,095	9	3.57 (1.57-8.13)
**80+ years**			
0 to 1	55,801	417	1.98 (1.62-2.42)
2 to 3	58,021	182	Ref
4 to 5	58,123	167	1.13 (0.89-1.43)
6 to 7	57,943	194	1.53 (1.17-2.00)
8 to 9	57,703	174	1.57 (1.15-2.13)
10 to 11	57,337	180	2.06 (1.46-2.90)
12 to 13	56,823	171	2.43 (1.66-3.54)

14 to 15	56,078	143	2.70 (1.77-4.11)
16 to 17	55,263	105	3.32 (2.07-5.33)
18 to 19	53,253	73	5.30 (3.12-9.02)

* Adjusted for sex, age in 5 years band up to 80, deprivation status, macro-region of residence, calendar week, interval between doses and primary reason for vaccination.
†Values suppressed due to small numbers.

Table S19: Vaccine effectiveness estimates for ChAdOx1 against hospitalisations or mortality by length of time since two-dose vaccination in Scotland (reference group: unvaccinated), stratified by age group

Follow-up period (weeks post second dose)	Person-years	Number of events	VE* (95% CI)
**Overall**			
Unvaccinated	336,942	2,245	0.0 (0.0-0.0)
0 to 1 post-first dose	6,860	39	-15.4 (-60.6-17.0)
Partially vaccinated†	94,761	420	49.3 (43.3-54.6)
0 to 1	47,252	78	77.7 (71.9-82.3)
2 to 3	55,318	85	83.7 (79.7-87.0)
4 to 5	65,698	106	86.6 (83.6-89.0)
6 to 7	71,120	134	86.8 (84.2-88.9)
8 to 9	73,540	245	79.0 (75.9-81.7)
10 to 11	73,212	280	79.6 (76.8-82.1)
12 to 13	71,773	337	77.4 (74.6-80.0)
14 to 15	68,114	356	75.9 (72.9-78.6)
16 to 17	63,974	402	70.5 (67.0-73.7)
18 to 19	58,608	508	63.7 (59.6-67.4)
20 to 21	45,716	598	53.6 (48.4-58.3)
**18-64 years old**			
Unvaccinated	318,254	1,886	0.0 (0.0-0.0)
0 to 1 post-first dose	6,752	39	-14.8 (-60.3-17.8)
Partially vaccinated†	90,633	328	57.0 (51.2-62.1)
0 to 1	46,147	73	80.8 (75.5-84.9)
2 to 3	49,899	69	87.0 (83.4-89.9)
4 to 5	51,801	78	89.5 (86.8-91.7)
6 to 7	52,099	84	90.5 (88.0-92.4)
8 to 9	51,963	130	84.6 (81.4-87.2)
10 to 11	51,475	136	83.3 (80.0-86.1)
12 to 13	50,082	152	83.2 (80.0-85.9)
14 to 15	46,488	199	79.4 (75.9-82.4)
16 to 17	42,421	214	74.6 (70.4-78.3)
18 to 19	37,181	211	65.9 (60.0-70.9)
20 to 21	24,478	185	51.1 (41.9-58.9)
**65-79 years old**			
Unvaccinated	13,933	266	0.0 (0.0-0.0)
0 to 1 post-first dose	97	-	-
Partially vaccinated†	2,880	54	34.9 (12.5-51.6)
0 to 1	1,000	3	72.8 (13.9-91.4)
2 to 3	4,994	14	58.8 (24.7-77.5)
4 to 5	11,418	16	81.8 (68.0-89.6)
6 to 7	13,637	32	78.1 (66.7-85.6)
8 to 9	14,160	76	64.9 (52.4-74.1)
10 to 11	14,175	79	71.6 (62.0-78.8)
12 to 13	14,141	91	64.3 (53.0-72.8)

14 to 15	14,102	83	64.1 (52.8-72.7)
16 to 17	14,055	107	67.5 (58.4-74.6)
18 to 19	13,966	195	64.9 (56.8-71.4)
20 to 21	13,816	213	65.5 (57.9-71.8)
**80+ year olds**			
Unvaccinated	4,754	93	0.0 (0.0-0.0)
0 to 1 post-first dose	11	-	-
Partially vaccinated†	1,248	38	-26.5 (-85.1-13.5)
0 to 1	105	2	-55.3 (-537.4-62.1)
2 to 3	424	2	33.3 (-179.7-84.1)
4 to 5	2,479	12	-7.6 (-123.8-48.3)
6 to 7	5,384	18	33.5 (-22.5-64.0)
8 to 9	7,417	39	30.2 (-11.2-56.2)
10 to 11	7,562	65	35.0 (4.1-55.9)
12 to 13	7,550	94	30.9 (1.9-51.3)
14 to 15	7,524	74	47.2 (24.7-62.9)
16 to 17	7,498	81	32.1 (5.3-51.3)
18 to 19	7,461	102	36.9 (14.5-53.4)
20 to 21	7,421	200	20.6 (-3.8-39.2)

* Adjusted for age, sex, deprivation, comorbidities, number of previous tests, interval between doses and temporal trend
† Partially vaccinated: ≥2 weeks post-first dose and before second dose

Table S20: Rate ratios over time since receiving two ChAdOx1 doses for severe COVID-19 in Scotland using Pillar 2 testing (sensitivity analysis)

Follow-up period (weeks post second dose)	Person-years	Number of events	RR* (95% CI)
0 to 1	26,711	‡	0.90 (0.20 4.06)
2 to 3	31,851	‡	Ref
4 to 5	38,717	‡	0.47 (0.09 2.57)
6 to 7	41,110	5	1.06 (0.28 4.01)
8 to 9	40,429	13	2.09 (0.66 6.61)
10 to 11	37,172	16	2.25 (0.72 7.02)
12 to 13	34,950	12	2.07 (0.64 6.70)
14 to 15	32,778	16	3.33 (1.07 10.36)
16 to 17	30,150	12	2.38 (0.74 7.71)
18 to 19	29,004	24	4.13 (1.36 12.57)
20 to 21	28,695	45	8.19 (2.76 24.30)

* Adjusted for age, sex, deprivation, comorbidities, number of previous tests, interval between doses and temporal trend
‡ Values suppressed due to small numbers.

Table S21: Vaccine effectiveness estimates in Scotland for severe COVID-19 using Pillar 2 testing for outcome ascertainment in Scotland (sensitivity analysis)

Follow-up period (weeks post second dose)	Person-years	Number of events	RR* (95% CI)
Unvaccinated	336,942	887	Ref
0 to 1 post-first dose	6,860	11	28.1 (-32.4-60.9)
Partially vaccinated†	94,761	165	51.7 (42.1-59.6)
0 to 1	47,252	17	89.1 (82.3-93.3)
2 to 3	55,318	22	89.9 (84.4-93.4)
4 to 5	65,698	32	89.9 (85.5-92.9)
6 to 7	71,120	42	89.3 (85.3-92.2)
8 to 9	73,540	58	85.8 (81.3-89.2)
10 to 11	73,212	71	83.5 (78.8-87.1)
12 to 13	71,773	108	76.9 (71.5-81.3)
14 to 15	68,114	126	73.4 (67.5-78.2)
16 to 17	63,974	120	71.7 (65.2-77.0)
18 to 19	58,608	156	58.4 (49.5-65.7)
20 to 21	45,716	164	43.2 (30.8-53.4)

* Adjusted for age, sex, deprivation, comorbidities, number of previous tests, interval between doses and temporal trend
† Partially vaccinated: ≥2 weeks post-first dose and before second dose
‡ Values suppressed due to small numbers.

Table S22: Rate ratios over time since receiving two ChAdOx1 doses for severe COVID-19 in Scotland among 40-64 year olds (sensitivity analysis)

Follow-up period (weeks post second dose)	Person-years	Number of events	RR (95% CI)
0 to 1	38,269	60	1.13 (0.78-1.64)
2 to 3	41,567	58	Ref
4 to 5	43,199	64	0.96 (0.67-1.37)
6 to 7	43,441	69	1.03 (0.71-1.49)
8 to 9	43,280	114	2.03 (1.43-2.89)
10 to 11	42,646	115	2.46 (1.70-3.55)
12 to 13	40,250	123	2.79 (1.90-4.10)
14 to 15	36,513	163	3.96 (2.68-5.86)
16 to 17	33,385	183	5.65 (3.77-8.45)
18 to 19	25,070	170	7.99 (5.26-12.14)
20 to 21	13,391	137	11.93 (7.69-18.51)

Table S23: Vaccine effectiveness against severe COVID-19 estimated using an alternative reference period (0-13 days post-first ChAdOx1 dose) in Scotland

Follow-up period (weeks post second dose)	Person-years	Number of events	VE fully adjusted	VE minimally adjusted
Unvaccinated				
0 to 1 post-first dose (Ref)	6,412	38	Ref	Ref
Partially vaccinated†	134,874	488	63.7 (49.1-74.1)	58.8 (42.2, 70.7)
0 to 1	52,485	87	83.3 (75.3-88.7)	81.9 (73.2, 87.8)
2 to 3	63,055	90	88.5 (83.1-92.2)	87.1 (80.9, 91.3)
4 to 5	69,170	137	86.3 (80.1-90.5)	83.8 (76.4, 88.8)
6 to 7	72,186	239	78.7 (69.5-85.1)	73.9 (62.5, 81.8)
8 to 9	72,098	273	79.1 (70.0-85.4)	74.2 (62.8, 82.1)
10 to 11	70,990	338	76.2 (65.9-83.4)	70.6 (57.6, 79.6)
12 to 13	67,708	349	74.8 (63.7-82.5)	67.7 (53.4, 77.7)
14 to 15	63,639	398	68.6 (54.7-78.2)	57.7 (38.7, 70.8)
16 to 17	59,225	496	62.0 (45.1-73.7)	47.0 (22.8, 63.5)
18 to 19	47,382	597	51.8 (30.1-66.8)	30.7 (-1.2, 52.5)
20 to 21	33,880	549	43.7 (17.9-61.4)	17.6 (-21.1, 44.0)

Minimally adjusted includes age, sex, deprivation plus time periods whereas fully adjusted also included QCovid risk groups, number of previous tests and urban-rural classification.

† Partially vaccinated: ≥2 weeks post-first dose and before second dose

Table S24: Vaccine effectiveness estimates for ChAdOx1 against COVID-19 hospitalisations or death by length of time since full vaccination in Brazil (reference group: 0-13 days post-first dose vaccination), stratified by age

Follow-up period (weeks post second dose)	Person-years	Number of events	VE* (95% CI)
**Overall**			
0 to 2 post-first dose	1,849,099	21736	Ref
Partially vaccinated	11,701,310	37802	57.9 (56.9-58.9)
0 to 1	1,601,585	2688	73.2 (71.9-74.5)
2 to 3	1,492,259	1095	86.4 (85.4-87.3)
4 to 5	1,338,063	1019	83.5 (82.3-84.7)
6 to 7	1,117,983	1019	77.9 (76.1-79.5)
8 to 9	862,976	863	75.6 (73.4-77.6)
10 to 11	651,213	751	69.3 (66.3-72.1)
12 to 13	445,924	646	60.8 (56.6-64.6)
14 to 15	264,128	472	59.7 (54.6-64.2)
16 to 17	169,692	397	50.5 (43.4-56.6)
18 to 19	132,459	275	42.2 (32.4-50.6)
**18-64 years**			
0 to 2 post-first dose	1,733,794	19575	Ref
Partially vaccinated	10,498,560	25556	59.9 (58.8-60.9)
0 to 1	1,412,977	1343	76.4 (74.8-77.9)
2 to 3	1,304,208	460	89.9 (88.8-90.8)
4 to 5	1,150,939	449	86.6 (85.1-87.9)
6 to 7	932,197	427	81 (78.7-83)
8 to 9	679,491	278	80.1 (77.1-82.7)
10 to 11	471,565	156	79.3 (75.2-82.8)
12 to 13	276,017	85	73.5 (66.5-79)
14 to 15	132,338	38	73.7 (63.6-81.1)
16 to 17	71,730	27	68.7 (54.1-78.7)
18 to 19	53,177	26	59 (39.5-72.3)
**65-79 years**			
0 to 2 post-first dose	110,032	2045	Ref
Partially vaccinated	835,662	6711	41.7 (37.2-45.9)
0 to 1	129,587	514	63.4 (58.6-67.7)
2 to 3	129,105	252	78.2 (74.5-81.4)
4 to 5	128,303	219	77.4 (73.2-81)
6 to 7	127,116	229	72.1 (66.8-76.6)
8 to 9	125,081	222	68.5 (62.2-73.8)
10 to 11	121,588	219	54.3 (44.8-62.2)
12 to 13	112,337	189	40.7 (27.2-51.7)
14 to 15	74,978	105	48.9 (34.1-60.3)
16 to 17	42,052	82	25.5 (0.8-44.1)
18 to 19	25,487	49	13.3 (-23.4-39.1)
**80+ years**			

0 to 2 post-first dose	5,273	116	Ref
Partially vaccinated	367,083	5,535	25.7 (10.5-38.4)
0 to 1	59,022	831	45.5(33.6-55.3)
2 to 3	58,946	383	66.1(57.9-72.7)
4 to 5	58,821	351	59.0(48.7-67.3)
6 to 7	58,670	363	45.7(31.8-56.8)
8 to 9	58,404	363	40.5(24.9-52.9)
10 to 11	58,061	376	28.4(9.3-43.5)
12 to 13	57,570	372	15.1(-8.0-33.2)
14 to 15	56,812	329	8.8(-17.2-29.0)
16 to 17	55,910	288	-3.4(-34.6-20.6)
18 to 19	53,794	200	-19.1 (-59.2-10.9)

* Adjusted for age, sex, deprivation, macro-region of residence, primary reason for vaccination, interval between doses, and temporal trend

† Partially vaccinated: ≥2 weeks post-first dose and before second dose

Table S25: Rate ratios over time since receiving two ChAdOx1 doses for severe COVID-19 in Scotland using an incidence-density matched case-control design (sensitivity analysis)

Follow-up period (weeks post second dose)	RR (95% CI)
0 to 1	1.28 (0.94, 1.75)
2 to 3	1.00 (Ref)
4 to 5	0.82 (0.6, 1.12)
6 to 7	1.04 (0.76, 1.43)
8 to 9	1.64 (1.2, 2.26)
10 to 11	1.83 (1.32, 2.55)
12 to 13	2.39 (1.71, 3.36)
14 to 15	2.60 (1.84, 3.69)
16 to 17	4.07 (2.84, 5.83)
18 to 19	5.18 (3.58, 7.5)
20 to 21	7.60 (5.19, 11.13)

10 controls per case sampled, with exact matching for outcome date, sex, age (individual years up to 80, two-year bands 80-90, and five-year bands above that) and local authority. Statistical adjustment as per cohort models.

Table S26: Formulae for models

Model	Scotland	Brazil
Rate Ratios	Event ~ Poisson(PY*rate),<br>PY is the person time exposure in the period<br><br>log(rate) = Intercept + calendar_period + post_dose2_period + vaccine_gap + age_group + gender + deprivation + clinical and testing characteristics (see table S26 – part 1)	β_0+β_1*age+β_2*sex+β_3*week+β_4*state-of-residence+β_5*reason-to-vaccination+β_6*deprivation-municipal+β_7*post-vaccination-period+β_8*age*week+β_9*week*state-of-residence+β_10*age*state-of-residence
Vaccine Effects	Event ~ Poisson(PY*rate),<br>PY is the person time exposure in the period<br><br>log(rate) = Intercept + calendar_period + vaccine status + age_group + gender + deprivation + clinical and testing characteristics (see table S26 – part 2)	
Test Negative Design	Event ~ Binomial (N, p)<br>N is the number of tests;<br><br>logit(p) ~ Intercept + s(age) + s(days) + deprivation + clinical risk groups + number of tests groups + geographical region	

Table S27: Estimates of covariates from main adjusted models for the primary outcome (COVID-19 hospitalisation or death) in two-dose and vaccine effectiveness cohorts in Scotland

Estimates for covariates from model for estimating rate ratios in the overall two-dose cohort in Scotland

Variable	Categories	RR	L95	U95
	(Intercept)	0.00	0.00	0.00
Period	19/05/2021	0.60	0.40	0.90
	26/05/2021	0.70	0.49	1.01
	02/06/2021	0.75	0.54	1.05
	09/06/2021	0.60	0.43	0.84
	16/06/2021	0.85	0.63	1.13
	23/06/2021	1.00		
	30/06/2021	1.52	1.19	1.94
	07/07/2021	1.65	1.30	2.09
	14/07/2021	0.95	0.74	1.23
	21/07/2021	0.91	0.70	1.18
	28/07/2021	0.47	0.35	0.63
	04/08/2021	0.49	0.37	0.66
	11/08/2021	0.39	0.29	0.52
	18/08/2021	0.64	0.49	0.84
	25/08/2021	1.03	0.80	1.33
	01/09/2021	1.21	0.94	1.57
	08/09/2021	1.15	0.89	1.49
	15/09/2021	0.80	0.61	1.06
	22/09/2021	0.61	0.46	0.81
	29/09/2021	0.52	0.39	0.69
	06/10/2021	0.45	0.33	0.60
	13/10/2021	0.42	0.30	0.57
Post Vaccine Period	0 to 1	1.24	0.91	1.69
	2 to 3	1.00		
	4 to 5	0.87	0.65	1.16
	6 to 7	1.02	0.77	1.35
	8 to 9	1.81	1.40	2.35
	10 to 11	2.01	1.54	2.62
	12 to 13	2.52	1.92	3.32
	14 to 15	3.01	2.26	3.99
	16 to 17	4.01	2.99	5.38
	18 to 19	5.43	4.00	7.38
	20 to 21	7.39	5.37	10.18
Vaccine Gap	<7 weeks	1.01	0.81	1.26
	7-8 weeks	0.90	0.81	1.00
	9-10 weeks	0.92	0.86	1.00
	11-12 weeks	1.00		

	13+ weeks	1.47	1.23	1.76
Age Group	16-19	0.79	0.40	1.53
	20-24	0.57	0.37	0.87
	25-29	0.90	0.67	1.22
	30-34	0.89	0.68	1.16
	35-39	1.12	0.90	1.40
	40-44	1.02	0.84	1.25
	45-49	1.06	0.89	1.27
	50-54	0.91	0.78	1.06
	55-59	0.99	0.86	1.14
	60-69	1.00		
	65-69	1.03	0.88	1.20
	70-74	1.23	1.07	1.42
	75-79	1.22	1.06	1.41
	80-84	1.36	1.17	1.58
	85-89	1.53	1.30	1.80
	90+	1.78	1.48	2.16
Gender	Female	1.00		
	Male	1.32	1.24	1.41
Deprivation	Q1	1.00		
Scottish Index of	Q2	0.83	0.76	0.90
Multiple	Q3	0.62	0.56	0.68
Deprivation	Q4	0.56	0.51	0.62
Quintiles	Q5	0.45	0.41	0.50
Urban Rural	1 Large Urban Areas	1.00		
Classification	2 Other Urban Areas	0.91	0.85	0.97
	3 Accessible Small Towns	0.72	0.64	0.81
	4 Remote Small Towns	0.45	0.38	0.54
	5 Accessible Rural	0.67	0.59	0.76
	6 Remote Rural	0.35	0.28	0.43
Number of previous	0	1.00		
Covid Tests	1	1.53	1.41	1.66
	2	1.91	1.71	2.14
	3-4	2.68	2.40	3.00
	5-9	3.04	2.66	3.48
	10+	3.11	2.62	3.69
Body Mass Index	<20	1.00		
	20-24	0.98	0.79	1.21
	25-29	1.08	0.88	1.32
	30-34	1.24	1.01	1.53
	34-39	1.68	1.35	2.10
	40+	2.23	1.77	2.81
Health conditions	AF	1.07	0.96	1.19
	ASTHMA	1.20	1.11	1.30

	CCF	1.20	1.06	1.36
	CHD	1.26	1.17	1.37
	COPD	1.55	1.41	1.70
	DIABETES	1.75	1.62	1.88
	EPILEPSY	1.34	1.13	1.60
	FRACTURE	1.18	1.06	1.31
	PVD	1.00	0.86	1.17
	RA/SLE	1.54	1.30	1.81
	Severe Mental Illness	1.20	1.11	1.30
	STROKE	1.28	1.16	1.41
	VTE	1.36	1.20	1.53
	CKD	1.38	1.27	1.50
GP Smoking Status	Non-Smoker	1.00		
	Ex-Smoker	1.04	0.96	1.13
	Smoker	1.01	0.93	1.09
	Unknown	0.98	0.81	1.19
Blood Pressure	No Measurement	1.00		
	Low	1.50	1.10	2.04
	Normal	1.22	1.01	1.47
	High	1.28	1.05	1.57
	Very High	1.21	0.95	1.54
Number of other Q Covid risk groups	0	1.00		
	1	1.49	1.37	1.61
	2+	1.30	1.04	1.62

Estimates for covariates from model for estimating vaccine effectiveness in the overall population in Scotland

Variable	Category	RR	L95	U95
	(Intercept)	0.00	0.00	0.00
Period	18/05/2021	0.38	0.30	0.48
	25/05/2021	0.50	0.40	0.63
	01/06/2021	0.43	0.34	0.55
	08/06/2021	0.68	0.55	0.84
	15/06/2021	0.69	0.56	0.86
	22/06/2021	1.00		
	29/06/2021	1.61	1.36	1.92
	06/07/2021	2.08	1.76	2.46
	13/07/2021	1.59	1.33	1.90
	20/07/2021	1.25	1.03	1.50
	27/07/2021	0.91	0.75	1.11
	03/08/2021	0.87	0.71	1.06
	10/08/2021	0.68	0.55	0.84
	17/08/2021	1.02	0.85	1.24
	24/08/2021	1.83	1.54	2.17
	31/08/2021	2.53	2.15	2.98
	07/09/2021	2.60	2.21	3.07
	14/09/2021	2.11	1.79	2.49
	21/09/2021	1.52	1.28	1.81
	28/09/2021	1.36	1.14	1.62
	05/10/2021	1.21	1.01	1.46
	12/10/2021	1.20	1.00	1.44
	19/10/2021	0.79	0.65	0.96
Vaccine Status	Unvaccinated	1.00		
	0 to 1 post dose 1	1.15	0.83	1.61
	2+ dose 1	0.51	0.45	0.57
	0 to 1	0.22	0.18	0.28
	2 to 3	0.16	0.13	0.20
	4 to 5	0.13	0.11	0.16
	6 to 7	0.13	0.11	0.16
	8 to 9	0.21	0.18	0.24
	10 to 11	0.20	0.18	0.23
	12 to 13	0.23	0.20	0.25
	14 to 15	0.24	0.21	0.27
	16 to 17	0.29	0.26	0.33
	18 to 19	0.36	0.33	0.40
	20 to 21	0.46	0.42	0.52
Age Group	16-19	0.58	0.43	0.79
	20-24	0.42	0.35	0.50
	25-29	0.55	0.47	0.64

	30-34	0.68	0.59	0.79
	35-39	0.84	0.74	0.96
	40-44	0.78	0.69	0.88
	45-49	0.84	0.74	0.95
	50-54	0.87	0.77	0.98
	55-59	0.91	0.81	1.01
	60-69	1.00		
	65-69	1.12	0.99	1.26
	70-74	1.24	1.10	1.39
	75-79	1.34	1.20	1.51
	80-84	1.47	1.30	1.65
	85-89	1.73	1.52	1.97
	90+	1.88	1.61	2.20
Gender	Female	1.00		
	Male	1.18	1.13	1.24
Deprivation	Q1	1.00		
Scottish Index of	Q2	0.84	0.79	0.90
Multiple	Q3	0.69	0.64	0.74
Deprivation	Q4	0.62	0.58	0.67
Quintiles	Q5	0.52	0.48	0.56
Urban Rural	1 Large Urban Areas	1.00		
Classification	2 Other Urban Areas	0.95	0.90	1.00
	3 Accessible Small Towns	0.75	0.68	0.82
	4 Remote Small Towns	0.51	0.44	0.58
	5 Accessible Rural	0.72	0.65	0.79
	6 Remote Rural	0.42	0.36	0.50
Number of previous	0	1.00		
Covid Tests	1	1.62	1.52	1.72
	2	1.90	1.71	2.11
	3-4	2.48	2.20	2.80
	5-9	2.25	1.89	2.67
	10+	2.02	1.66	2.46
Body Mass Index	<20	1.00		
	20-24	1.07	0.91	1.27
	25-29	1.18	1.00	1.38
	30-34	1.50	1.28	1.77
	34-39	2.01	1.69	2.39
	40+	2.43	2.02	2.91
	AF	1.11	1.01	1.22
Health condition	ASTHMA	1.24	1.17	1.32
	CCF	1.19	1.06	1.34
	CHD	1.23	1.14	1.32
	COPD	1.66	1.53	1.80
	DIABETES	1.65	1.55	1.76

	EPILEPSY	1.31	1.13	1.51
	FRACTURE	1.20	1.10	1.31
	PVD	1.12	0.98	1.28
	RA/SLE	1.46	1.26	1.69
	Severe Mental Illness	1.15	1.08	1.23
	STROKE	1.26	1.15	1.37
	VTE	1.25	1.13	1.39
	CKD	1.32	1.23	1.43
GP Smoking Status	Non-Smoker	1.00		
	Ex-Smoker	0.99	0.93	1.06
	Smoker	0.87	0.82	0.92
	Unknown	1.08	0.98	1.20
Blood Pressure	No Measurement	1.00		
	Low	1.46	1.18	1.81
	Normal	1.62	1.47	1.79
	High	1.69	1.50	1.89
	Very High	1.68	1.44	1.97
Number of other	0	1.00		
Q Covid Risk Groups	1	1.54	1.43	1.65
	2+	1.44	1.18	1.74

See Table S5 for definition of QCovid groups

Table S28: ICD-10 codes for COVID-19 illness

Code	Description
U07.1	Covid-19, virus identified
U07.2	Covid-19, virus not identified
Source: https://www.who.int/classifications/icd/COVID-19-coding-icd10.pdf	

Table S29: Reporting STROBE and RECORD checklists

	Item No.	STROBE items	RECORD items	Location in manuscript where items are reported
**Title and abstract**				
	1	(a) Indicate the study's design with a commonly used term in the title or the abstract (b) Provide in the abstract an informative and balanced summary of what was done and what was found	RECORD 1.1: The type of data used should be specified in the title or abstract. When possible, the name of the databases used should be included.<br><br>RECORD 1.2: If applicable, the geographic region and timeframe within which the study took place should be reported in the title or abstract.<br><br>RECORD 1.3: If linkage between databases was conducted for the study, this should be clearly stated in the title or abstract.	Abstract
Background rationale	2	Explain the scientific background and rationale for the investigation being reported		Introduction, paras 1 and 2.
Objectives	3	State specific objectives, including any prespecified hypotheses		Introduction, last paragraph
Study Design	4	Present key elements of study design early in the paper		Methods, first paragraph
Setting	5	Describe the setting, locations, and relevant dates, including periods of recruitment, exposure, follow-up, and data collection		Methods – study design and datasets and comparative analyses sections
Participants	6	*(a) Cohort study* - Give the eligibility criteria, and the	RECORD 6.1: The methods of study population selection (such	Methods – study design

		sources and methods of selection of participants. Describe methods of follow-up<br>*Case-control study* - Give the eligibility criteria, and the sources and methods of case ascertainment and control selection. Give the rationale for the choice of cases and controls<br>*Cross-sectional study* - Give the eligibility criteria, and the sources and methods of selection of participants<br><br>*(b) Cohort study* - For matched studies, give matching criteria and number of exposed and unexposed<br>*Case-control study* - For matched studies, give matching criteria and the number of controls per case	as codes or algorithms used to identify subjects) should be listed in detail. If this is not possible, an explanation should be provided.<br><br>RECORD 6.2: Any validation studies of the codes or algorithms used to select the population should be referenced. If validation was conducted for this study and not published elsewhere, detailed methods and results should be provided.<br><br>RECORD 6.3: If the study involved linkage of databases, consider use of a flow diagram or other graphical display to demonstrate the data linkage process, including the number of individuals with linked data at each stage.	and datasets and comparative analyses sections
Variables	7	Clearly define all outcomes, exposures, predictors, potential confounders, and effect modifiers. Give diagnostic criteria, if applicable.	RECORD 7.1: A complete list of codes and algorithms used to classify exposures, outcomes, confounders, and effect modifiers should be provided. If these cannot be reported, an explanation should be provided.	Methods – Exposure, Outcome and Confounders sections.
Data sources/ measurement	8	For each variable of interest, give sources of data and details of methods of assessment (measurement).<br>Describe comparability of assessment methods if there is more than one group		Methods – study design and datasets. Appendix.
Bias	9	Describe any efforts to address potential sources of bias		Methods – study design and datasets. Sensitivity analyses in Appendix.
Study size	10	Explain how the study size was arrived at		STROBE flowcharts

				(Figures S2 and S3)
Quantitative variables	11	Explain how quantitative variables were handled in the analyses. If applicable, describe which groupings were chosen, and why		Methods – Exposure, Outcome and Confounders sections. Appendix.
Statistical methods	12	(a) Describe all statistical methods, including those used to control for confounding<br><br>(b) Describe any methods used to examine subgroups and interactions<br><br>(c) Explain how missing data were addressed<br><br>(d) *Cohort study* - If applicable, explain how loss to follow-up was addressed<br><br>*Case-control study* - If applicable, explain how matching of cases and controls was addressed<br><br>*Cross-sectional study* - If applicable, describe analytical methods taking account of sampling strategy<br><br>(e) Describe any sensitivity analyses		Methods – statistical analysis section
Data access and cleaning methods		..	RECORD 12.1: Authors should describe the extent to which the investigators had access to the database population used to create the study population.<br><br>RECORD 12.2: Authors should provide information on the data cleaning methods used in the study.	Methods Supplement (Figures S2 and S3)
Linkage		..	RECORD 12.3: State whether the study included person-level, institutional-level, or other data linkage across two or more	Methods Supplement (Figures S2 and S3)

			databases. The methods of linkage and methods of linkage quality evaluation should be provided.	
Participants	13	(a) Report the numbers of individuals at each stage of the study (*e.g.*, numbers potentially eligible, examined for eligibility, confirmed eligible, included in the study, completing follow-up, and analysed)<br>(b) Give reasons for non-participation at each stage.<br>(c) Consider use of a flow diagram	RECORD 13.1: Describe in detail the selection of the persons included in the study (*i.e.,* study population selection) including filtering based on data quality, data availability and linkage. The selection of included persons can be described in the text and/or by means of the study flow diagram.	STROBE flowchart (Figure S6 and S9)
Descriptive data	14	(a) Give characteristics of study participants (*e.g.*, demographic, clinical, social) and information on exposures and potential confounders<br>(b) Indicate the number of participants with missing data for each variable of interest<br>(c) *Cohort study* - summarise follow-up time (*e.g.*, average and total amount)		Table 1 and Supplement
Outcome data	15	*Cohort study* - Report numbers of outcome events or summary measures over time<br>*Case-control study* - Report numbers in each exposure category, or summary measures of exposure<br>*Cross-sectional study* - Report numbers of outcome events or summary measures		Table 1
Main results	16	(a) Give unadjusted estimates and, if applicable, confounder- adjusted		Appendix tables.

		estimates and their precision (e.g., 95% confidence interval). Make clear which confounders were adjusted for and why they were included<br><br>(b) Report category boundaries when continuous variables were categorized<br><br>(c) If relevant, consider translating estimates of relative risk into absolute risk for a meaningful time period		
Other analyses	17	Report other analyses done—e.g., analyses of subgroups and interactions, and sensitivity analyses		Appendix tables.
Key results	18	Summarise key results with reference to study objectives		Discussion, first paragraph
Limitations	19	Discuss limitations of the study, taking into account sources of potential bias or imprecision. Discuss both direction and magnitude of any potential bias	RECORD 19.1: Discuss the implications of using data that were not created or collected to answer the specific research question(s). Include discussion of misclassification bias, unmeasured confounding, missing data, and changing eligibility over time, as they pertain to the study being reported.	Discussion, fifth paragraph
Interpretation	20	Give a cautious overall interpretation of results considering objectives, limitations, multiplicity of analyses, results from similar studies, and other relevant evidence		Discussion, sixth and seventh paragraphs
Generalisability	21	Discuss the generalisability (external validity) of the study results		Discussion, fourth paragraph
Funding	22	Give the source of funding and the role of the funders		Acknowledge ments

		for the present study and, if applicable, for the original study on which the present article is based		
Accessibility of protocol, raw data, and programming code		..	RECORD 22.1: Authors should provide information on how to access any supplemental information such as the study protocol, raw data, or programming code.	SAP and code via github

Figure S1: Distribution of variants over time in Scotland

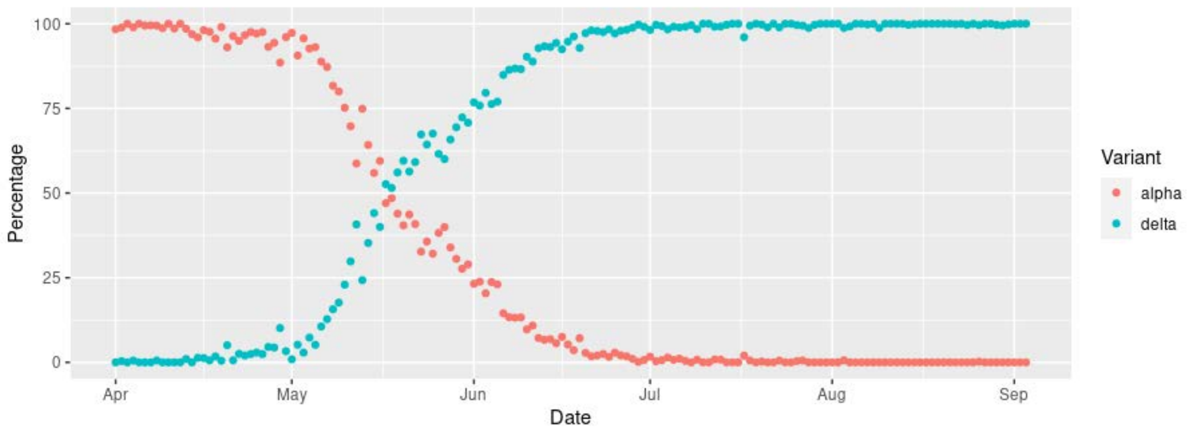

Figure S2: Data linkage diagram for Scotland

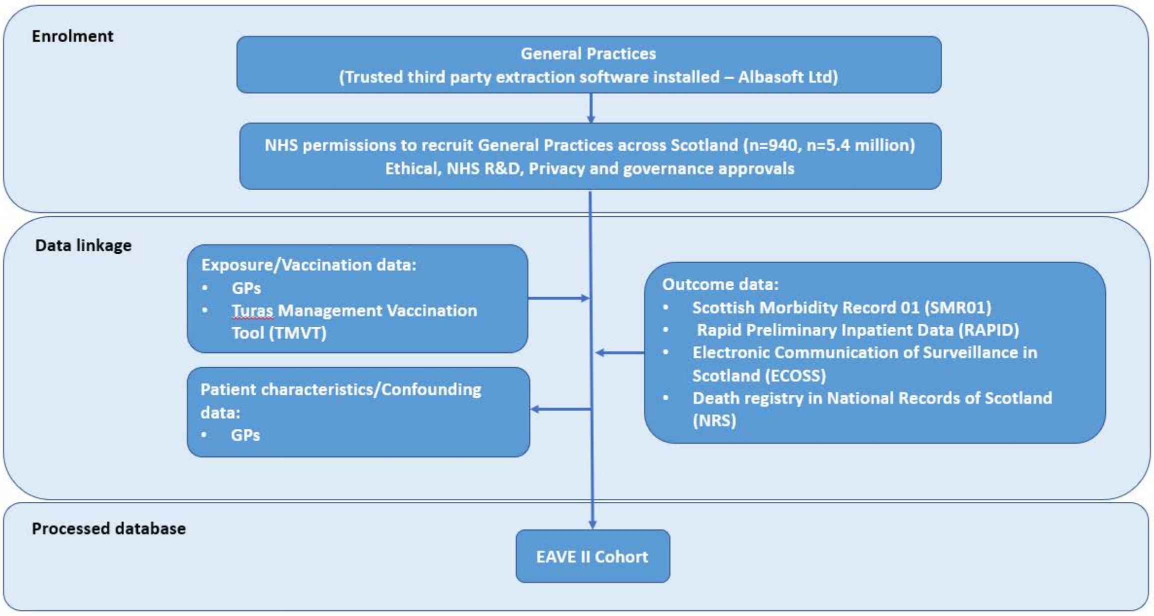

Figure adapted from Vasileiou et al.[1]

Note: Community Health Index (CHI) numbers were used to link all datasets. Details on these datasets are available in our published protocol.[2] There are two core methods of recording vaccine delivery, the national Turas Management Vaccination Tool(TMVT) system and local GP IT systems. TMVT was developed as a web application by NHS Education Scotland (NES). It is in general the preferred method of recording a vaccination where this is done outside the normal vaccine locations, predominantly dedicated vaccination centres and community programmes. Most vaccinations delivered in general practice settings are recorded in local IT systems; there are a few geographical areas that have however mandated the use of TMVT in every setting, including GP practices. Currently GPs are paid per 100 vaccines administered so they are highly motivated to record information accurately. If this is not recorded to a minimum standard, they will not receive payment. All vaccines administered through vaccination centres and community programmes are accounted for on a daily basis. All vaccines recorded via TMVT are transferred to the national clinical datastore (NCDS) then to Albasoft on a daily basis. At 9pm each night, these are loaded into a secure database and each practice “polls” the data store as part of the ESCRO data pump run between 12:00am and 5:00am each day to request the records for their specific practice. These are then loaded into a local queue at the practice for processing later in the day. As part of the same data pump run, the local GP IT system is queried and all vaccination records for the previous day are extracted (with a 10 day overlap to catch any retrospective recording) These records are then transferred back to Albasoft and collated into a single data source which is returned to the National Clinical Data Store (NCDS) at 8am each morning. As a result, all vaccinations recorded either by TMVT or GP IT systems pass through Albasoft in a 24-hour cycle. As part of the agreement to provide these data for EAVE II, vaccination records from both the TMVT and GP IT systems are transferred each day following the National Clinical Data Store processing to the EAVE II secure datastore in Public Health Scotland (PHS). This ensures that the EAVE II data are as up to date as possible. It is therefore extremely unlikely that any vaccinations will have been missed.

Additional methodological details about Scottish databases

The Early Pandemic Evaluation and Enhanced Surveillance of COVID-19 (EAVE II) study brings together data from 5.4 million people in Scotland covering ~99% of the national population.[2] Primary care data from 940 general practices were linked to laboratory data from the Electronic Communication of Surveillance in Scotland (ECOSS), hospital discharge data from the Scottish Morbidity Record (SMR) 01 database and Rapid Preliminary Inpatient Data (RAPID),[3] and the death registry from National Records of Scotland (NRS). Vaccination status was ascertained from general practice records and the Turas Vaccination Management Tool (TVMT).[4] ECOSS data included all reverse transcriptase polymerase chain reaction (RT-PCR) test results from both NHS laboratories (Pillar 1 which included high proportions of asymptomatic screening tests) and Lighthouse Government laboratories (Pillar 2, largely symptomatic community tests).[16] Data were deterministically linked using the Community Health Index (CHI) unique identifier.[13] Approvals were obtained from the National Research Ethics Service Committee, Southeast Scotland 02 (reference number: 12/SS/0201), and Public Benefit and Privacy Panel for Health and Social Care (reference number: 1920-0279).

Figure S3: Data linkage diagram for Brazil

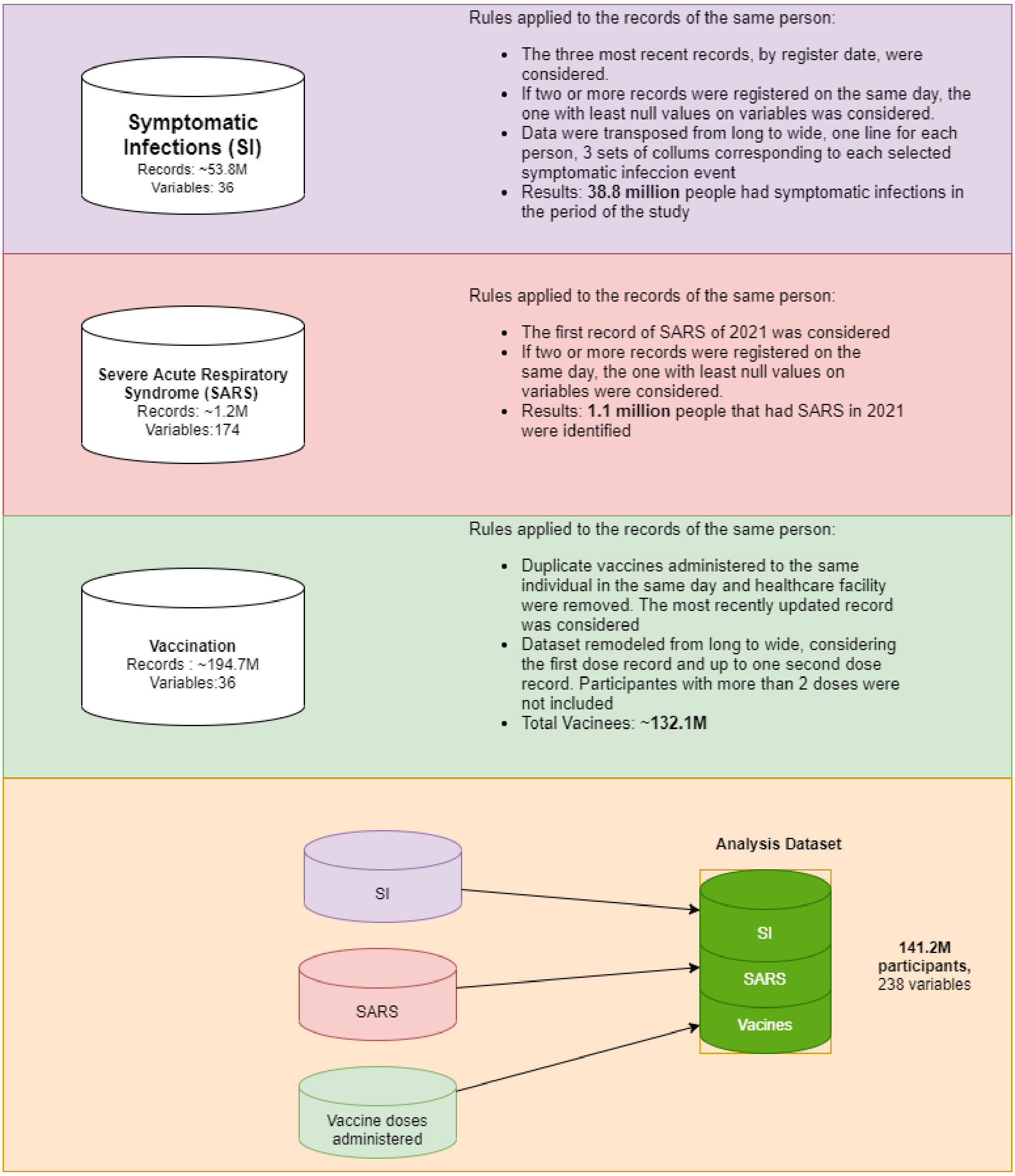

Additional methodological details about Brazilian databases

In Brazil, we used three deterministically linked national datasets provided by the Brazilian Ministry of Health Department of Informatics (DATASUS): COVID-19 Vaccination Campaign (SI-PNI) containing all administered vaccine doses; Acute Respiratory Infection Suspected Cases (e-SUS-Notifica) which holds clinical and laboratory data from all suspected cases and contact tracing; and Severe Acute Respiratory Infection/Illness (SIVEP-Gripe) containing all COVID-19 hospitalisations and deaths. The Brazilian National Commission in Research Ethics approved the research protocol (CONEP approval number 4.921.308). All analyses used unidentified secondary data in accordance with the Brazilian

Personal Data Protection General Law (LGPD). Data were manipulated in a secure computing environment, ensuring protection against data leakage and records re-identification.

Figure S4: Distribution of variants over time in Brazil, stratified by region

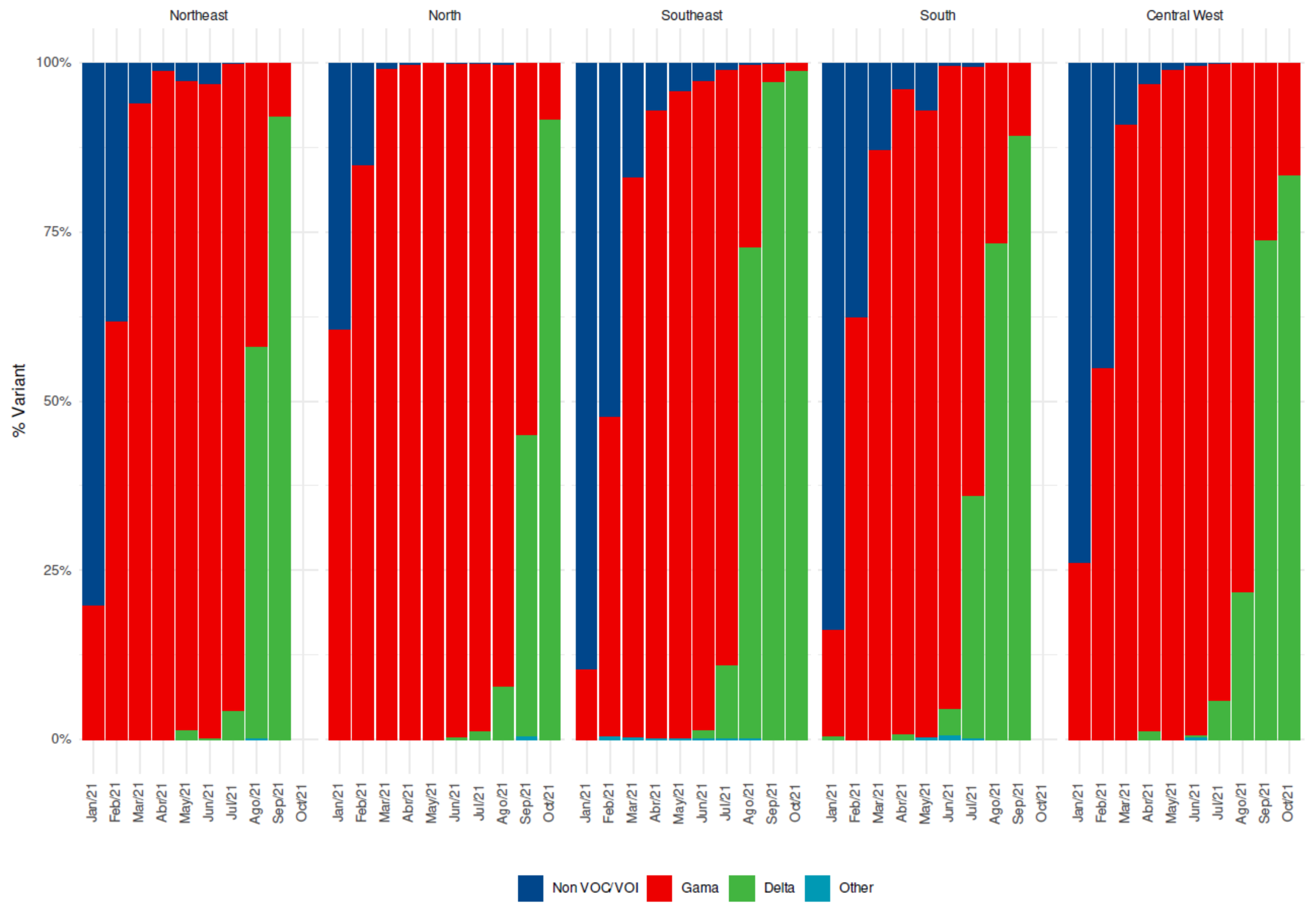

Figure S5: ChAdOx1 two-dose vaccination numbers in Scotland, stratified by age group

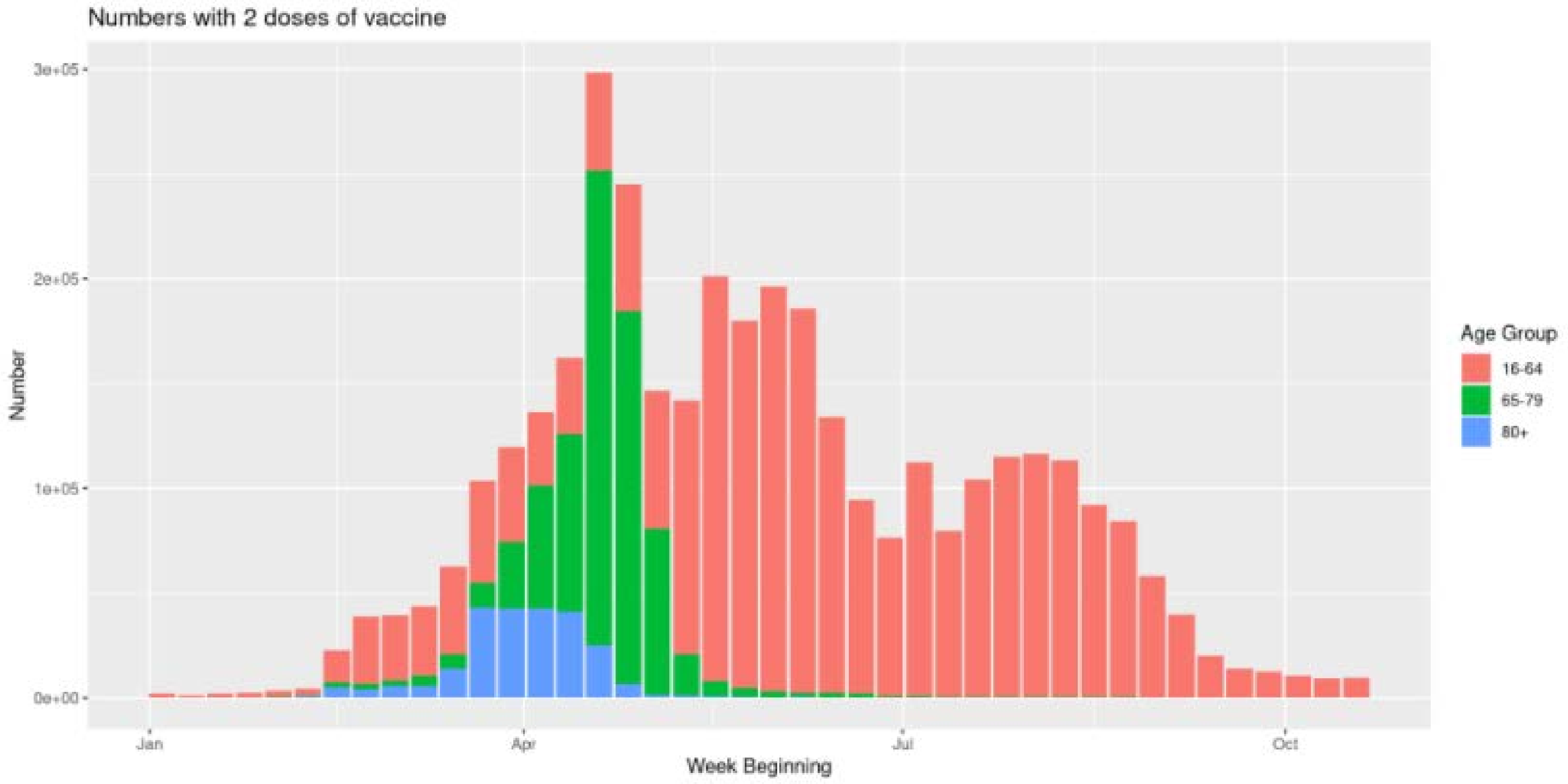

Figure S6: STROBE Flow diagram for Scotland

Scotland – 2 Dose ChAdOx1 cohort

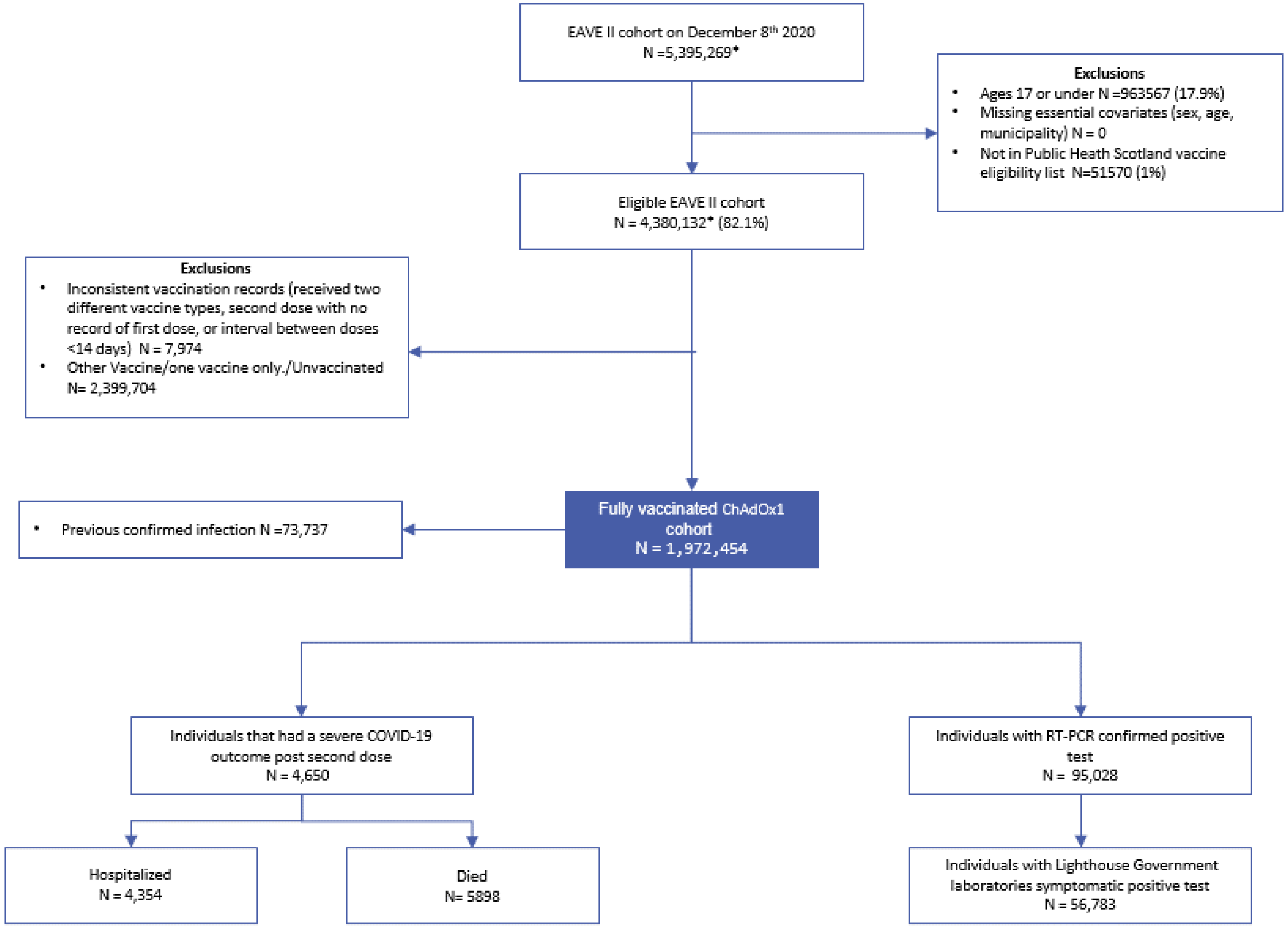

Scotland – ChAdOx1 Vaccine Effect Cohort

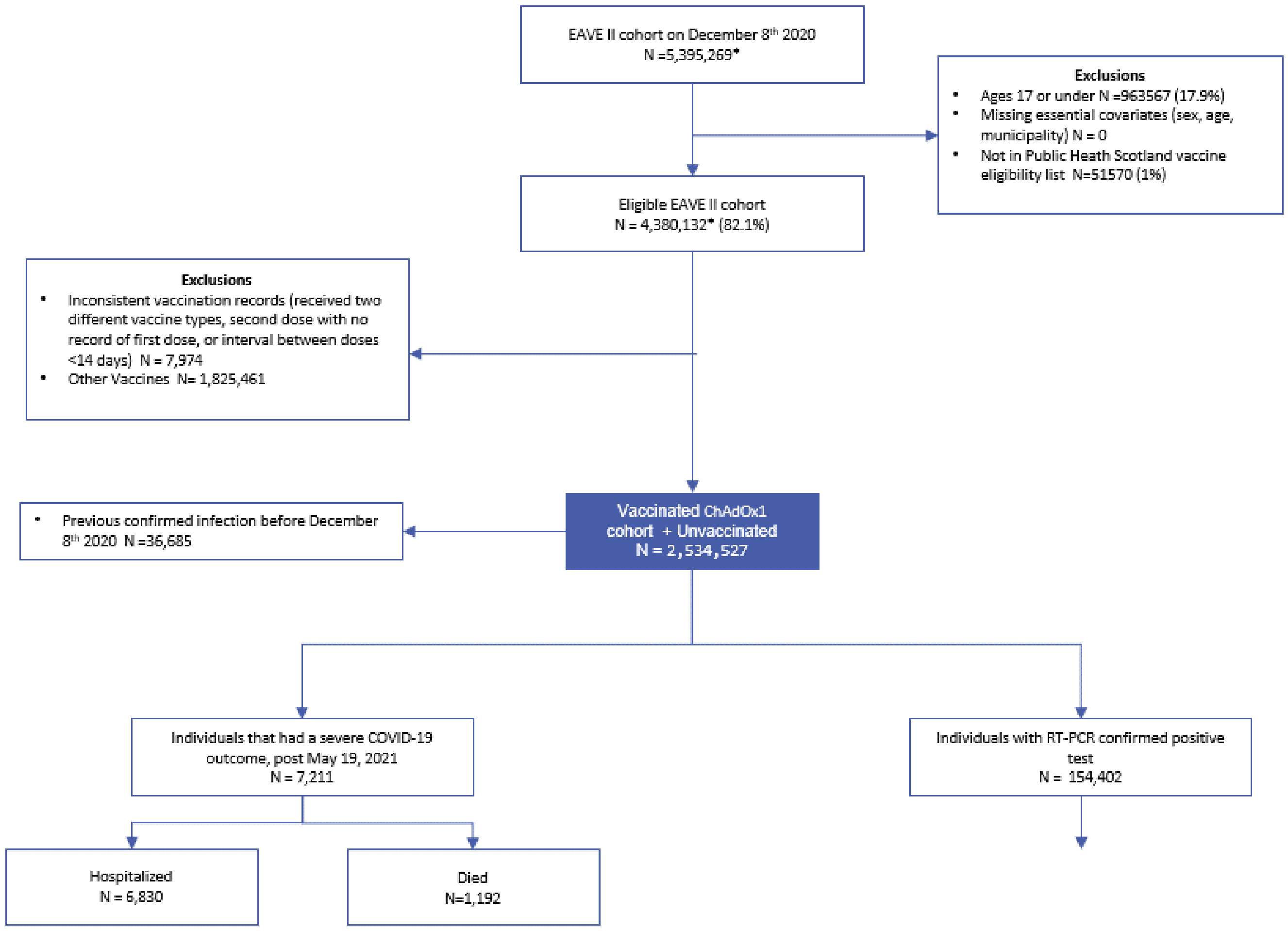

Based upon sampling weights, as the number of records in EAVE II is greater than the population of Scotland

Figure S7: Trends in COVID-related outcomes in Scotland for: a) COVID-19 hospitalisation or death; b) COVID-19 hospitalisation; c) COVID-19 death; d) SARS-CoV-2 confirmed infection

a) COVID-19 hospitalisation or death

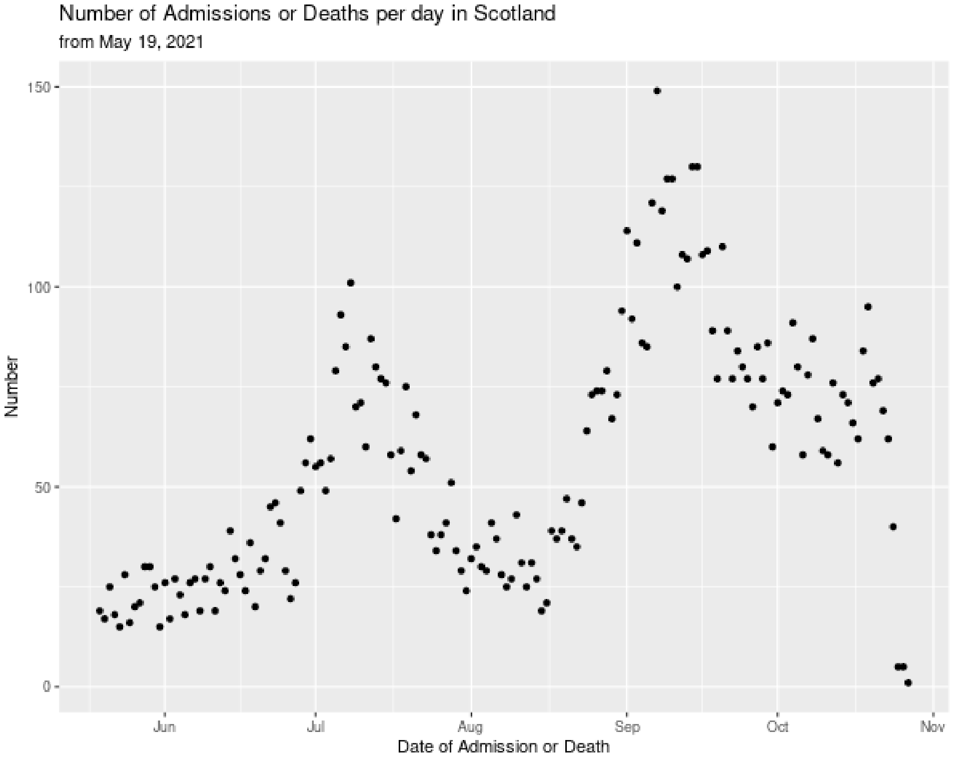

b) COVID-19 hospitalisation

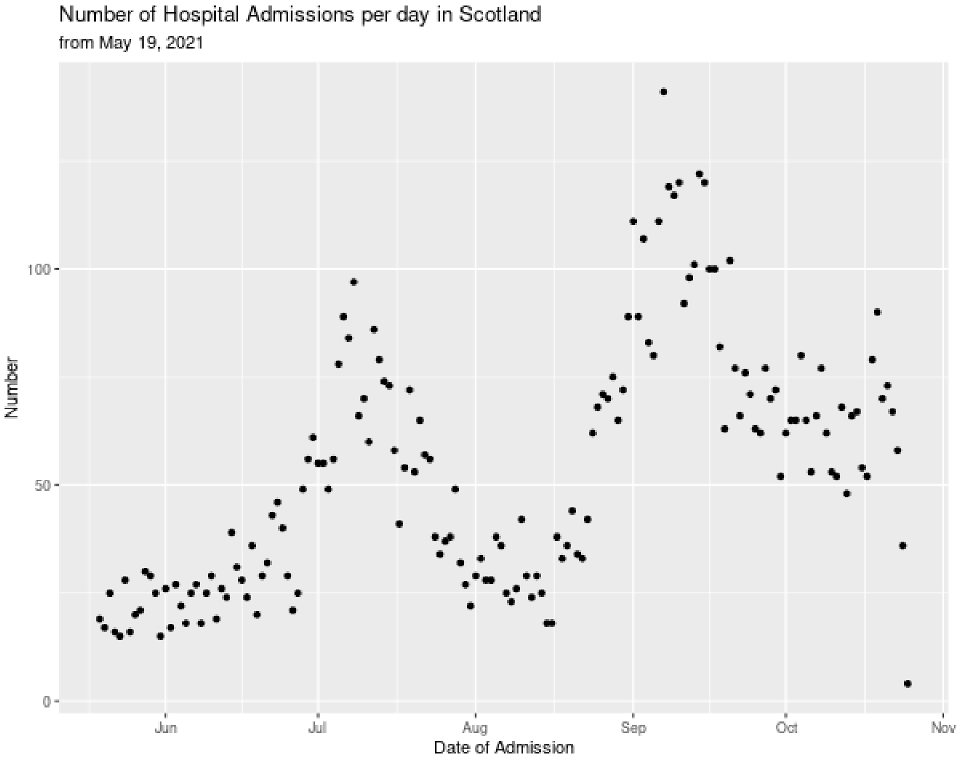

c) COVID-19 death

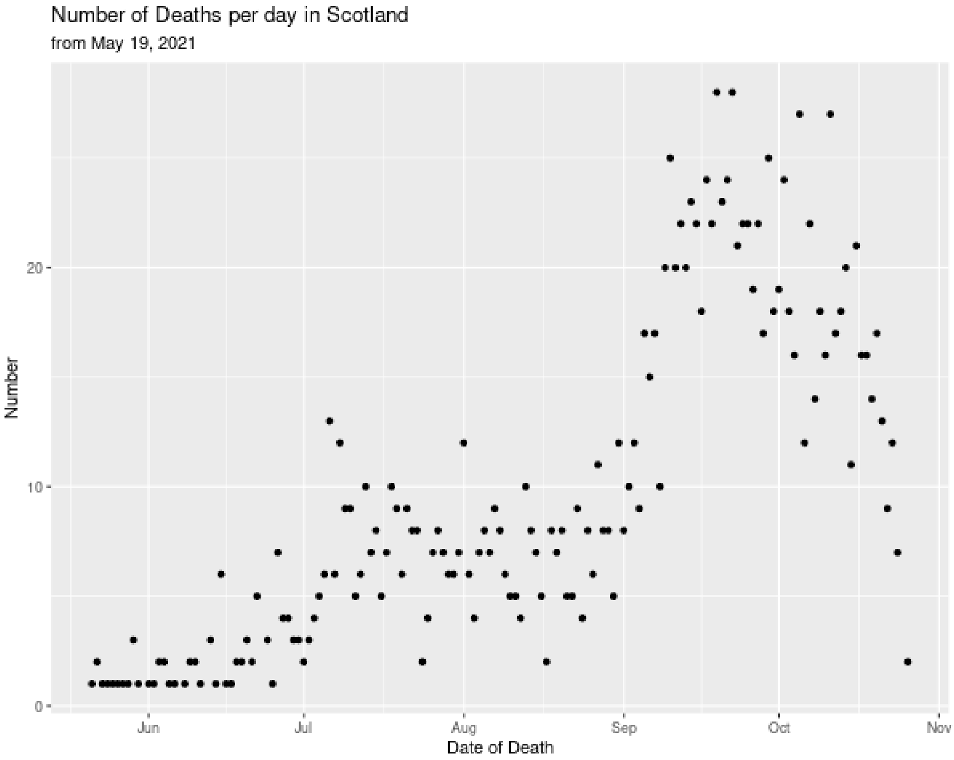

d) SARS-CoV-2 confirmed infection

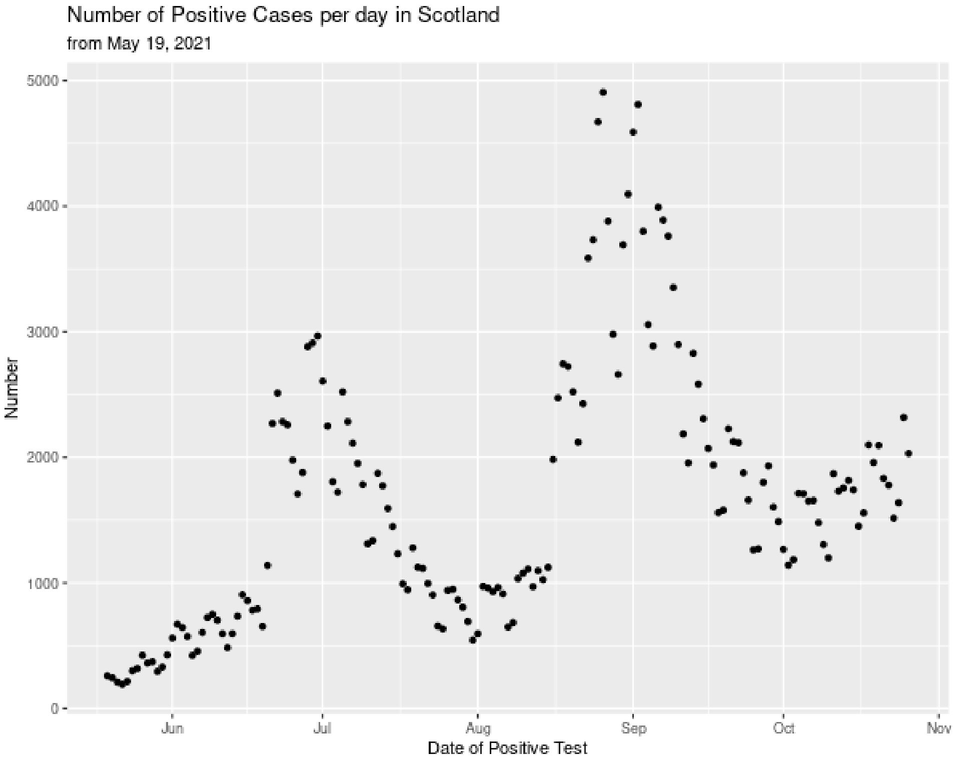

Figure S8: Association between waning and calendar period effects for vaccines aged 65+ in Scotland

Most individuals aged 65+ received the ChadOx1 vaccine and virtually all were vaccinated in the period between March 23, 2021 and May 18, 2021, with 330,000 vaccinated in a three-week period (April 14-May 4 2021). The waning period and the calendar period are confounded with the week of vaccination through the age-period cohort paradigm where the week of receiving the second dose represents a cohort of individuals. The use of two-week periods for the vaccine waning partially breaks the linear association but does not completely eliminate it.

This issue can be seen in the table below where the table entries are the post vaccine periods. Within the study period short post vaccine periods were not observed for the majority of the vaccinees. The periods of peak infections are identified by the shading and these correspond to post vaccine periods 4 and 5 and 8-10 for the vaccine cohorts which were the largest groups. Information on the long post vaccination periods 10-12, are also only available within the second peak period.

Period	Vaccination Week							
	24/03/21	31/03/21	07/04/21	14/04/21	21/04/21	28/04/21	05/05/21	12/05/21
19/05/21	4	3	3	2	2	1	1	0
26/05/21	4	4	3	3	2	2	1	1
02/06/21	5	4	4	3	3	2	2	1
09/06/21	5	5	4	4	3	3	2	2
16/06/21	6	5	5	4	4	3	3	2
23/06/21	6	6	5	5	4	4	3	3
30/06/21	7	6	6	5	5	4	4	3
07/07/21	7	7	6	6	5	5	4	4
14/07/21	8	7	7	6	6	5	5	4
21/07/21	8	8	7	7	6	6	5	5
28/07/21	9	8	8	7	7	6	6	5
04/08/21	9	9	8	8	7	7	6	6
11/08/21	10	9	9	8	8	7	7	6
18/08/21	10	10	9	9	8	8	7	7
25/08/21	11	10	10	9	9	8	8	7
01/09/21	11	11	10	10	9	9	8	8
08/09/21	12	11	11	10	10	9	9	8

Within each of the vaccine cohorts it is difficult to segregate the trends in the post vaccine period from the calendar period trend. Changes in trend were identified. In the graphs below the event rates for hospitalisation or death are shown by calendar period and by post vaccine period for the seven weekly vaccine cohorts. In the first chart the temporal trend can be seen with an approximate linear increase in rates up to the first peak then a decline followed by another increase. In the second chart there is a linear trend for increasing hospitalisation rates with increasing post vaccine period and this linear trend cannot be separated out from the temporal trend in this age group.

A similar situation exists for the 80+ age group. However, for those aged under 65, sufficient numbers entered the vaccinated cohort from the beginning of April onwards that there is representation of all post vaccine periods from 0-1 weeks post vaccination to 19-20 weeks post vaccination in all calendar periods during the study period.

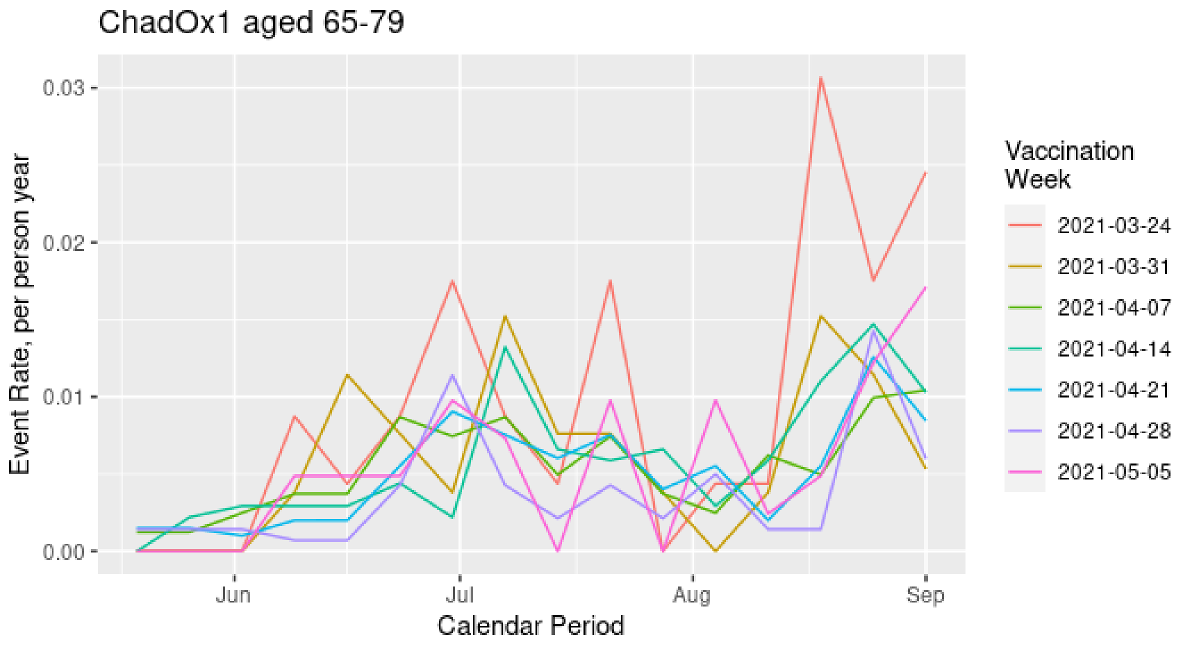

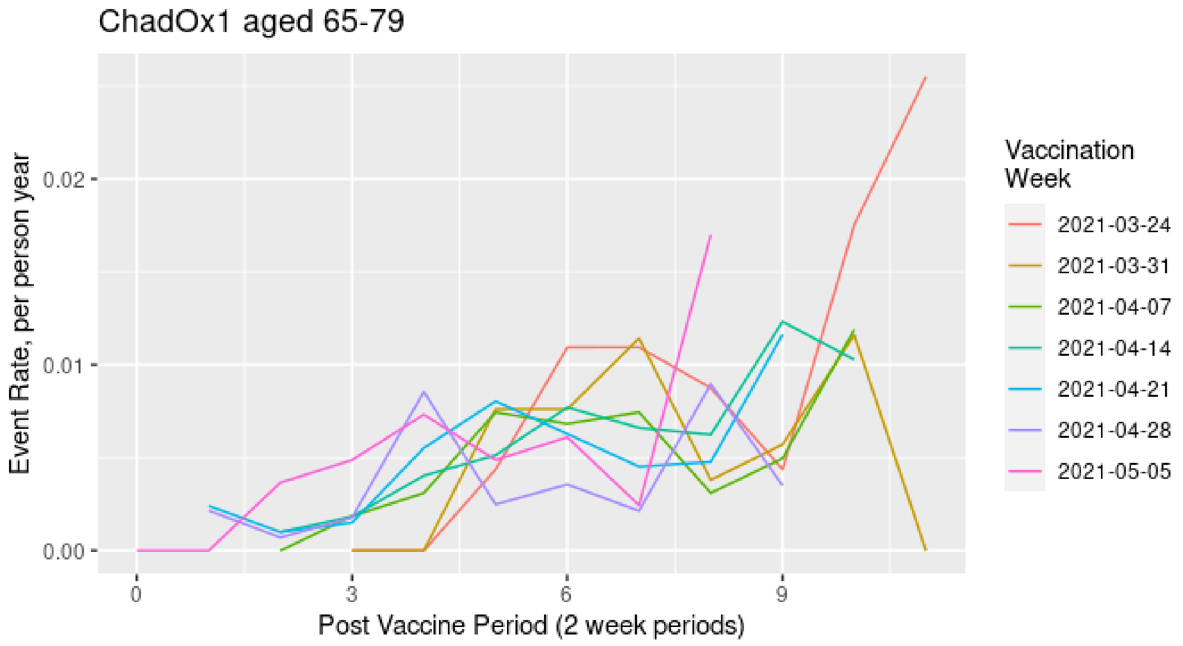

Figure S9: STROBE Flow diagram for Brazil

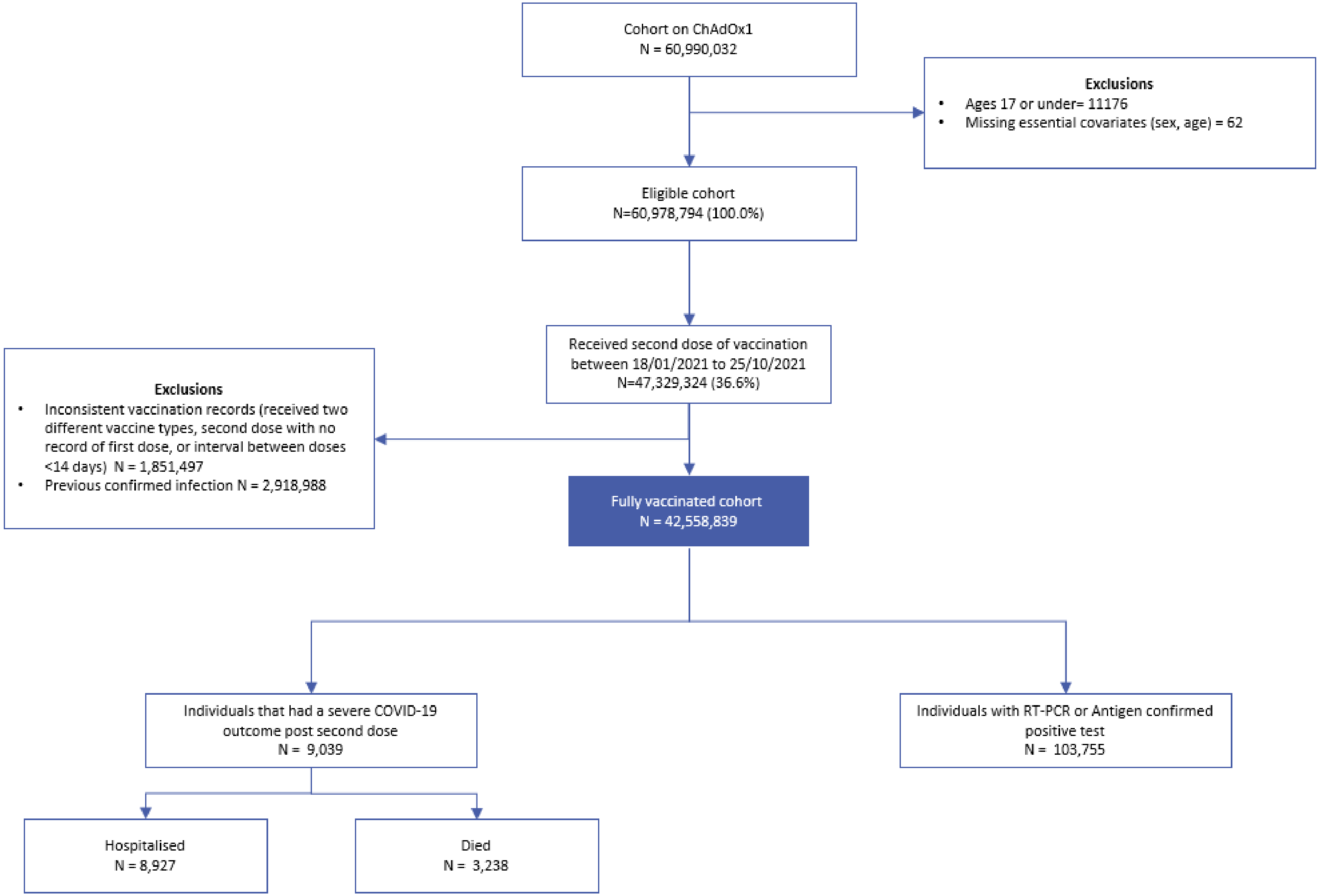

Figure S10: Monthly numbers of: a) COVID-19 hospitalisation or death; b) COVID-19 hospitalisation; c) COVID-19 death; d) SARS-CoV-2 confirmed symptomatic infection in Brazil, stratified by age group

A)

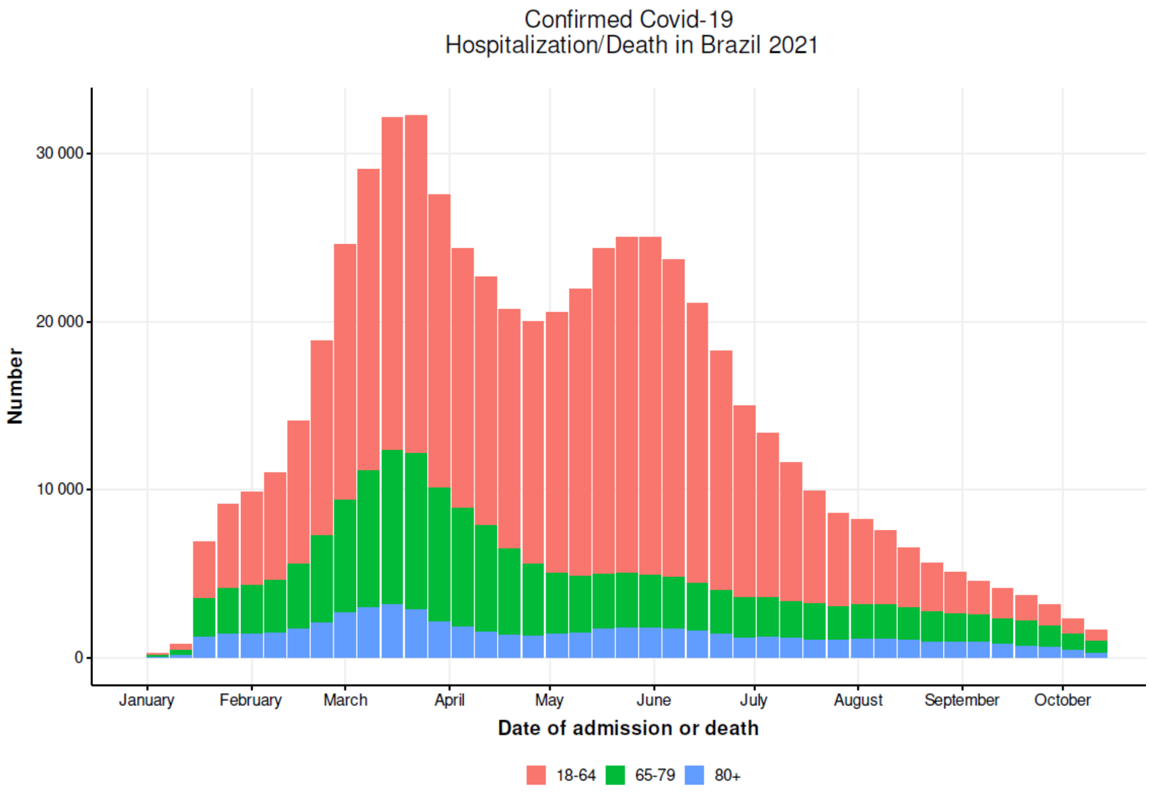

B)

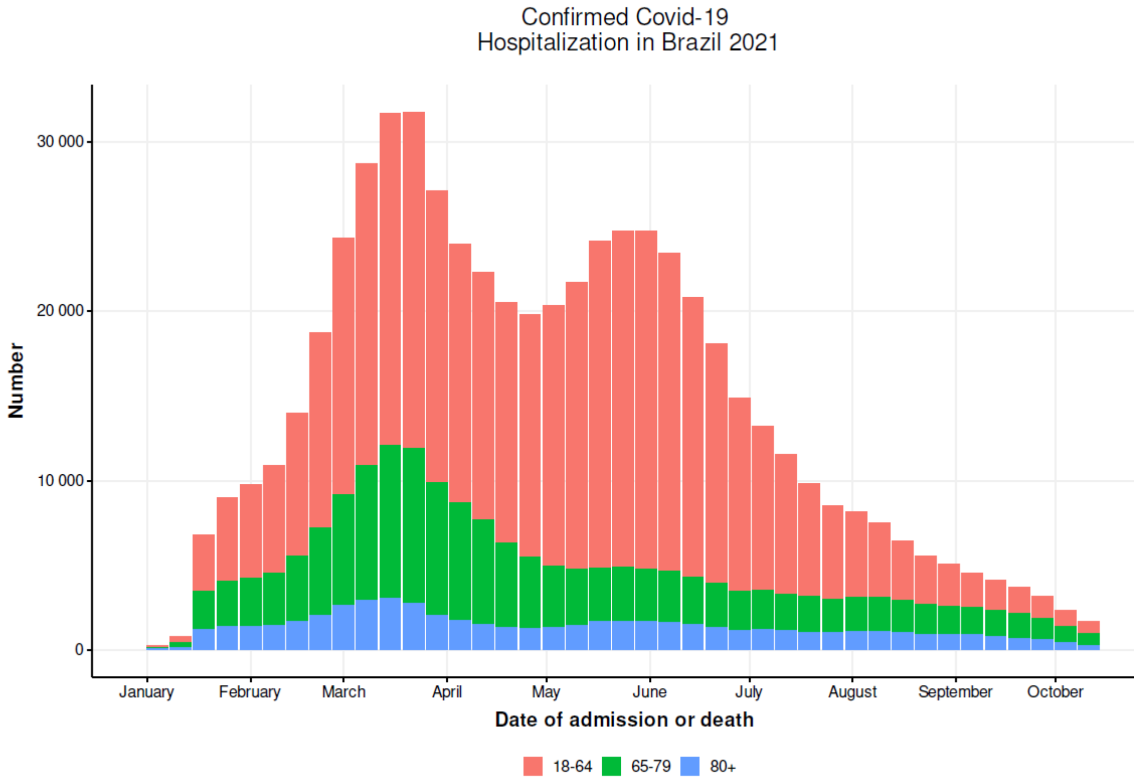

C)

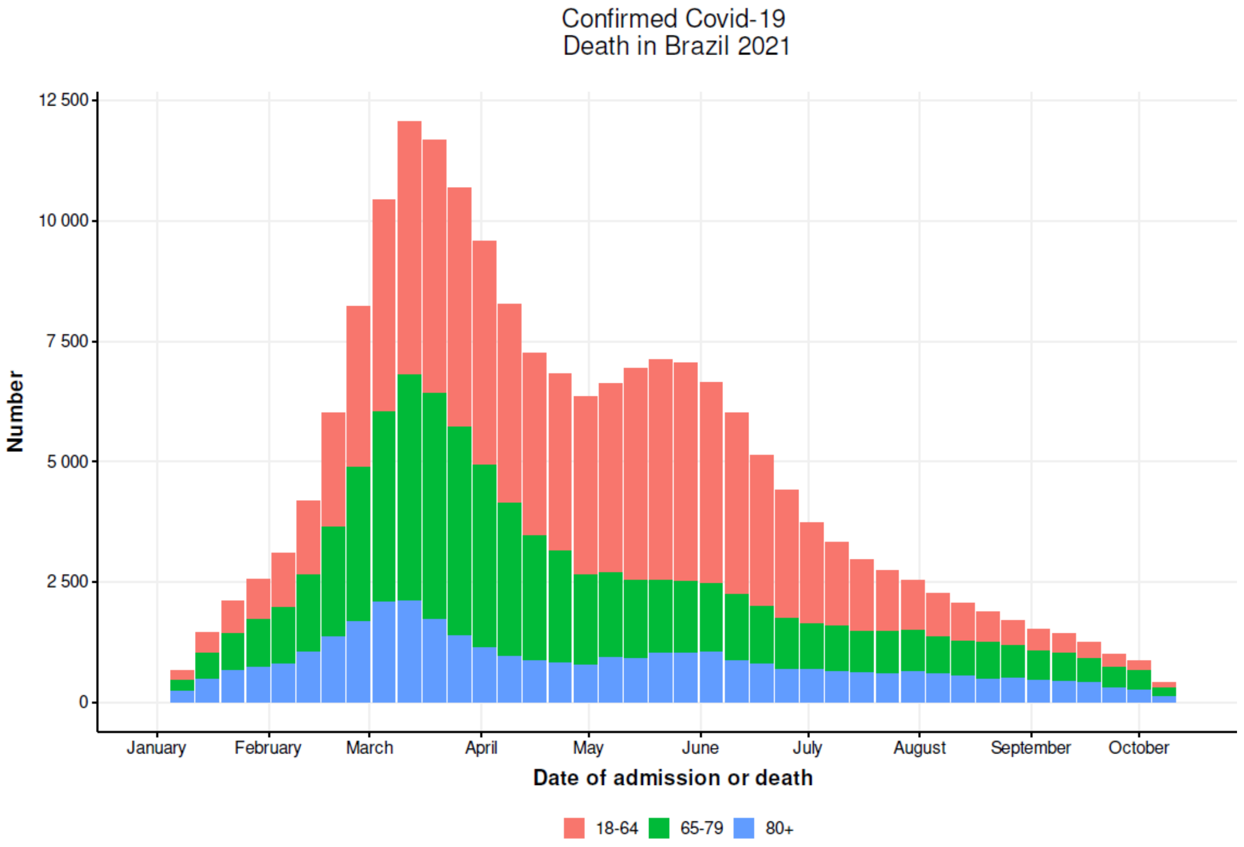

Confirmed Covid-19
Death in Brazil 2021
12 500
10 000
7 500
5 000
2 500
0
Number
January
February
March
April
May
June
July
August
September
October
Date of admission or death
18-64
65-79
80+

D)

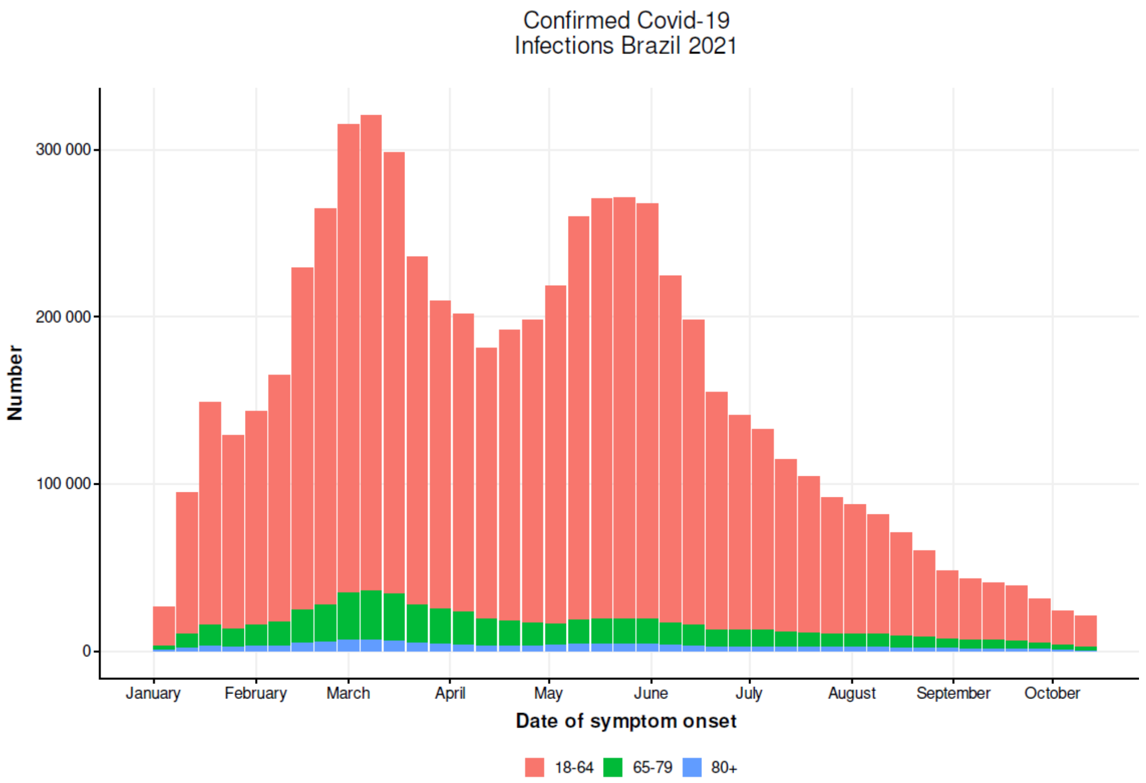

Confirmed Covid-19
Infections Brazil 2021
300 000
200 000
100 000
0
Number
January
February
March
April
May
June
July
August
September
October
Date of symptom onset
18-64
65-79
80+

Figure S11: Cumulative uptake for receipt of any vaccination in Brazil, stratified by age group

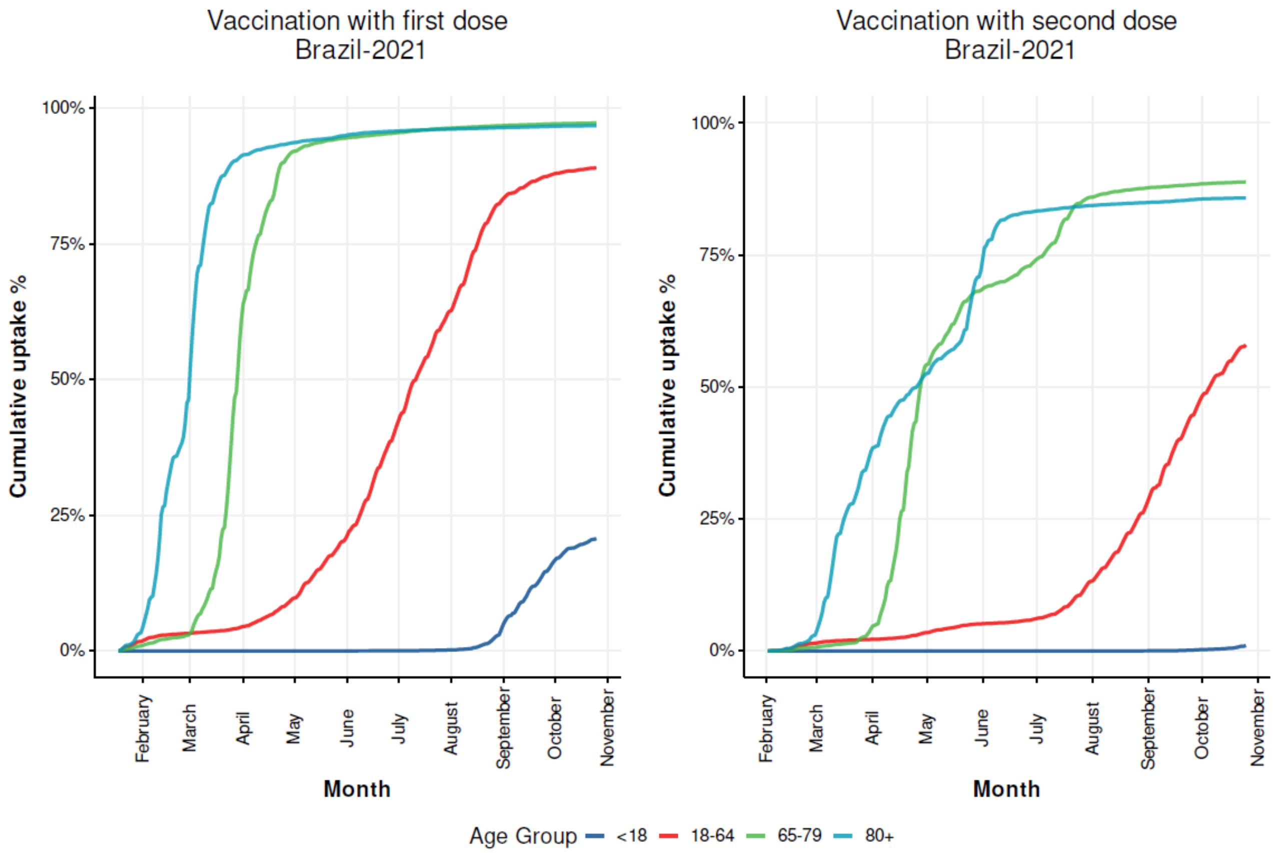

Figure S12: ChAdOx1 vaccination numbers in Brazil, stratified by age group

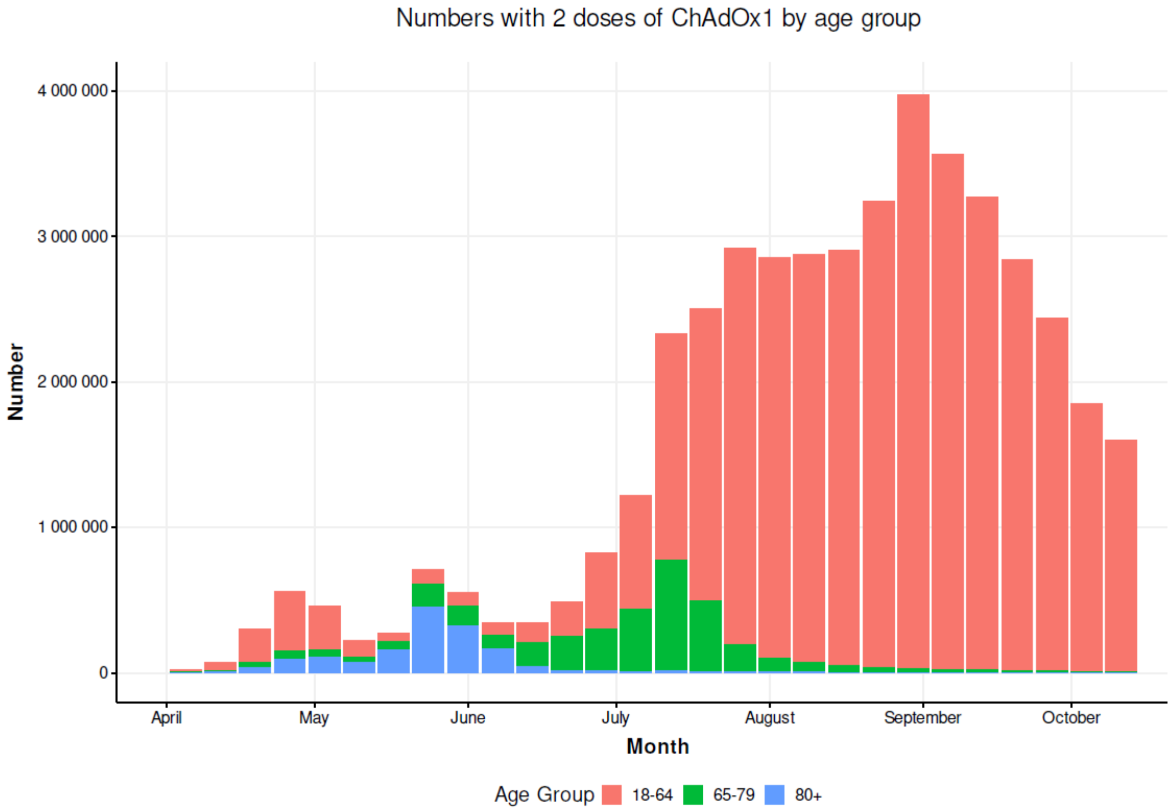

References

1. Vasileiou E, Simpson CR, Shi T, et al. Interim findings from first-dose mass COVID-19 vaccination roll-out and COVID-19 hospital admissions in Scotland: a national prospective cohort study. The Lancet 2021;397:1646-57.
2. Simpson CR, Robertson C, Vasileiou E, et al. Early Pandemic Evaluation and Enhanced Surveillance of COVID-19 (EAVE II): protocol for an observational study using linked Scottish national data. BMJ Open 2020;10:e039097.
3. National Data Catalogue. Rapid Preliminary Inpatient Data (RAPID). 2021. (Accessed 14/3/2021, at https://www.ndc.scot.nhs.uk/National-Datasets/data.asp?SubID=37.)
4. Turas Vaccine Management Tool. 2021. (Accessed 14/3/2021, at https://learn.nes.nhs.scot/42708/turas-vaccination-management-tool.)
